# RASSF1C oncogene elicits amoeboid invasion, cancer stemness, and extracellular vesicle release via a SRC/Rho axis

**DOI:** 10.15252/embj.2021107680

**Published:** 2021-09-17

**Authors:** Maria Laura Tognoli, Nikola Vlahov, Sander Steenbeek, Anna M Grawenda, Michael Eyres, David Cano‐Rodriguez, Simon Scrace, Christiana Kartsonaki, Alex von Kriegsheim, Eduard Willms, Matthew J Wood, Marianne G Rots, Jacco van Rheenen, Eric O'Neill, Daniela Pankova

**Affiliations:** ^1^ Department of Oncology University of Oxford Oxford UK; ^2^ Molecular Pathology Oncode Institute The Netherlands Cancer Institute Amsterdam The Netherlands; ^3^ University of Groningen University Medical Center Groningen Groningen The Netherlands; ^4^ Cancer Research UK Edinburgh Centre MRC Institute of Genetics & Molecular Medicine The University of Edinburgh Western General Hospital Edinburgh UK; ^5^ Department of Physiology, Anatomy and Genetics University of Oxford Oxford UK; ^6^ La Trobe Institute for Molecular Science La Trobe University Melbourne Vic. Australia; ^7^ Department of Paediatrics University of Oxford Oxford UK

**Keywords:** amoeboid motility, extracellular vesicles, gene methylation, RASSF1C oncogene, Rho/ROCK pathway, Cancer, Cell Adhesion, Polarity & Cytoskeleton, Signal Transduction

## Abstract

Cell plasticity is a crucial hallmark leading to cancer metastasis. Upregulation of Rho/ROCK pathway drives actomyosin contractility, protrusive forces, and contributes to the occurrence of highly invasive amoeboid cells in tumors. Cancer stem cells are similarly associated with metastasis, but how these populations arise in tumors is not fully understood. Here, we show that the novel oncogene RASSF1C drives mesenchymal‐to‐amoeboid transition and stem cell attributes in breast cancer cells. Mechanistically, RASSF1C activates Rho/ROCK via SRC‐mediated RhoGDI inhibition, resulting in generation of actomyosin contractility. Moreover, we demonstrate that RASSF1C‐induced amoeboid cells display increased expression of cancer stem‐like markers such as CD133, ALDH1, and Nanog, and are accompanied by higher invasive potential *in vitro* and *in vivo*. Further, RASSF1C‐induced amoeboid cells employ extracellular vesicles to transfer the invasive phenotype to target cells and tissue. Importantly, the underlying RASSF1C‐driven biological processes concur to explain clinical data: namely, methylation of the RASSF1C promoter correlates with better survival in early‐stage breast cancer patients. Therefore, we propose the use of *RASSF1* gene promoter methylation status as a biomarker for patient stratification.

## Graphical abstract

Tumour‐suppressor RASSF1A encodes an alternative transcript, RASSF1C, that acts as oncogene in human breast cancer, triggering pro‐invasive cellular features and aggressiveness.

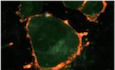

## Introduction

Cell invasion and migration are essential processes during cancer progression and metastatic dissemination. In different tissue contexts, cancer cells use distinct modes of invasion, moving either as collective groups or single cells, adopting mesenchymal or amoeboid motility (Friedl & Bröcker, 2000; Friedl *et al*, 2012). Mesenchymally invading single cells use integrins and metalloprotease‐dependent degradation of the extracellular matrix to invade and metastasize to distant organs. Downregulation of integrins or inhibition of metalloproteases results in mesenchymal‐amoeboid transition (MAT) and adoption of amoeboid type of invasion. Amoeboid cancer cells are characterized by upregulation of Rho/ROCK signaling pathway, which drives reorganization of the actin cytoskeleton and leads to generation of protrusive forces. It has been reported that enhanced Rho/ROCK signaling directly promotes phosphorylation of myosin light chain 2 (pMLCII) *in vitro* (Kimura *et al*, 1996), which in turn induces reduction of stress fibers and formation of cortical actomyosin (Kimura *et al*, 1996; Álvarez‐González *et al*, 2015). These major changes in actin architecture enable cancer cells to remodel components of the extracellular matrix and to squeeze into the surrounding tissue. Interestingly, invading cells can employ a hybrid mode of motility, switching between MAT and amoeboid‐mesenchymal transition (AMT), depending on the tissue specificity of the matrices (Egeblad *et al*, 2010; He *et al*, 2011). This cellular plasticity allows cancer cells to initiate a lesion and adopt appropriate invasive modes for each step of the metastatic process. Cancer dissemination is a complex mechanism, and the exact sequence of molecular events that lead to metastatic colonization of distant sites of the body is not well understood. Extracellular vesicles (EVs) have recently been demonstrated to influence epithelial‐mesenchymal transition (EMT) and migration in both an autocrine and non‐cell autonomous manner (Luga *et al*, 2012; Sung *et al*, 2015). Extracellular vesicles are small lipid bilayer vesicles released from internal compartments such as multivesicular bodies (exosomes) or via blebbing from the plasma membrane (ectosomes, microvesicles) and have been implicated in promoting metastasis (Peinado *et al*, 2012; Hoshino *et al*, 2015). Extensive membrane blebbing facilitated by Rho‐driven motility has been associated with increased shedding of vesicles (Li *et al*, 2012; Paluch & Raz, 2013; Sedgwick *et al*, 2015). It has recently been reported that RhoA‐driven tumor progression is attributed to loss of the Hippo pathway scaffold and tumor suppressor RASSF1A (Lee *et al*, 2016). Epigenetic suppression of the *RASSF1‐1α* promoter has been associated with poor cancer survival (Pefani *et al*, 2014, 2016) due to suppression of RASSF1A transcript expression. However, this event also represents an epigenetic switch and expression of an alternative isoform, RASSF1C, from the internal *RASSF1‐2γ* promoter (Vlahov *et al*, 2015). Although methylation of the *RASSF1* gene has been investigated in a large number of clinical studies and has been considered as a potential biomarker for breast cancer progression (Grawenda & O’Neill, 2015), how methylation of *RASSF1‐2γ* influences disease outcome has not been addressed. RASSF1C has been shown to activate SRC kinase and induce an invasive phenotype, both *in vitro* and *in vivo* (Reeves *et al*, 2010; Vlahov *et al*, 2015). SRC plays a crucial role in cancer cell plasticity and has been described as a key player in EMT in solid tumors, via its association with FAK and β‐integrins (Canel *et al*, 2010). SRC can also regulate small Rho‐GTPases by phosphorylation of RhoGDI (DerMardirossian *et al*, 2006). Here we identified a novel mechanism where SRC promotes amoeboid invasiveness via RASSF1C‐SRC‐RhoGDI signaling and Rho/ROCK/pMLCII activation. We show that SRC activation by RASSF1C leads to inhibitory phosphorylation of RhoGDI, followed by ROCK/Rho upregulation, which in turn drives MAT. We also demonstrate that molecular events driven by MAT are associated with cancer stemness and that amoeboid cells express the cancer stem cell markers ALDH1, CD133, and the pluripotency marker Nanog. Additionally, our data illustrate that RASSF1C‐SRC‐Rho activity results in release of EVs that transfer stemness, invasive ability, and metastatic behavior to recipient cells *in vitro* and *in vivo*.

Detailed analysis of the *RASSF1* promoter (*RASSF1‐1α* vs *RASSF1‐2γ*) in early‐stage breast cancer supports the association of RASSF1C with adverse prognosis and offers an optimized biomarker for patients.

## Results

### The RASSF1C oncogene promotes mesenchymal‐amoeboid transition

**Figure EV1:**
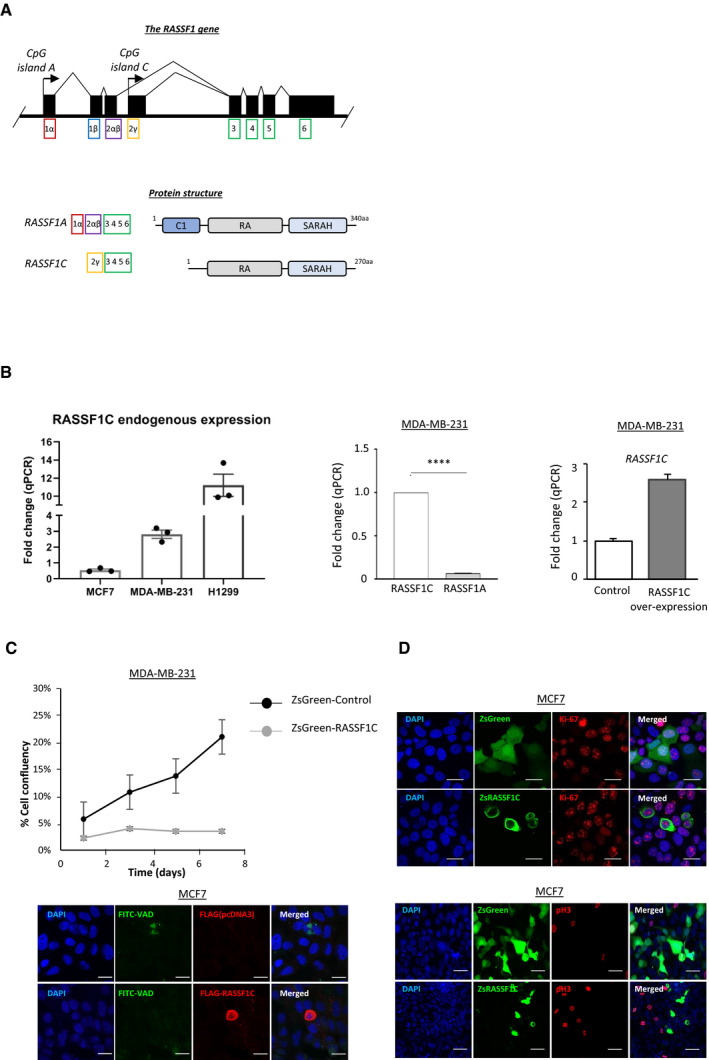
RASSF1C oncogene is responsible for MAT Schematic representation of the *RASSF1* gene locus (top) and protein domains of the two main *RASSF1* isoforms (bottom). Top: black boxes represent exons, black arrows represent CpG island promoters. Bottom, RASSF1A transcript contains six exons (1α, 2αβ, 3, 4, 5, and 6) and is translated to a protein with 340 aa. The RASSF1C variant is transcribed from an intragenic CpG island and consists of five exons (2γ, 3, 4, 5, and 6) and is translated to a protein of 270 aa. Bottom: at the protein level, RASSF1A and RASSF1C both encode for a RA (Ras Association) domain and a C‐terminal SARAH (Sav/RASSF/Hpo) domain. RASSF1C however lacks the C1 (protein C kinase conserved) region at the N‐terminal, which is present in RASSF1A.Left: fold change of qRT–PCR for endogenous RASSF1C expression in the cells lines used in this study is shown. Middle: fold change of qRT–PCR for RASSF1C and RASSF1A mRNA in MDA‐MB‐231 cells are shown. Right: MDA‐MB‐231 cells were transfected with a Control (pcDNA3) or FLAG‐RASSF1C plasmid and mRNA levels of RASSF1C were analyzed via qRT–PCR. Data show a 2.5‐fold increase in RASSF1C mRNA upon over‐expression.MDA‐MB‐231 cells stably expressing either a ZsGreen‐Control or ZsGreen‐RASSF1C construct were FACS sorted for green^high^ expression, re‐seeded, and kept in 2D culture for the following 7 days. Confluency was measured by taking a picture every other day for 7 days and quantifying the amount of green fluorescent cells.Confocal images of MCF7 cells transiently transfected with ZsGreen or Flag constructs and stained after 72 h for Ki‐67 (red) expression, FITC‐VAD (green), and Flag (red), phospho‐Histone 3 (red) and DAPI (blue). Scale bars represent 10 μm (Ki‐67 and FITC‐VAD panels) and 20 μm (pH3 panel). Data information: Data are analyzed by Student’s *t*‐test and represented as mean ± SEM. ****P ≤ 0.0001, *n* = 3. Source data are available online for this figure.

RASSF1C oncogene is one of the two main isoforms encoded by the *RASSF1* gene (EV1A). The major isoform, RASSF1A, is a *bona fide* tumor suppressor epigenetically inactivated in numerous cancers (lung and breast cancer, among others (Grawenda & O’Neill, 2015)). Surprisingly, RASSF1C expression is maintained in tumors and emerging studies indicate a pro‐oncogenic role for this isoform of the *RASSF1* gene (Estrabaud *et al*, 2007). We have previously linked RASSF1C to SRC kinase activation and aggressiveness in breast cancer, contributing to explain the biological events leading to the aggressive phenotype observed in *RASSF1*A‐methylated tumors (Vlahov *et al*, 2015). To study the effect of RASSF1C oncoprotein (EV1A) in breast cancer, we employed MCF7 and MDA‐MB‐231 cell lines, both lacking expression of the tumor suppressor isoform RASSF1A, as shown in reports from our and other groups (Montenegro *et al*, 2012; Vlahov *et al*, 2015; Calanca *et al*, 2019; Chatzifrangkeskou *et al*, 2019). We first assessed RASSF1C transcript endogenous levels in both breast and H1299 lung cancer cell lines (Fig EV1B, left). The mRNA levels of RASSF1C were significantly higher in MDA‐MB‐231 cells compared to RASSF1A transcript levels (Fig EV1B, middle), in agreement with reports indicating loss of RASSF1A expression in this cell line. Additionally, we confirmed expression levels of RASSF1C mRNA after MDA‐MB‐231 cells were transfected with a plasmid encoding for a control sequence (pcDNA3) or RASSF1C (Fig EV1B, right graph). Importantly, upon RASSF1C over‐expression, cells remained viable, despite showing lower proliferation rates than controls (Fig EV1C), and displayed markers of proliferation (Ki‐67, Fig EV1D) but not markers of apoptosis (FITC‐VAD, Fig EV1D) or active mitosis (pH3, Fig EV1D).

**Figure 1:**
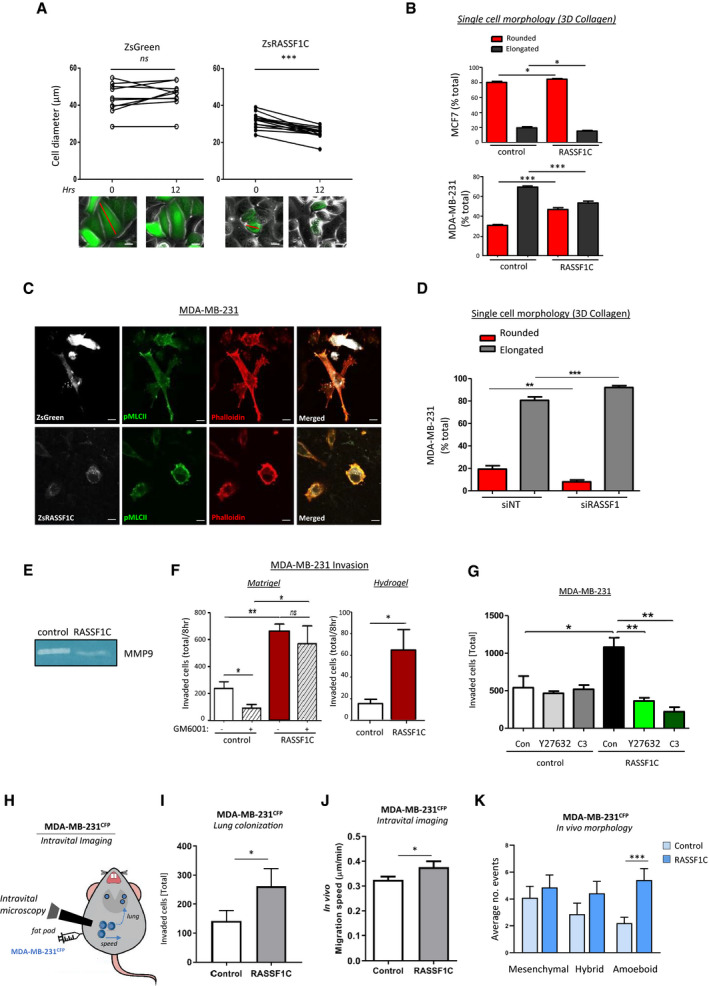
The RASSF1C oncogene promotes mesenchymal‐amoeboid transition Top: graphs showing the change in cell diameter for MCF7 cells transfected with ZsGreen (left) or ZsRASSF1C (right) plasmids at 0 h and 12 h, connections indicate individual cell tracking. Bottom: example images diameter measurements (red line). Scale 20 μm.Single‐cell morphology of MCF7 and MDA‐MB‐231 cell lines, expressing or not a RASSF1C construct, cultured in 3D rat tail collagen I and analyzed for their rounded or elongated morphology 24 h after seeding in 3D matrix.Confocal images of MDA‐MB‐231 cells transfected with ZsGreen or ZsRASSF1C grown in 3D collagen matrix. Cells were imaged and stained with Phalloidin‐568 (red), pMLCII/Alexa 633 (false‐colored green), or imaged for ZsGreen (false‐colored white). Scale 20 μm.Single‐cell morphology assay of MDA‐MB‐231 cells cultured in 3D collagen and analyzed for morphology 24 h after transient knock‐down using either a control sequence (NT, non‐targeting) or a sequence targeting the *RASSF1* locus. RASSF1 knock‐down marks a reduction in rounded cells.Gelatine zymography assay shows downregulation of MMP‐9 metalloprotease in MDA‐MB‐231 cells over‐expressing RASSF1C.3D Matrigel Boyden chamber invasion with or without metalloproteases inhibitor GM6001 and 3D Hydrogel invasion of MDA‐MB‐231 control cells or cells over‐expressing RASSF1C (24 h).Quantification of Boyden chamber invasion after 24 h in 3D Matrigel of MDA‐MB‐231 cells, transfected with ZsGreen (control) or ZsRASSF1C (RASSF1C) and treated with inhibitors against ROCK (Y27632, 10 μmol/l) or Rho (C3, 2 μg/ml).Cartoon summarizing intravital imaging experiments.Lung colonization of MDA‐MB‐231 CFP^+^ cells (MDA‐MB‐231^CFP^) stably expressing control or HA‐RASSF1C in mammary gland tumors from 5 mice each, measured from intravital imaging time‐lapse images.Average migration speed (expressed as μm/min) of *n* = 30 randomly selected CFP‐positive cells in intravital images of Control and RASSF1C‐expressing MDA‐MB‐231 tumors.Quantification of CFP^+^ MDA‐MB‐231 cells displaying different types of morphology *in vivo*, expressed as average number of events, observed from a total of 30 positions in *n* = 5 mice/group (Control or RASSF1C). Mesenchymal, amoeboid, and hybrid morphology were scored manually based on diameter and circularity (distance between the two furthest points). The higher distance between cell edges was defined as mesenchymal morphology, while the lower distance was considered amoeboid morphology. Data information: All data are from *n* = 3 independent experiments. Data are analyzed by Student’s *t*‐test and represented as mean ± SEM.*P ≤ 0.05, **P ≤ 0.01, ***P ≤ 0.001. Source data are available online for this figure.

The process of MAT is associated with changes in cell morphology, where actin cytoskeleton and stress fibers are reorganized into a contractile actomyosin cortex. Cancer cells adopting amoeboid mode of motility have then typical rounded morphology in three‐dimensional matrices. To determine whether RASSF1C is involved in MAT, we transiently transfected MCF7 cells with a fluorescently tagged ZsGreen‐RASSF1C (ZsRASSF1C) or control construct (ZsGreen). We observed that ZsRASSF1C‐expressing cells grown on 2D surface displayed a progressive decrease of diameter within 12 h of expression of the construct compared to control (Fig 1A, Appendix Fig S1A, Movie EV1). As rounded phenotype is a morphological change associated with reorganization of actin cytoskeleton and actin cortical localization, this indicated a potential correlation between RASSF1C expression and mesenchymal‐amoeboid transition (Fig 1A). In order to verify whether these phenotypic changes could also be observed in three‐dimensional matrices, the necessary 3D environment for upregulation of Rho/ROCK signaling, we cultured RASSF1C‐expressing cells in 3D extracellular collagen matrices. We observed increased numbers of RASSF1C‐expressing cells that exhibited rounded morphology in 3D collagen matrix in mesenchymal (MDA‐MB‐231) (Fig 1B, bottom) and also in epithelial (MCF7 from 80% of epithelial‐polygonal to 85% rounded morphology and H1299 from 81% epithelial‐polygonal to 88% rounded shape) cell lines (Fig 1B top, Appendix Fig S1B and C), suggesting that RASSF1C may be involved not only in mesenchymal‐amoeboid but also in epithelial‐amoeboid transition (EAT). Next, we visualized the co‐localization of F‐actin (via phalloidin stain) and pMLCII, a marker of high myosin II activity correlated to rounded morphology (Georgouli & Herraiz, 2019), which indicated phenotypic shift from actin stress fibers in the mesenchymal control cells (visible in the phalloidin stain, Fig 1C top panel), to cortical actomyosin in RASSF1C‐expressing MDA‐MB‐231 cells (visible in both pMLCII and phalloidin stain, Fig 1C bottom panel). Therefore, as RASSF1C‐mediated morphological changes are associated with cortically localized actin and myosin, this indicated MAT (Keller & Eggli, 1998; Wyckoff *et al*, 2006). To additionally confirm whether RASSF1C cells also actively adopt rounded phenotype during MAT process, we produced MDA‐MB‐231 spheroids and embedded them into a 3D collagen matrix. We observed that single cells in both control (ZsGreen) and ZsRASSF1C^+ve^ 3D spheroids were able to actively detach and migrate from the spheroids. However, while control cells maintained a typically elongated, fibroblast‐like, shape, cells transfected with RASSF1C showed rounded morphology (Appendix Fig S1D). We reasoned that expression of RASSF1C is responsible for EAT, in MCF7 and H1299 cell lines, and MAT in MDA‐MB‐231 cell line, and for the switch from elongated to rounded morphology induced in 3D collagen matrix. To corroborate these results, we tested whether loss of RASSF1C can revert the phenotype from rounded to elongated, fibroblast‐like shape. To this end, suppression of endogenous RASSF1C expression via siRNA‐mediated knock‐down in MDA‐MB‐231 cells resulted in a significant decrease in the baseline number of rounded cells and a significant increase in the number of elongated cells in 3D matrix (Fig 1D).

As reported in the literature, mesenchymal invasion is dependent on production of metalloproteases that mediate proteolytic degradation of the extracellular matrix (Brooks *et al*, 1996). Conversely, amoeboid invasion does not rely on proteolytic degradation of surrounding tissue (Pandya *et al*, 2017), but is dependent on either squeezing through pre‐existing pores in the extracellular matrix or deforming surrounding tissue by tension generated by cortical actomyosin (Sahai & Marshall, 2003a; Wyckoff *et al*, 2006). To investigate the production of metalloproteases we performed gelatin zymography assay *in situ*, where we analyzed MMP enzymatic activity from the cell medium (Snoek‐van Beurden & Von Den Hoff, 2005). We detected increased activity of MMP‐9 metalloprotease in control MDA‐MB‐231 cells compared to cells expressing RASSF1C (Fig 1E, Appendix Fig S1E for quantification). To confirm this observation, we performed Boyden chamber invasion assay with a broad spectrum metalloprotease inhibitor, GM6001. As expected, in control cells GM6001 inhibited Matrigel invasion (Fig 1F, left bar graph). However, in the presence of RASSF1C, metalloprotease inhibition did not influence the ability of cells to invade Matrigel (Fig 1F, left bar graph). These data suggested that RASSF1C‐mediated mode of invasion was not dependent on metalloproteases. To further investigate this, we performed an invasion assay using hydrogel matrix, which is not degradable by metalloproteases (Fig 1F, right bar graph, and Appendix Fig S1F). Hydrogel invasion assay showed that control mesenchymally invading cells were not able to invade the hydrogel matrix, whereas RASSF1C cell ability to invade the matrix was significantly greater (Fig 1F, right bar graph). These results strongly support the hypothesis that RASSF1C‐mediated mode of invasion is metalloprotease‐independent and we reasoned that it may be supported by traction forces generated via contractile actomyosin, that allow cells to squeeze via pores in a three‐dimensional environment. Given that Rho/ROCK signaling is the major pathway involved in amoeboid motility (Sahai & Marshall, 2003b), we next asked whether the observed RASSF1C‐promoted amoeboid invasion is a consequence of Rho/ROCK upregulation in 3D matrices. Using ROCK (Y27632) and Rho (C3) inhibitors, we could demonstrate that Matrigel invasion by ZsRASSF1C‐expressing MDA‐MB‐231 cells was greatly impaired by both inhibitors, thus demonstrating Rho/ROCK dependency (Fig 1G and Appendix Fig S1G). However, the treatment of control cells with both drugs resulted in no effect on their ability to invade (Fig 1G and Appendix Fig S1G). These data confirmed our previous results, showing that MDA‐MB‐231 control cells use a mesenchymal mode of invasion which is dependent on metalloprotease degradation of the ECM (Fig 1F). To further support Rho/ROCK dependency, we could show that expression of ZsRASSF1C and a dominant‐negative RhoA derivative (RhoA‐DN) suppressed ZsRASSF1C‐driven invasion in both MDA‐MB‐231 and MCF7, while a catalytically active RhoA derivative (RhoA‐CA) enhanced invasion upon RASSF1C expression (Appendix Fig S1H and I).

We next set out to determine whether RASSF1C could promote amoeboid invasion and metastatic spread *in vivo*. To this end, we employed CFP‐labeled MDA‐MB‐231 cells (MDA‐MB‐231^CFP^; Zomer *et al*, 2015) and stably expressed RASSF1C to promote a switch to amoeboid motility (MDA‐MB‐231^CFP;HA‐RASSF1C^). Tumors were initiated in the mammary gland of immuno‐compromised mice, and lung colonization as well as migration of individual cells were tracked by time‐lapse images via intravital microscopy (IVM) (Fig 1H). Importantly, we observed that the number of MDA‐MB‐231^CFP;HA‐RASSF1C^ cells that colonized distal sites in the lungs was significantly greater than the number of control cells, therefore supporting RASSF1C oncogenic potential *in vivo* (Fig 1I). Low‐adhesion attachment during *in vitro* amoeboid invasion in 3D matrices allows amoeboid cells to translocate at relatively high velocities, between 2 and 25 µm/min, while relative speed of mesenchymally invaded cells is approximately 0.1–0.5 µm/min due to recruitment of proteolytic enzymes and slow turnover of focal adhesions (Palecek *et al*, 1997; Friedl *et al*, 1998; Sahai & Marshall, 2003b). We next investigated the speed adopted by RASSF1C cells during *in vivo* invasion. Manual analysis of the invading cell speed in MDA‐MB‐231^CFP;HA‐RASSF1C^ and MDA‐MB‐231^CFP; Control^ tumors showed higher migration speed of cells in MDA‐MB‐231^CFP;HA‐RASSF1C^ tumors (Fig 1J). Interestingly, these cells adopted more amoeboid, rounded morphology during their invasion from MDA‐MB‐231^CFP;HA‐RASSF1C^, compared to control tumors (Fig 1K, morphology was defined as amoeboid, hybrid, or mesenchymal).

The data so far suggest that RASSF1C expression promotes a phenotypic switch from mesenchymal or epithelial to amoeboid morphology and motility both *in vitro* and *in vivo*, likely through upregulation of Rho/ROCK/pMLCII pathway.

### RASSF1C‐mediated SRC activation promotes pro‐amoeboid Rho/ROCKI/pMLCII signaling

**Figure 2:**
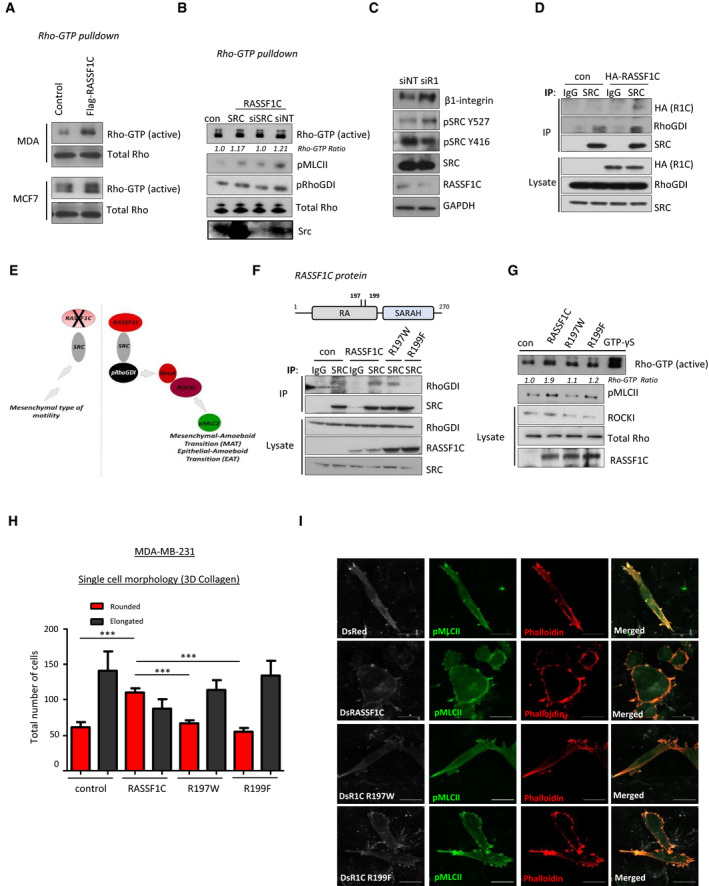
RASSF1C oncogene upregulates Rho/ROCKI/pMLCII signaling pathway via SRC Western blot analysis of Rho‐GTP pull‐down assay in MDA‐MB‐231 and MCF7 cell lines transiently transfected either with control or Flag‐RASSF1C plasmid.Rho‐GTP pull‐down binding assay in MDA‐MB‐231 control or RASSF1C over‐expressing cells and co‐transfected either with SRC plasmids, siRNA against SRC or siNT (non‐targeting sequence). Western blot analysis shows Rho activity (Rho‐GTP‐double band) and pMCLII and pRhoGDI binding. The ratio below Rho‐GTP Western blot shows differences in Rho‐GTP protein expression, compared to control condition.Western blot analysis of proteins in MDA‐MB‐231 cells transiently transfected with siRNA targeting a control sequence (NT) or targeting RASSF1. GAPDH was used as a loading control.Immunoprecipitation from MDA‐MB‐231 transiently transfected with control (pcDNA3) or HA‐RASSF1C plasmid. Pull‐down was performed using a SRC or same species IgG antibody and blotted with indicated antibodies.Schematic cartoon recapitulating the proposed RASSF1C/SRC‐driven mechanism.Top, schematic of RASSF1C domain structure indicating putative mutations in the RA domain affecting RhoA activity. Bottom, immunoprecipitation of MDA‐MB‐231 transiently transfected with either control (DsRed), DsRASSF1C plasmid, DsRASSF1C‐R197W or DsRASSF1C‐R199F mutants. Pull‐down was performed using a SRC or same species IgG antibody and blotted with indicated antibodies.Western blot analysis of Rho‐GTP pull‐down assay in MDA‐MB‐231 cells transiently transfected with RASSF1C or its mutants (R197W, R199F). The ratio under Rho‐GTP Western blot shows differences in active Rho protein expression, compared to control condition.Quantification of rounded versus elongated cells in single‐cell morphology assay in 3D collagen indicating the degree of mesenchymal‐amoeboid transition of MDA‐MB‐231 cells when RASSF1C or the described mutants are expressed.Representative confocal images show elongated or rounded cells per field of view of MDA‐MB‐231 cells transfected with DsRed, DsRASSF1C, DsRASSF1C‐R197W or DsRASSF1C‐R199F, grown in 3D collagen and stained with Phalloidin‐568 (red) for F‐actin and pMLCII/Alexa 633. Scale bars represent 10 μm. Data information: For both Rho‐GTP pull‐downs and immunoprecipitation assays total proteins were used as loading controls. All data are from *n* = 3 independent experiments. Data are analyzed by Student’s *t*‐test, represented as mean ± SEM. ***P ≤ 0.001. Source data are available online for this figure.

**Figure EV2:**
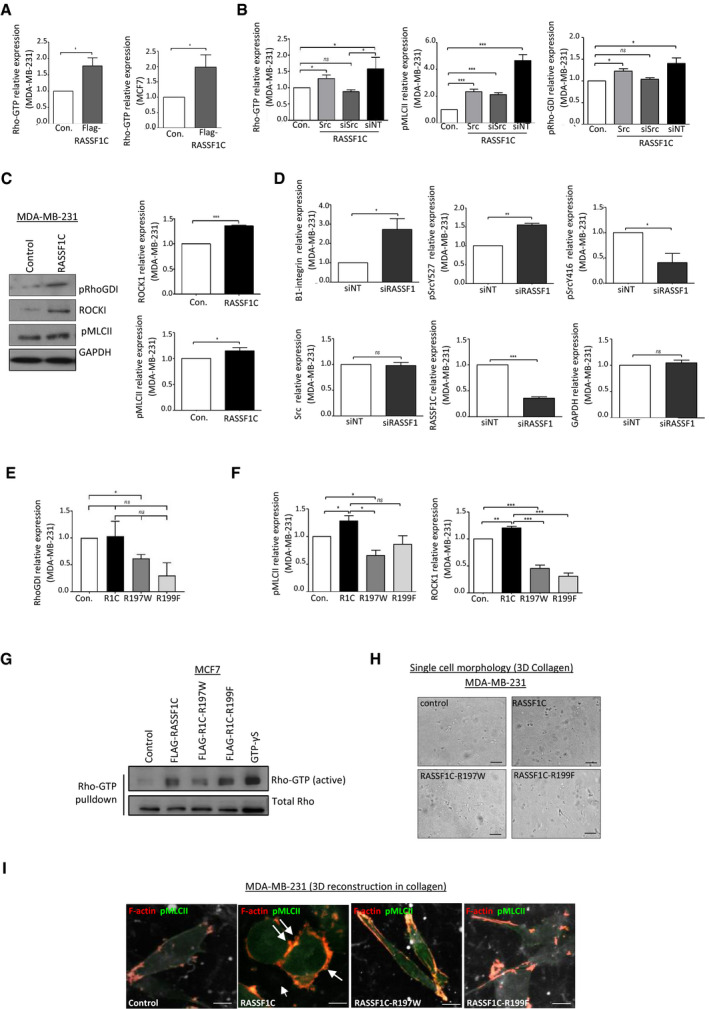
RASSF1C promotes Rho/ROCKI signaling and amoeboid morphology Quantification of Western blot analysis, related to Fig 2A.Quantification of Western blot analysis, related to Fig 2B.Western blot analysis and quantification of protein expression in MDA‐MB‐231 cells transiently transfected with an empty vector or a RASSF1C construct. GAPDH was used as a loading control.Quantification of Western blot analysis, related to Fig 2C.Quantification of Western blot analysis, related to Fig 2F.Quantification of Western blot analysis, related to Fig 2G.Active Rho‐GTP pull‐down assay from MCF7 cells transfected with Control, FLAG‐RASSF1C, FLAG‐RASSF1C‐R197W, or FLAG‐RASSF1C‐R199F plasmids.Related to Fig 2H, representative images, from which quantification in Fig 2H was derived, of single‐cell morphology assay in 3D collagen indicating mesenchymal‐amoeboid transition of MDA‐MB‐231 cells when RASSF1C or mutant derivatives are expressed, Scale bar 20 µm.Maximum Intensity Projection of confocal images of MDA‐MB‐231 cells grown in 3D collagen and transfected with ZsGreen, ZsRASSF1C wild‐type or mutant plasmids. Cells were stained with phalloidin for F‐actin (red) and pMLCII (far red, false‐colored to green). Arrows indicate site of cell blebbing. Scale 5 μm. Data information: Data are analyzed by Student’s *t*‐test and represented as mean ± SEM.*P ≤ 0.05, **P ≤ 0.01, ***P ≤ 0.001, *n* = 3. Source data are available online for this figure.

We next set out to explore the mechanism through which RASSF1C promotes MAT. RASSF1C directly supports activation of SRC kinase by preventing CSK inhibitory phosphorylation on Y527 (Vlahov *et al*, 2015). Notably, SRC activation is well‐documented to promote mesenchymal motility; however, it can also facilitate contractility via inhibitory phosphorylation of RhoGDI (pY156), which results in activation of RhoA (DerMardirossian *et al*, 2006). Therefore, we hypothesized that RASSF1C activation of SRC may be implicated in MAT via upregulation of Rho/ROCK/pMLCII signaling pathway. As the Rho interacting domain of RASSF1A is identical to RASSF1C (Lee *et al*, 2016), we investigated whether RASSF1C promotes Rho activation. Rho‐GTP pull‐down assay on lysates obtained from cells expressing a control or a RASSF1C construct showed that RASSF1C expression results in accumulation of active Rho (shown as Rho‐GTP) in MDA‐MB‐231 and MCF7 cells (Figs 2A and EV2A for quantification). Next, we performed Rho‐GTP pull‐down in cells expressing RASSF1C and co‐expressing a SRC construct or depleted of SRC by siRNA knock‐down (Figs 2B and EV2B for quantification). Transient knock‐down of SRC by siRNA showed a decrease in Rho activity (measured as lower expression levels of Rho‐GTP in the pull‐down). Interestingly, SRC over‐expression resulted in increased inhibitory phosphorylation of RhoGDI (pY156), a Rho‐GTPase inhibitor, and elevated levels of pMLCII (pS19) (Figs 2B and EV2B). Similarly, RASSF1C over‐expression alone could induce RhoGDI phosphorylation, increased expression of ROCKI and pMLCII (Fig EV2C). Furthermore, inhibition of endogenous RASSF1C expression by siRNA in MDA‐MB‐231 cells resulted in reduced SRC activating phosphorylation (pY416), increased SRC inhibitory phosphorylation (pY527), concomitant with increased expression of β1‐integrin, a crucial regulator of stable attachment to ECM in mesenchymally invaded cells (Pandya *et al*, 2017) (Figs 2C and EV2D for quantification). The above results led us to hypothesize that the morphological changes observed in RASSF1C cells (Fig 1) were due to RASSF1C upregulation of Rho/ROCK/pMLCII signaling axis via activation of SRC. SRC in turn inhibits RhoGDI, event that allows activation of Rho. To confirm that SRC and RhoGDI are downstream of RASSF1C in Rho/ROCK upregulation, we performed co‐immunoprecipitation by pulling down endogenous SRC and determined that both RASSF1C and RhoGDI are found in complex with SRC (Fig 2D for SRC and IgG control IPs, Fig 2E, cartoon that recapitulates the hypothesized mechanism). A previous study described that the main isoform of the *RASSF1* gene, the tumor suppressor RASSF1A, is able to repress RhoA activity and MLCII phosphorylation through its RA (Ras association) domain (Lee *et al*, 2016). Based on this report, we next generated two point mutations in the identical RA domain sequence of RASSF1C, RASSF1C‐R197W, and RASSF1C‐R199F, which had previously been demonstrated to be necessary for RASSF1A‐mediated inhibition of RhoA (Lee *et al*, 2016). Compared to RASSF1C, RASSF1C‐R199F and RASSF1C‐R197W mutants were less capable of stimulating SRC binding to RhoGDI or activate Rho, which in turn led to decreased MLCII phosphorylation and ROCKI expression (Fig 2F for IP of endogenous SRC or IgG control, Figs 2G and EV2E–G). Moreover, we confirmed that RASSF1C‐R199F and RASSF1C‐R197W mutants failed to promote rounded morphology, and instead exhibited elongated, fibroblast‐like morphology in 3D collagen matrix, typical of mesenchymal cells (Fig 2H for quantification and EV2H for representative images). This mesenchymal phenotype was accompanied by formation of abundant stress fibers with no signs of formation of cortical actomyosin (Fig 2I). Tension and contractility of the actomyosin network initiated by Rho/ROCK signaling in amoeboid cells promote rupture of the actomyosin cortex resulting membrane blebs, spherical membrane herniations (Keller & Eggli, 1998). These membrane blebs were typical only in RASSF1C amoeboid cells, while RASSF1C‐R199F and RASSF1C‐R197W mutants maintained their mesenchymal, elongated phenotype in 3D substrates (Fig EV2I). Here we show for the first time that pro‐mesenchymal activated SRC can also be involved in amoeboid invasiveness via inhibition of RhoGDI, a RhoA antagonist, which in turn allows activation of Rho/ROCK/pMLCII signaling and cytoskeleton reorganization.

### RASSF1C amoeboid cells release pro‐invasive extracellular vesicles

**Figure 3:**
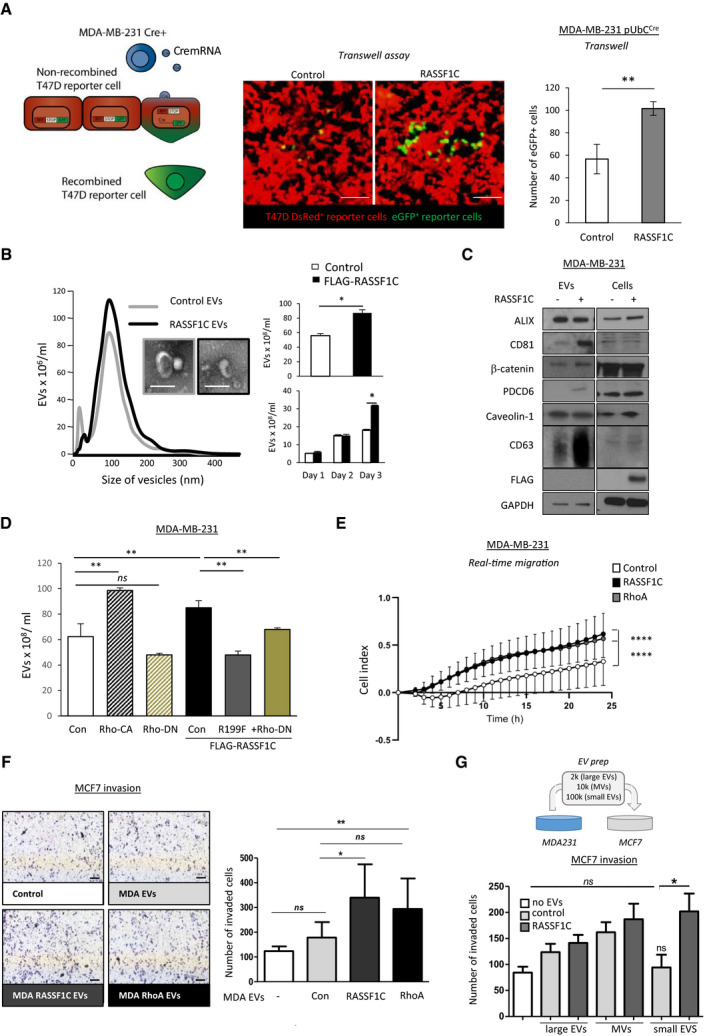
Amoeboid RASSF1C cells produce pro‐invasive Extracellular Vesicles Left: Cartoon showing the principle behind the Cre‐carrying EVs and how recipient cells switch from DsRed^+^ to eGFP^+^ expression upon Cre‐mediated excision of the DsRed locus. Center: representative images, and right: quantification of T47D^DsRed^ cells that converted to eGFP^+^ in the bottom wells of a transwell system in the presence of MDA‐MB‐231^CFP;Cre;Control^ or MDA‐MB‐231^CFP;Cre;HA‐RASSF1C^ seeded in the upper wells. Scale bars represent 100 μm.Left: averaged Nanoparticle Tracking Analysis displaying the size distribution of EVs isolated from media of MDA‐MB‐231 cells expressing Control (pcDNA3) or FLAG‐RASSF1C plasmid. Inserts: transmission electron microscopy images of single EVs. Scale bars represent 200 nm. Right: quantification of total secreted EVs (96 h, top) or at indicated times (below), as measured by NTA.Western blot analysis of EVs isolated from MDA‐MB‐231 cells transfected with Control (pcDNA3) or FLAG‐RASSF1C with indicated antibodies. Equal amounts of protein were loaded after normalization via microBCA assay.Quantification of EVs isolated from MDA‐MB‐231 cells transfected with Control (pcDNA3), RhoA‐CA, RhoA‐DN, FLAG‐RASSF1C, FLAG‐RASSF1C‐R199F, or FLAG‐RASSF1C + RhoA‐DN using Nanoparticle Tracking Analysis.Normalized values of real‐time migration rates in xCELLigence plates of MDA‐MB‐231 cells treated with 20 ng/μl EVs derived from MCF7 cells expressing a Control, FLAG‐RASSF1C, or EGFP‐RhoA plasmid, left to migrate for 24 h. Statistics were performed with 2‐way ANOVA (Dunnett’s multiple comparisons test).Left: Representative images of Boyden chamber invasion assay in 3D Matrigel of MCF7 cells, treated with 20 ng/μl EVs derived from MDA‐MB‐231 cells, transfected the same constructs used in (E). Right: Quantification of Boyden chamber invasion 24 h after EV treatment. Scale bars represent 200 µm.Top: graphical representation of the functional (invasion) assay performed using MDA‐MB‐231 derived EVs on MCF7 recipient cells. Bottom: Boyden chamber invasion assay in 3D Matrigel of EVs isolated from different steps of ultracentrifugation. EVs were collected from conditioned medium produced by MDA‐MB‐231 expressing Control (pcDNA3) or FLAG‐RASSF1C. The 2K (large EVs), 10K (medium EVs or MVs), and 100K (small EVs, including exosomes) pellets were collected and equal amount of EVs (normalized according to protein content) were incubated with MCF7 cells. MCF7 were evaluated for invasion after 24 h. Data information: All data are from *n* = 3 independent experiments. Data are analyzed by Student’s *t*‐test and represented as mean ± SEM.*P ≤ 0.05, **P ≤ 0.01, ****P ≤ 0.0001. Source data are available online for this figure.

**Figure EV3:**
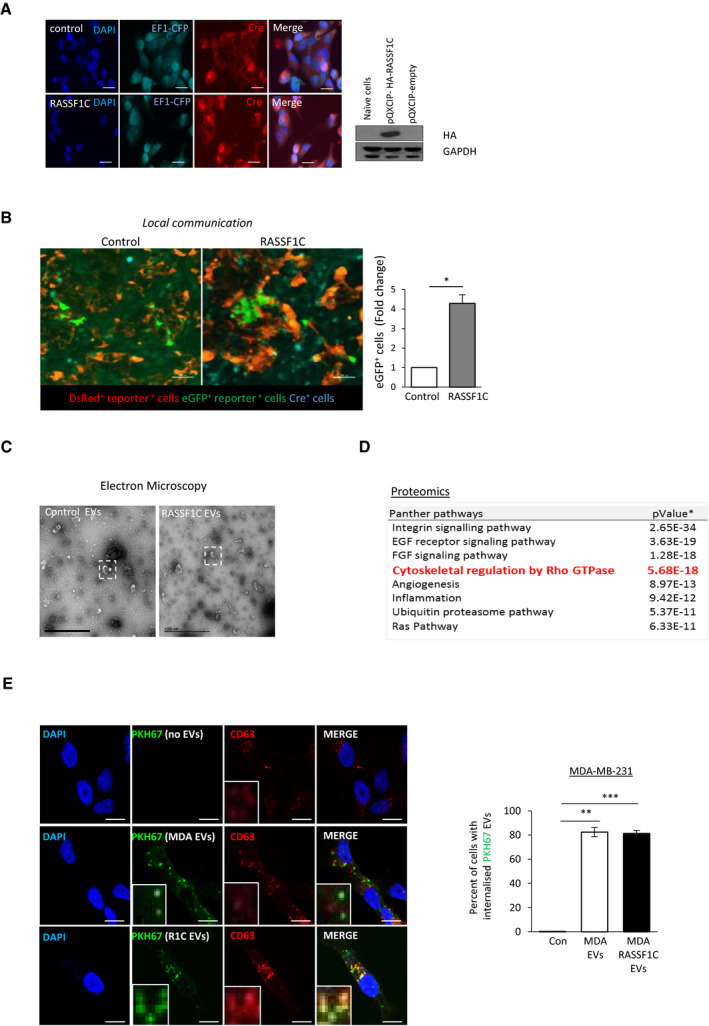
Upregulation of RASSF1C promotes EV release Left: Confocal images of MDA‐MB‐231^CFP;Cre;Control^ or MDA‐MB‐231^CFP;Cre;HA‐RASSF1C^ stained for Cre expression (red). Scale bars 20 μm. Right: Western blots of RASSF1C expression (probed as HA tag) in MDA‐MB‐231^CFP;Cre;Control^ and MDA‐MB‐231^CFP;Cre;HA‐RASSF1C^. GAPDH was used as loading control.Images and quantification of T47D cells that express eGFP^+^ in a mixed culture of T47D reporter cells and MDA‐MB‐231 donor cells infected with pQXCIP (MDA‐MB‐231^CFP;Cre;Control^) or pQXCIP‐HA‐RASSF1C (MDA‐MB‐231^CFP;Cre;HA‐RASSF1C^). Scale bars 100 μm.Full view transmission electron microscopy images of EVs isolated from MDA‐MB‐231 cells expressing Control (pcDNA3) or Flag‐RASSF1C, from which magnifications in Fig 3B are taken. White dashed boxes show single extracellular vesicles magnified in Fig 3B. Scale bars 1,000 nm.Panther pathway analysis of the shared EV proteome of MDA‐MB‐231 cells transfected with Control (pcDNA3) or FLAG‐RASSF1C. The presence of Rho‐GTPase‐related pathways in EVs is highlighted in red. P values were calculated with a two‐tailed Student’s *t*‐test.Representative pictures and quantification of the percentage of MDA‐MB‐231 cells that have internalized PKH67 stained EVs isolated from MDA‐MB‐231 expressing Control (MDA EVs) or FLAG‐RASSF1C (R1C EVs). Recipient cells have been fixed, stained, and imaged 18 h after treatment with 50 ng/μl EVs. Scale bars represent 20 μm. Inset images show magnification of internalized EVs. Data information: Data are analyzed by Student’s *t*‐test and represented as mean ± SEM.*P ≤ 0.05, **P ≤ 0.01, ***P ≤ 0.001, *n* = 3. Source data are available online for this figure.

Having shown that RASSF1C expression leads to MAT and invasion via modulation of Rho/ROCK/pMLCII signaling pathway, we hypothesized that RASSF1C could promote invasion via additional mechanisms. Growing evidence in the literature describes the importance of extracellular cues, such as extracellular vesicles (EVs), involved in intercellular communication and in mediating cancer invasiveness (Quail & Joyce, 2013a). To study cell‐cell communication via EV transfer, we employed a previously reported system comprised of a donor and a reporter cell line. The donor cell line, i.e., MDA‐MB‐231^CFP, UbC‐Cre^, expressing Cre under the ubiquitin promoter, has been shown to package Cre mRNA into EVs (Zomer *et al*, 2015). The reporter cell line, i.e., T47D luminal breast cancer cells, harbors both a floxed *DsRed* and a lox‐stop‐lox *eGFP* allele. When the two cell types are co‐cultured, T47D reporter cells that internalize EVs containing Cre mRNA switch from DsRed to eGFP, thereby allowing distinction between Cre^+^ EV recipients (green) from unrecombined T47D cells (red, Fig 3A, left). We tested the system *in vitro* by generating donor MDA‐MB‐231^CFP, UbC‐Cre^ cells stably expressing a control or a RASSF1C construct (Fig EV3A). We performed experiments with reporter T47D cells where donor/reporter cells were either in direct contact (co‐culture) or separated by a 0.4 μm porous membrane (transwell). Both the co‐culture (Fig EV3B) and the transwell setup (Fig 3A) showed a significant increase in eGFP events, thereby indicating that RASSF1C modulates cell communication, likely through transfer of EVs.

In line with these results, we therefore sought to understand whether amoeboid RASSF1C cells shed EVs able to transfer the invasive potential to recipient cells. We isolated EVs, via a differential centrifugation protocol, from MDA‐MB‐231 cells expressing either a control or Flag‐RASSF1C construct, cultured in medium with 0.1% EV‐depleted FBS. Employing Nanoparticle Tracking Analysis (NTA), we observed a significantly higher number of EVs released by RASSF1C cells (Fig 3B), which could explain the differences observed in recombined reporter cell numbers in the Cre‐LoxP model (Fig 3A). The mean EV size (˜100 nm) indicated isolation of small EVs and it was confirmed by Electron Microscopy (Fig 3B insets and EV3C for complete field of view). In order to rule out the possibility that exogenous plasmid expression alone could drive EV release, we cultured cells for up to 72 h and isolated EVs at different time‐points (24, 48, and 72 h, Fig 3B, bottom bar graph). Collection of the conditioned media at defined time‐points suggested that the accumulation of EVs occurred over days following transfection with RASSF1C, suggesting that this occurs in response to changes in cell behavior and is not an acute release in response to RASSF1C expression. We next set out to characterize released EVs by protein composition. Western blotting of isolated EVs showed selective enrichment of some commonly used EV markers, e.g. CD63 and CD81 vs ALIX, in RASSF1C samples (Fig 3C). Interestingly, RASSF1C protein enrichment on EVs was not observed (Fig 3C, Flag tag). In order to obtain a more comprehensive picture of the proteome present in RASSF1C‐derived EVs, we performed Mass Spectrometry and found that the general proteome of MDA‐MB‐231 EVs is dominated by proteins involved in integrin signaling and cytoskeletal regulation by Rho signaling (PANTHER pathway enrichment, Fig EV3D), but not greatly altered by RASSF1C. This observation suggests that any functional effect of RASSF1C‐derived EVs may be ascribed to a different type of EV cargo or to a specific EV subpopulation. Having shown that RASSF1C expression leads to amoeboid transition in MDA‐MB‐231 cells, we next sought to understand if Rho signaling was also involved in RASSF1C‐mediated release of EVs. Previously used RhoA and RASSF1C constructs (Appendix Fig S1H, Fig 2F–H) were expressed in MDA‐MB‐231 cells and EVs were isolated as described before. NTA measurements showed that over‐expression of a constitutively active derivative of RhoA (RhoA‐CA) enhanced EV release similarly to RASSF1C expression, whereas a RhoA dominant‐negative derivative (RhoA‐DN) had an opposite effect (Fig 3D). Moreover, expression of a RASSF1C derivative with impaired capacity of activating Rho signaling (RASSF1C‐R199F) or co‐expression of RASSF1C wild‐type and RhoA‐DN also hinders EV release, suggesting that the effects on EVs driven by RASSF1C may involve RhoA activation.

Having shown that RASSF1C cells promote EV transfer, we next asked whether EVs can confer a selective migratory and invasive advantage to recipient cells. In order to answer this question, we first performed an uptake experiment to ensure that isolated EVs are functional and can be further used for functional assays. EVs were isolated from MDA‐MB‐231 as previously described, labeled with PKH67 lipophilic dye, and equal amounts of EVs (normalized for protein content by microBCA) were incubated with recipient MDA‐MB‐231 cells. As shown in Fig EV3E, both control and RASSF1C EVs were internalized by recipient cells at similar rates.

We next performed functional assays by incubating recipient (MDA‐MB‐231 or MCF7) cells with EVs isolated from donor MDA‐MB‐231 cells expressing a control, a RASSF1C, or a RhoA construct.

Incubation of cells with RASSF1C or RhoA EVs (20 ng/μl dose) stimulated significantly higher cell migration than incubation with control EVs (Fig 3E, xCELLigence transwell migration assay, displayed results are normalized to baseline untreated cells). At higher doses (40 ng/μl, Appendix Fig S2A), EVs derived from control cells were able to promote recipient cell migration at similar rates to EVs derived from RASSF1C or RhoA.

We next incubated the same panel of EVs with recipient MCF7 cells seeded on a Matrigel layer, in order to measure Matrigel invasion. Strikingly, the number of invaded cells was significantly higher when cells were treated with 20 ng/μl of RASSF1C or RhoA EVs, compared to control EVs and untreated cells (Fig 3F, Appendix Fig S2B for a comparison of both concentrations).

Cells have been shown to release heterogeneous populations of EVs (Willms *et al*, 2016), and classification of the different subgroups is still subject to ongoing research (Willms *et al*, 2018). In order to investigate whether multiple EV populations could transfer the observed oncogenic phenotype to recipient cells, we isolated fractions using three centrifugation steps: 2,000 *g* (large EVs), 10,000 *g* (MV or medium EVs; Sedgwick *et al*, 2015), and 100,000 *g* (small EVs, among which exosomes exist, corresponding to the protocol used throughout the study), and incubated recipient cells with control or RASSF1C EV populations (Fig 3G). Figure 3G shows that only RASSF1C small EVs induced a significantly higher number of cells to invade Matrigel in a transwell assay, if compared to control small EVs. In our setup, RASSF1C large and medium EVs did not confer significantly higher invasive ability to recipient cells, compared to the corresponding control fractions.

To further establish whether the small EVs population is mediating the invasive phenotype observed in the small EV fraction recovered from ultracentrifugation (Fig 3G), we employed a Tangential Flow Filtration/Size Exclusion Chromatography isolation protocol and incubated recipient cells with RASSF1C or control small EVs (Appendix Fig S2C). First, EVs isolated with this method were characterized by Western blotting and NTA. EVs displayed protein markers already detected with the ultracentrifugation protocol (Appendix Fig S2C, left) and NTA showed similar particle number between RASSF1C and control EVs, with this isolation method (Appendix Fig S2C, middle). Importantly, RASSF1C small EVs still induced a significantly higher number of recipient cells to invade the Matrigel membrane, compared to control small EVs (Appendix Fig S2C, right). The data so far suggest that, despite all EV fractions showing a degree of ability to confer invasive potential, it is the RASSF1C‐derived small EVs that significantly transferred invasive potential to naïve cells.

It is important to note that, throughout our study, the treatment of recipient cells with MDA‐MB‐231 derived control EVs (expressing a control vector) already induced an increase in cell migration or invasion, compared to untreated cells (Fig 3E–G, Appendix Fig S2C). This is expected, as it is well‐documented that EVs from highly invasive cancer cells can promote migration and invasion in recipient cells. However, oncogenic activity of RASSF1C consistently led to a significant increase of invasive potential in recipient cells.

Taken together, the data show that RASSF1C promotes EV functional transfer and the release of pro‐invasive EVs, and that both RASSF1C and RhoA expression promote the release of pro‐migratory EVs. Our data suggest that RASSF1C oncogenic potential has an effect on EV secretion and this may occur through Rho activation.

### RASSF1C amoeboid cells display cancer stemness features transferrable via EVs

**Figure 4:**
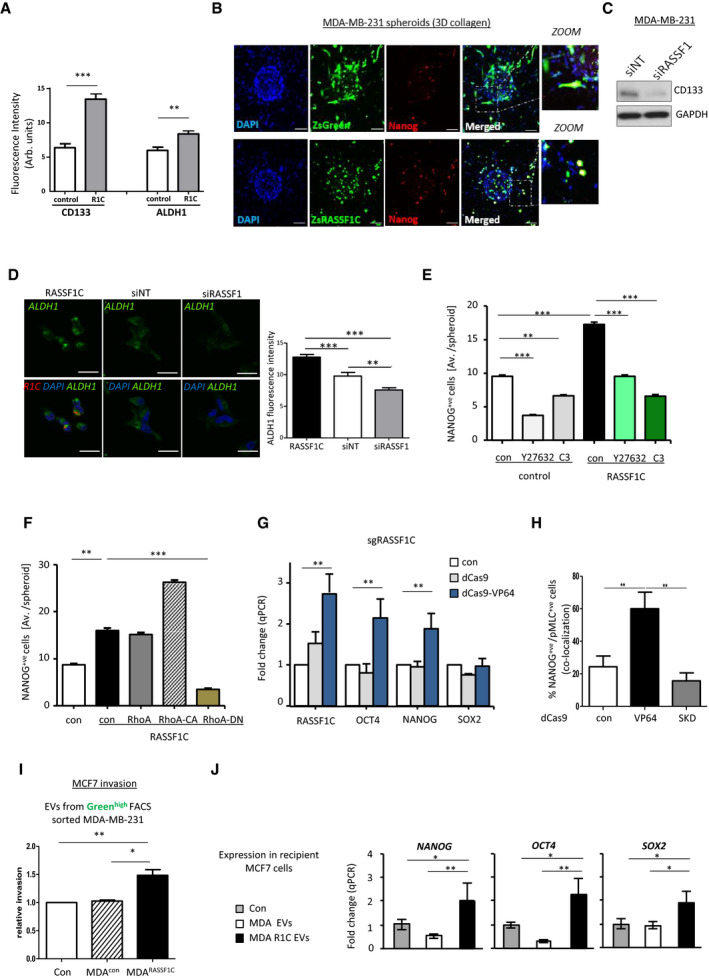
Amoeboid RASSF1C cells express cancer stemness features Quantification of fluorescent intensity of CD133 and ALDH1 cancer stem cell markers in MDA‐MB‐231 control or RASSF1C transfected spheroids cultured on Matrigel matrix.Confocal images of ZsGreen or ZsRASSF1C‐expressing MDA‐MB‐231 spheroids embedded in 3D rat tail collagen I. Cells were stained with NANOG (red). Zoom images show that RASSF1C‐expressing cells are also NANOG‐positive. Scale 100 μm.Western blot analysis of CD133 protein expression level in MDA‐MB‐231 cells treated with siNT or siRASSF1.Representative confocal images (upper row) and quantification of fluorescent intensity of ALDH1 expression in MDA‐MB‐231 cells expressing RASSF1C (black bar in the graph) or after siRNA treatment against RASSF1 (gray bar). Lower row shows merged images with DAPI (blue), ALDH1 (green), and RASSF1C (red). Scale bars represent 20 μm.Quantification of the absolute number of NANOG‐positive cells (measured as average cell number/spheroid) per MDA‐MB‐231 ZsGreen (control) or ZsRASSF1C (RASSF1C) spheroids in 3D collagen, treated with ROCK inhibitor (Y27632, 10 μmol/l) or Rho inhibitor (C3, 2 μg/ml).Quantification of the absolute number of NANOG‐positive cells (measured as average cell number/spheroid) per MDA‐MB‐231 ZsGreen (control, con) or ZsRASSF1C (RASSF1C) spheroids in 3D collagen, co‐transfected with either RhoA WT, catalytically active (CA) or dominant‐negative (DN) constructs.Fold change in qRT–PCR of NANOG, OCT4, and SOX2 mRNA from MCF7 cells expressing CRISPR‐dCas9 or dCas9‐VP64 plus single guide RNA for the RASSF1C promoter (sgRASSF1C).Quantification of the percentage of double‐positive pMLCII and NANOG cells in MCF7 cells expressing CRISPR‐dCas9, dCas9‐VP64, dCas9‐SKD plus single guide RNA for the RASSF1C promoter (sgRASSF1C). At least 300 cells were counted per experiment.Quantification of number of invaded MCF7 cells in 3D Matrigel, after incubation with EVs from MDA‐MB‐231 cells sorted for Green^high^ expression. MDA‐MB‐231 cells stably expressing a pQXCIP‐zsGreen or pQXCIP‐zsGreen‐RASSF1C construct were sorted and EVs were harvested 24 h later, prior to seeding with MCF7 cells in Boyden chambers. Invasive potential of MCF7 cells was evaluated 24 h after incubation with EVs.qRT–PCR expression of NANOG, OCT4, SOX2 in MCF7 cells 24 h after exposure to 50 ng/μl EVs derived from MDA‐MB‐231 cells (treated as indicated). Data Information: All data are from *n* = 3 independent experiments. Data are analyzed by Student’s *t*‐test and represented as mean ± SEM.*P ≤ 0.05, **P ≤ 0.01, ***P ≤ 0.001. Source data are available online for this figure.

**Figure EV4:**
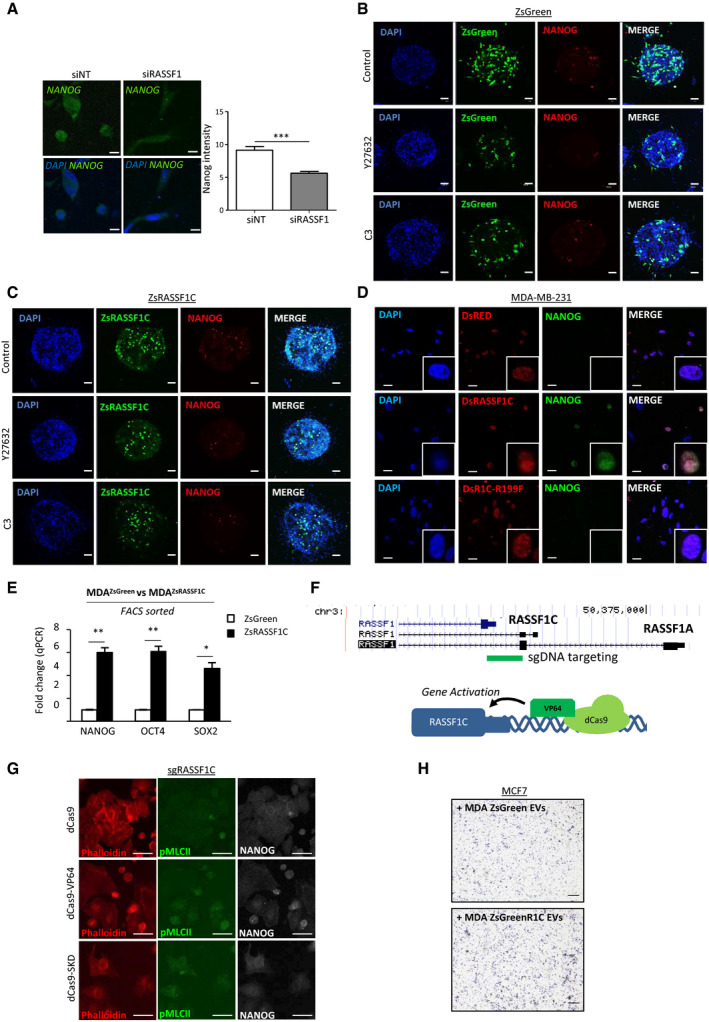
RASSF1C is associated with cancer stemness Representative confocal images and quantification of fluorescent intensity of NANOG expression in MDA‐MB‐231 cells after siRNA treatment against RASSF1 (siRASSF1). Scale bars represent 10 μm.Confocal images of MDA‐MB‐231 ZsGreen spheroids treated with ROCK inhibitor (Y27632, 10 μmol/l) or Rho inhibitor (C3, 2 μg/ml). Cells were stained for NANOG (red). Scale bars represent 200 μm.Confocal images of MDA‐MB‐231 ZsRASSF1C spheroids treated with ROCK inhibitor (Y27632, 10 μmol/l) or Rho inhibitor (C3, 2 μg/ml). Cells were stained for NANOG (red). Scale bars represent 200 μm.Confocal images of MDA‐MB‐231 cells grown on 3D collagen and transfected with Control (DsRed), DsRASSF1C or DsRASSF1C‐R199F (DsR1C‐R199F). Cells were stained with NANOG (green). White boxes show detailed images of single nuclei. Scale bars represent 20 μm.Fold change of qRT–PCR of NANOG, OCT4, and SOX2 mRNA from FACS sorted MDA‐MB‐231 ZsGreen or ZsRASSF1C.Cartoon representing the methodology used to induce the expression of RASSF1C using the CRISPR‐dCas9 system and genomic location of guide RNA target location.Related to Fig 4H, representative confocal images of MCF7 cells expressing CRISPR‐dCas9, dCas9‐VP64, dCas9‐SKD plus single guide RNA for the RASSF1C promoter (sgRASSF1C) and stained for Phalloidin (red), pMLCII (green), and NANOG (white). Scale bars 20 μm.Related to Fig 4I, representative images of Boyden chamber invasion of MCF7 cells incubated with EVs derived from MDA‐MB‐231 ZsGreen (control) or RASSF1C (ZsGreenR1C) cells sorted for Green^high^ expression. Scale bar 200 µm. Data information: Data are analyzed by Student’s *t*‐test and represented as mean ± SEM.*P ≤ 0.05, **P ≤ 0.01, ***P ≤ 0.001, *n* = 3.

Amoeboid transition has been previously linked to cancer stemness in melanoma cells, and RhoA has been directly associated with the expression of stem‐like features (Taddei *et al*, 2014; Pietrovito *et al*, 2018). Given the oncogenic role of RASSF1C and its ability to activate Rho (Fig 2), we next assessed whether RASSF1C‐promoted MAT could also be associated with cancer stemness during *in vitro* invasion. A plethora of markers have been elucidated and used to validate a cancer stemness profile. Among those, the surface glycoprotein CD133 and the aldehyde dehydrogenase enzyme ALDH1 are currently used as breast cancer stemness markers (Ginestier *et al*, 2007). We therefore assessed the expression of ALDH1 and CD133 in RASSF1C 3D spheroids cultivated on Matrigel matrix to mimic colony formation assay, an assay widely used to investigate stemness phenotype *in vitro*. We observed significantly higher expression of these markers in RASSF1C spheroid colonies, compared to control 3D spheroids (Fig 4A). NANOG, SOX2, and OCT4 (octamer‐binding transcription factor 4) are key regulators of pluripotency and tumor invasion, and they have been proposed as biomarkers for cancer stemness in breast and renal carcinoma (Ponti *et al*, 2005; Rasti *et al*, 2018). MDA‐MB‐231 spheroids expressing either a ZsGreen or ZsGreen‐RASSF1C construct were embedded in 3D collagen matrix, and cells were allowed to invade from the aggregates and stained for the stemness marker NANOG. A *Z‐stack* image in Fig 4B shows that invaded amoeboid cells from ZsGreen‐RASSF1C spheroids retained both rounded morphology (see magnification in Fig 4B) and also higher NANOG expression compared to control. In contrast, migrating cells from control 3D spheroids exhibit reduced NANOG fluorescence intensity, suggesting that mesenchymal invasion is not associated with a stemness phenotype. Furthermore, transient knock‐down of RASSF1C decreased CD133 protein levels (Fig 4C), as well as fluorescent intensity of ALDH1 and NANOG in MDA‐MB‐231 cells (Figs 4D and EV4A).

To verify that cancer stemness is dependent on amoeboid Rho/ROCK signaling, we treated control and RASSF1C spheroids in 3D collagen matrix with either a RhoA (C3) or a ROCK inhibitor (Y27632). Both inhibitors significantly decreased the number of NANOG^+^ cells in ZsGreen‐RASSF1C‐expressing spheroids (Figs 4E and EV4B and C). Similarly, co‐expression of RASSF1C and RhoA‐CA constructs greatly increased NANOG expression in MDA‐MB‐231 3D spheroids; however, this effect was reverted when RhoA‐DN was co‐expressed (Fig 4F), suggesting Rho/ROCK dependency. Furthermore, RASSF1C‐R199F mutant cells cultured in 3D collagen matrix also showed impaired NANOG expression, due to inability of Rho activation by this mutant (Fig EV4D). In order to ensure that the expression of stem‐like features was originating from the RASSF1C‐expressing pool of cells, we sorted for green^high^ expression MDA‐MB‐231 stably expressing ZsGreen or ZsGreen‐RASSF1C and analyzed mRNA levels of pluripotency genes such as NANOG, OCT4, and SOX2. All three markers were significantly upregulated in sorted ZsGreen‐RASSF1C cells (Fig EV4E). In order to validate RASSF1C as a modulator of cancer stemness with an alternative approach, we induced endogenous *RASSF1C* expression by epigenetic editing; where inactive Cas9 derivatives (dCas9) fused to a transcriptional activation domain (dCas9‐VP64) and single guide RNAs target the internal *RASSF1* promoter (sgRASSF1C) to selectively promote expression of the RASSF1C transcript (see Fig EV4F, graphical abstract). MCF7 cells expressing a dCas9‐VP64 construct showed upregulation of NANOG and OCT4, along with the RASSF1C mRNA transcript (Fig 4G). Interestingly, in this assay, no upregulation of the pluripotency marker SOX2 was observed. Next, we aimed to show that RASSF1C amoeboid cells and cancer stem cells are the same entities. To this end, we employed the Cas9 technique and visualized both pMLCII (an amoeboid cell marker) and NANOG (a cancer stem cell marker). Amoeboid cells with increased endogenous RASSF1C expression, induced by dCas9‐VP64, have elevated co‐expression of both contractile actomyosin and NANOG, compared to cells transfected with a repressive Cas9 derivative, dCas9‐SKD, where the pMLCII/NANOG^+ve^ pool was significantly reduced (Figs 4H and EV4G). The presented data suggest that RASSF1C expression and its downstream Rho/ROCK/pMLCII pathway upregulation re‐wire cells to express cancer stemness markers such as CD133, ALDH1, NANOG, and OCT4. Having previously shown that RASSF1C/RhoA drive invasive behavior through the release of EVs (Fig 3), we next wanted to assess whether the cancer stem cell pool of RASSF1C cells promotes invasive behavior via EVs. In order to maximize the RASSF1C^+^ cell pool, we sorted ZsGreen‐Control and ZsGreen‐RASSF1C‐expressing MDA‐MB‐231 cells for green^high^ expression by FACS and isolated EVs from the sorted pools. Equal amounts of EVs from control and RASSF1C^+^ cells were then incubated with recipient low invading MCF7 cells, and cells were allowed to invade through Matrigel. The number of invaded MCF7 cells was significantly higher after incubation with Zsgreen^high^ RASSF1C EVs compared to control EVs (Figs 4I and EV4H). Furthermore, RASSF1C‐derived EVs also displayed the ability to promote upregulation of stem cell markers in recipient MCF7 cells, as shown in Fig 4J.

### RASSF1C amoeboid cells induce invasion and metastasis via EV transfer *in vivo*

**Figure 5:**
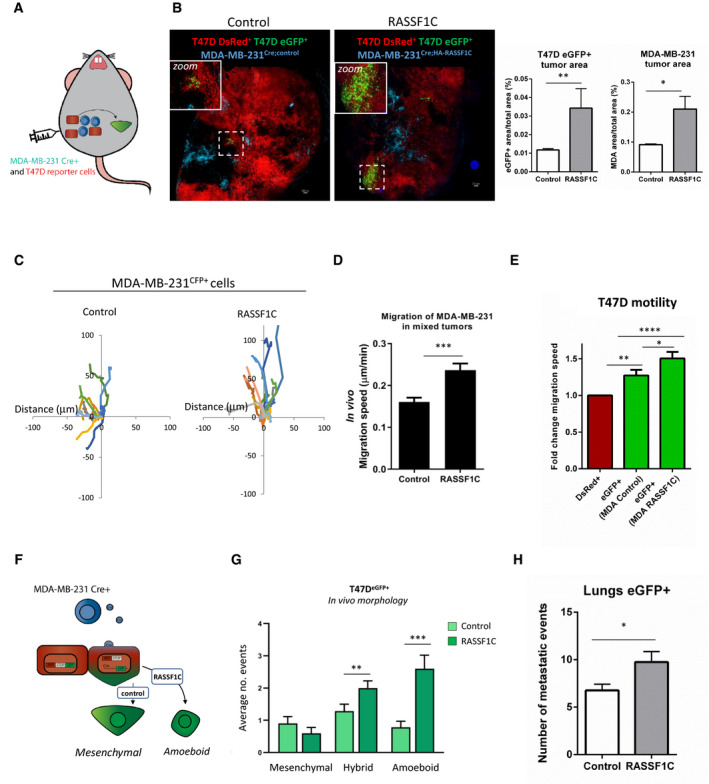
RASSF1C oncogene induces invasion and metastatic dissemination via EV transfer *in vivo* Cartoon of *in vivo* experimental approach. MDA‐MB‐231^CFP;Cre^ and reported T47D^DsRed^ cells were co‐injected in one mammary gland of 10 mice per experiment.Left: representative confocal images of mammary tumor formed by the co‐injection of T47D^DsRed^ reporter cells with either MDA‐MB‐231^CFP;Cre;Control^ or MDA‐MB‐231^CFP;Cre;HA‐RASSF1C^. Scale 200 μm. Right: averaged values for all mice for the percent of the eGFP^+^ or CFP^+^ cells as part of the whole tumor.Representative 3 h migration paths of either MDA‐MB‐231^CFP;Cre;Control^ (Control, left) or MDA‐MB‐231^CFP;Cre;HA‐RASSF1C^ (RASSF1C, right) within a single intravital imaging field of tumors co‐injected with T47D^DsRed^ reporter cells.
*In vivo* migration speed of MDA‐MB‐231^CFP;Cre;Control^ or MDA‐MB‐231^CFP;Cre;HA‐RASSF1C^ in a mixed tumor with T47D^DsRed^ cells. Values are an average of the totals from 5 mice each, measured from intravital time‐lapse images.The normalized *in vivo* motility of T47D^eGFP^ and T47D^DsRed^ reporter cells in mixed tumors. A Non‐parametric Mann‐Whitney U test was used to derive statistical significance.Cartoon representing the Cre‐LoxP model exploited to follow up amoeboid motility in recombined reporter (T47D^eGFP^) cells.Quantification of distinct morphology of T47D^eGFP^ in tumors from observed from a total of 30 positions in *n* = 5 mice/group (Control or RASSF1C). Mesenchymal, amoeboid, or hybrid motility was scored manually as described in Fig 1K.Quantification of the number of T47D^eGFP^ metastatic events found in the lungs of mice with mammary gland tumors. Whole lung tile images were used for quantification, 6 of 10 μm sections each, 100 μm apart. Data information: All data are from *n* = 3 independent experiments. Data are analyzed by Student’s *t*‐test and represented as mean ± SEM.*P ≤ 0.05, **P ≤ 0.01, ***P ≤ 0.001, ****P ≤ 0.0001.

**Figure EV5:**
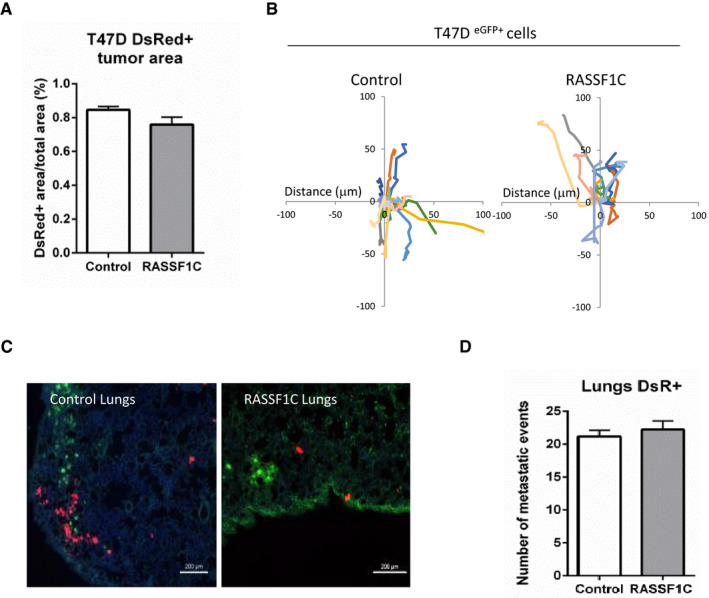
RASSF1C promotes invasion via EV transfer *in vivo* Quantification of the size of the T47D^DsRed^ cells within MDA‐MB‐231^CFP;Cre;Control^ or MDA‐MB‐231^CFP;Cre;HA‐RASSF1C^ tumors indicating equal contribution to tumor volume.Representative 3 h migration paths of T47D^eGFP^ reporter cells within a single intravital imaging field of tumors co‐injected with MDA‐MB‐231^CFP;Cre;Control^ (Control, left) or MDA‐MB‐231^CFP;Cre;HA‐RASSF1C^ (RASSF1C, right).Representative confocal images of lungs of mice with mammary gland tumors initiated by either the co‐injection of T47D^DsRed^ reporter cells with MDA‐MB‐231^CFP;Cre;Control^ or MDA‐MB‐231^CFP;Cre;HA‐RASSF1C^. Scale bars represent 200 μm.Quantification of the number of T47D^DsRed^ metastatic events found in the lungs of mice with mammary gland tumors initiated by MDA‐MB‐231^CFP;Cre;Control^ (Control) or MDA‐MB‐231^CFP;Cre;HA‐RASSF1C^ (RASSF1C) cells. Whole lung tile images were used for quantification, 6 of 10 μm sections each, 100 μm apart. Data information: Data are analyzed by Student’s *t*‐test and represented as mean ± SEM.

To further corroborate our *in vitro* results and three‐dimensional invasion models and validate their relevance *in vivo*, we next employed the previously validated Cre‐LoxP system (Fig 3) in a murine model, to study tumor growth and metastatic potential. The Cre‐LoxP system coupled with intravital imaging allows us to investigate *in vivo* invasive behavior of tumor cells, promoted by transfer of extracellular vesicles. We referred to an already established protocol where a mix of either MDA‐MB‐231^CFP;pUbC‐Cre; Control^ or MDA‐MB‐231^CFP;pUbC‐Cre;RASSF1C^ are injected together with T47D^DsRed^ reporter cells into the mammary glands of mice (Fig 5A and ref. Zomer *et al*, 2015). Resulting tumors were collected and frozen samples were sectioned in 20 μm slides. We did not observe a difference in the total T47D^DsRed^ tumor area, both in the presence of MDA‐MB‐231^CFP;pUbC‐Cre; Control^ and MDA‐MB 231^CFP;pUbC‐Cre;RASSF1C^ cells (Fig EV5A). However, the area of T47D^eGFP^ in MDA‐MB‐231^CFP;pUbC‐Cre;RASSF1C^;T47D^DsRed^ tumors was significantly bigger compared to MDA‐MB‐231^CFP;pUbC‐Cre; Control^;T47D^DsRed^ (Fig 5B images and left bars), suggesting that transfer of RASSF1C‐donor‐derived EVs in the tumor results in higher recombination events, namely reporter cell switch from T47D^DsRed^ to T47D^eGFP^ (Fig 5B, zoomed images), in agreement with our *in vitro* findings (Figs 3A and EV3B). Furthermore, in line with the oncogenic role described for RASSF1C *in vitro*, the area of CFP^+^ MDA‐MB‐231^CFP;pUbC‐Cre;RASSF1C^ bearing tumors was significantly greater compared to control (Fig 5B, right bars). We next evaluated the migratory potential of cells within MDA‐MB‐231^CFP;pUbC‐Cre; Control^ and MDA‐MB‐231^CFP;pUbC‐Cre;RASSF1C^ bearing tumors by time‐lapse intravital microscopy. Randomly selected CFP^+^, eGFP^+^, and DsRed^+^ cells from the same field of view were individually tracked and their migration speed was analyzed. In MDA‐MB‐231^CFP;pUbC‐Cre;RASSF1C^ mixed tumors, RASSF1C CFP^+^ cells showed a higher migration speed compared to cells in control mixed tumors (Fig 5C and D), suggesting that these cells use amoeboid mode of invasion and are characterized by increased migration speed, in agreement with our observations in Fig 1J. Furthermore, RASSF1C‐induced T47D^eGFP+^ reporter cells exhibited the greatest migratory potential compared to unrecombined T47D^DsRed+^ cells or control induced T47D^eGFP+^ cells, once again suggesting that RASSF1C‐driven transfer of EVs has a functional effect also *in vivo* (Figs 5E and EV5B, Movie EV2 for control and Movies EV3 and EV4 for RASSF1C representative cells).

Using manual tracking, we observed that *in vivo* RASSF1C‐driven eGFP^+^ cells also appear to adopt rounded morphology, typical of amoeboid mode (Fig 5F and G, Movie EV2 for control and Movies EV3 and EV4 for RASSF1C), similarly to what we observed for MDA‐MB‐231^CFP;pUbC‐Cre;RASSF1C^ tumors, analyzed in Fig 1K. Finally, we analyzed the ability of T47D^eGFP+^ reporter cells to colonize the lungs. A higher number of metastatic events was apparent in T47D^eGFP+^ cells deriving from the MDA‐MB‐231^CFP;pUbC‐Cre;RASSF1C^;T47D^DsRed^ tumors (Figs 5H and EV5C for representative images), whereas similar numbers of unrecombined T47^DsRed^ cells migrated to the lungs (Fig EV5D).

The presented data support the hypothesis that RASSF1C cells (MDA‐MB‐231 in our model) adopt rounded morphology also in *in vivo* settings and that RASSF1C‐mediated EV transfer occurs also *in vivo* and modulates the fate of less invasive cells (T47D in our model), leading to MAT, accompanied by increased local motility and distal metastatic events.

### Methylation signature of *RASSF1* gene as a prognostic biomarker in breast cancer patients

**Figure 6:**
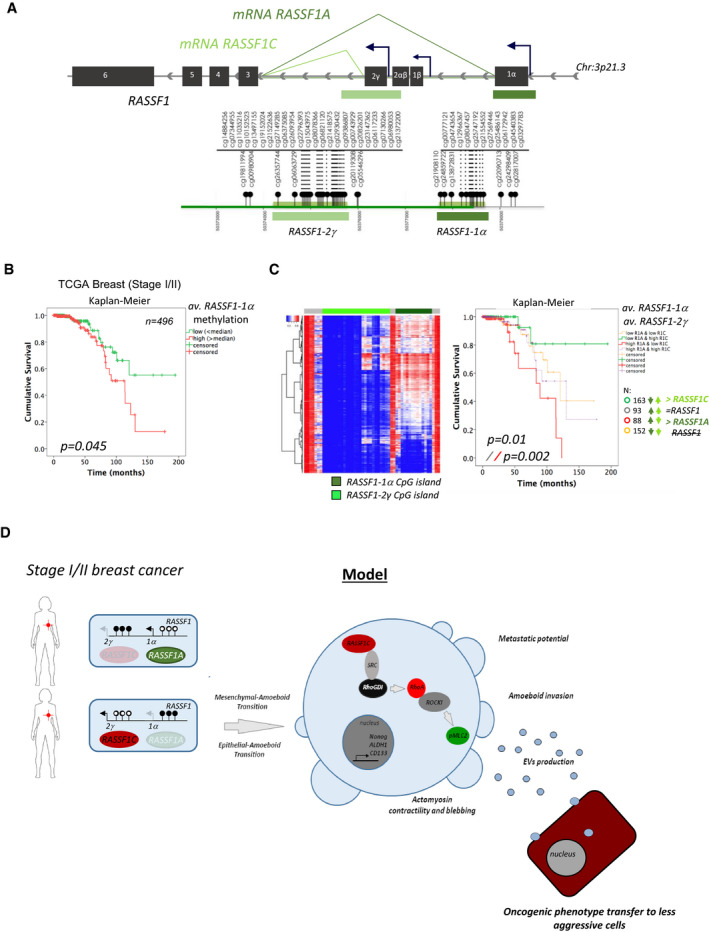
*RASSF1* gene methylation as a prognostic biomarker in breast cancer patients Schematic representation of the *RASSF1* gene showing the domains in *RASSF1A* and *C*. Bars shaded dark and light green denote the CpG islands associated with *RASSF1A* and *RASSF1C* respectively.Kaplan‐Meier plots of overall survival of 496 stage I and II breast cancer patients with different levels of promoter/CpG island methylation of *RASSF1A*, previously reported to be associated with outcome in breast cancer patients. P values were obtained from a log‐rank test.Left: heatmap depicting methylation patterns across the *RASSF1* gene. In light green, RASSF1‐2γ CpG island is depicted; in dark green, RASSF1‐1α CpG island is depicted. Right: Kaplan‐Meyer survival curves depicting interaction of *RASSF1A* and *RASSF1C* methylation to predict the outcome of early‐stage breast cancer patients. P values were calculated from a log‐rank test. Color legend: yellow, low RASSF1A and low RASSF1C; green, low RASSF1A and high RASSF1C; red, high RASSF1A and low RASSF1C; gray, high RASSF1A and high RASSF1C.Model representing how RASSF1C expression in RASSF1A‐silenced breast cancer cells (MDA‐MB‐231) activates a signaling cascade involving SRC kinase, RhoGDI and culminates in RhoA activation and generation of amoeboid contractility and cancer stemness features. RASSF1C‐driven amoeboid cancer stem cells contribute to alteration of the tumor microenvironment by rendering recipient cells (MCF7, T47D) more invasive and able to upregulate stem cell markers, due to EV‐mediated communication.

Hypermethylation of the *RASSF1* gene has been widely associated with poor prognostic outcome in breast cancer patients (Ramos *et al*, 2010). Specific methylation of the *RASSF1‐1α* promoter and loss of the RASSF1A isoform is a frequent event in many tumor types and offers closer association with cancer progression and poor overall survival (Reeves *et al*, 2010; Malpeli *et al*, 2011; Yamashita *et al*, 2018). However, the impact of *RASSF1‐2γ* promoter methylation and expression of RASSF1C has not been previously taken into consideration (Fig 6A). Individual *RASSF1* CpG site analysis allows separation of epigenetic silencing at *RASSF1‐1α* from *RASSF1‐2γ* and therefore study of the differential contribution of the tumor suppressor RASSF1A from the oncogenic RASSF1C isoform to disease progression (Fig 6A, Appendix Fig S3A). To determine whether RASSF1C‐induced amoeboid invasion and tumor progression may be clinically relevant, we interrogated a TCGA cohort of 732 stage I‐IV breast cancer patients, with a particular focus on the early stages (I and II). The TCGA database incorporates tumor methylation signal from 52 CpG sites across the *RASSF1* gene, determined using an Illumina HM450K BeadChip.

We first performed a survival analysis of stage I and II breast cancer patients only considering RASSF1‐1*α* methylation pattern (Fig 6B). The results support the hypothesis that high methylation of *RASSF1‐1α*, indicating reduced RASSF1A expression, is moderately associated with poor breast cancer outcome (*P* = 0.045, log‐rank test) (Fig 6B). Strikingly, *RASSF1‐2γ*, the RASSF1C promoter, also demonstrates a spectrum of low to high methylation, suggesting that methylation of *RASSF1‐2γ* CpGs may effect clinical outcome (Fig 6C, heatmap on the left). In general, breast cancer patients that were alive at follow‐up had lower median methylation of *RASSF1‐1α*, in agreement with the tumor suppressor role of RASSF1A, than those stages I and II patients that had died of breast cancer (Appendix Fig S3B). In line with our data supporting a pro‐oncogenic role for RASSF1C, deceased patients had lower median methylation of *RASSF1‐2γ*, implying that expression from this promoter was active in these tumors (Appendix Fig S3B). Intriguingly, increased methylation of *RASSF1‐1α* (reduced RASSF1A expression) has greater significance in stage I tumors (*P* = 0.000001), whereas reduced methylation *RASSF1‐2γ* (increased RASSF1C expression) has more prognostic influence in late‐stage tumors (*P* = 7 × 10^−6^ Mann–Whitney) (Appendix Fig S3B table). This supports the possibility of an epigenetic switch in *RASSF1* promoter usage from stage I/II to stage III/IV tumors, leading to RASSF1C expression and RASSF1A silencing (Vlahov *et al*, 2015). To determine whether *RASSF1‐2γ* methylation status influences stage I and II tumors, we next divided stage I and II breast cancer patients into four groups based on their methylation status, namely *R1‐1α^Low^:R1‐2γ^Low^*, *R1‐1α^Low^:R1‐2γ^High^*, *R1‐1α^High^:R1‐2γ^Low^*, and *R1‐1α^High^:R1‐2γ^High^*, and repeated the survival analysis from Fig 6B. In line with the above results, *RASSF1‐2γ* methylation (indicating loss of RASSF1C expression) further defines risk groups associated with *RASSF1‐1α* methylation and loss of RASSF1A (*P* = 0.01, log‐rank test, Fig 6C Kaplan‐Meyer curves). Specifically, the extreme survival groups were those with divergent methylation patterns that would support either RASSF1A (5‐yr survival ˜ 90% and 10‐yr survival ˜80%) or RASSF1C expression, (5‐yr survival ˜60% and 10‐year survival ˜10% (*P* = 0.002, log‐rank test, Fig 6C). Patients with convergent *RASSF1* methylation patterns (*R1‐1α^Low^:R1‐2γ^Low^* or *R1‐1α^High^:R1‐2γ^High^*) formed a group with intermediate survival. This indicates that patients with RASSF1C‐expressing tumors were at over 6‐fold higher risk of death than patients likely to express RASSF1A (*P* = 0.02, Appendix Fig S3C).

Taken together, our findings suggest that assessment of the methylation status of both RASSF1‐*1α* and *RASSF1‐2γ* promoters can be a refined tool for breast cancer patient stratification and concurs to provide a more sensitive prognostic signature than the use of RASSF1‐*1α* methylation alone.

## Discussion

The mechanisms underlying metastatic dissemination are largely shaped by the ability of tumor cells to interact with the extracellular environment (Quail & Joyce, 2013b). This is achieved by various mechanisms, by direct contact between cells in the tumor tissue, by transfer of secreted molecules or, as more recently described, via secretion of extracellular vesicles (EVs; Wortzel *et al*, 2019). EVs can be internalized by a recipient cell, through membrane fusion or endocytosis processes, and can thereby alter the host cell’s fate (Van Niel *et al*, 2018). Numerous *in vitro* and *in vivo* studies have shown that vesicle‐mediated exchange of cargo between cancer cells promotes cell migration, invasion (Harris *et al*, 2015; Sung *et al*, 2015), and metastatic events (Costa‐Silva *et al*, 2015; Hoshino *et al*, 2015; Peinado *et al*, 2017).

Oncogenes have been described as key players in the secretion of pro‐invasive EVs. The Rho family member RhoA, one of the major players in amoeboid cell migration, has been previously linked to EV shedding in cancer cells (Li *et al*, 2012; Sedgwick *et al*, 2015). The SRC oncogene has also been described as a modulator of EV release, by direct interaction with ESCRT partner ALIX (Hikita *et al*, 2019) and by acting on syntenin‐syndecan endosomal trafficking (Imjeti *et al*, 2017). Here, we describe for the first time activation of the Rho/ROCK/pMLCII pathway through expression of the RASSF1C oncogene, subsequent activation of the SRC kinase, and inhibitory phosphorylation of the Rho‐GTPase inhibitor, RhoGDI.

RASSF1C‐driven pathway activation leads to morphological changes in mesenchymal and epithelial cells (Fig 1A and B) that can be referred to as mesenchymal and epithelial to amoeboid transition, resulting in cytoskeleton reorganization, higher cell invasion (Fig 1C,F,G, Appendix Fig S1F–I) and *in vivo* dissemination (Fig 1I). Furthermore, RASSF1C oncogenic function results in secretion of greater numbers of EVs, a process likely to be mediated by RhoA (Fig 3B and D).

Building on previous work reported by our group on the interplay of the two major *RASSF1* isoforms, RASSF1C and RASSF1A, with the SRC kinase family (Vlahov *et al*, 2015), here we report a novel function of RASSF1C/SRC in eliciting amoeboid type of motility. SRC has been previously described to modulate RhoA activity through binding and phosphorylation of the Rho GDP dissociation inhibitor, RhoGDI (DerMardirossian *et al*, 2006). Herein we show that, in the presence of RASSF1C, SRC and RhoGDI precipitate in the same complex, and we suggest that the observed upregulation of Rho/ROCK/pMLCII pathway (Fig 2) occurs due to failed Rho inhibition by RhoGDI. Importantly, two point mutations generated in the Ras Association (RA) domain of RASSF1C (a domain shared with the tumor suppressor RASSF1A) fail to activate Rho and promote amoeboid transition (Fig 2F–H). The data suggest that the RA domain, previously shown to be important in RASSF1A direct inhibition of RhoA activity (Lee *et al*, 2016), may also contribute to RASSF1C activation of RhoA.

In agreement with the oncogenic role of RASSF1C (Reeves *et al*, 2010; Volodko *et al*, 2016), here we also report evidence that RASSF1C amoeboid cells release EVs which transfer the invasive phenotype to less invasive cells (Fig 3E–G). RASSF1C‐derived EVs fall in the small EVs category (Fig 3B, Appendix Fig S2C), and their secretion appears to be sustained by RhoA (Fig 3D,F,G). Interestingly, analysis of the RASSF1C EV proteome did not reveal differential enrichment of proteins (Fig EV3D), therefore suggesting that the oncogenic function carried out by RASSF1C EVs could be ascribed to the presence of a specific EV subpopulation, enriched in markers such as CD81, CD63, β‐catenin, PDCD6 (as enrichment of these markers was observed in Fig 3C), or to other types of cargo, such as RNAs or lipids (Zomer *et al*, 2015).

The importance of our *in vitro* results has been translated to *in vivo* settings, where we were able to validate the key role of RASSF1C in promoting tumor progression through EV transfer and amoeboid motility (Fig 5B,D,E). Remarkably, EVs act both locally and via systemic delivery to distal sites in mice, as previously observed (Fig 5H and ref. Zomer *et al*, 2015).

Furthermore, we highlight the relevance of the described biological mechanisms by providing a direct clinical correlation between *RASSF1C* methylation pattern and survival in breast cancer patients (Fig 6C, Appendix Fig S3C).

Clinically relevant data have also been collected for the Rho kinase ROCK (Boyle *et al*, 2020) in a study that unraveled a new axis ROCK‐PERK‐ATF4‐CRELD2 active in invasive breast carcinoma. Interestingly, the work showed that activation of this signaling axis led to CRELD2 enrichment in the secretome of breast cancer cells and CRELD2‐mediated education of cancer‐associated fibroblasts to a tumor‐promoting form. This work provides new insight into tumor‐stroma interactions and, similarly to ours, proposes Rho/ROCK signaling and the ROCK kinase as a converging target for therapeutic intervention in mammary tumors.

A similarly compelling clinical target could be the SRC kinase. Clinical trials for tyrosine kinase (including SRC) inhibitors have shown beneficial outcome for adult patients with Philadelphia chromosome‐positive chronic myeloid leukemia (CML; Cortes *et al*, 2018) and are being tested in combination with EGFR inhibitors for advanced pancreatic cancer (Cardin *et al*, 2018) or alone for the treatment of newly diagnosed endometrial cancer (Duska *et al*, 2019). Evidence that SRC kinase directly governs cancer EV release (Mineo *et al*, 2012) may reinforce the testing of SRC inhibitors in the clinic.

Heterogeneity of tumors suggests that multiple individual cellular phenotypes exist that can both impart resistance to therapy and have variable metastatic potential (Lawson *et al*, 2015). Moreover, cellular plasticity is now appreciated to allow dynamic switching between phenotypes, further increasing tumor heterogeneity. We previously demonstrated that physiological expression of the tumor suppressor RASSF1A upon stem cell differentiation contributes to cell lineage determination (Papaspyropoulos *et al*, 2018) and that, conversely, epigenetic silencing of *RASSF1A* in cancer results in tumor microenvironment remodeling and display of cancer stemness properties (Pankova *et al*, 2019).

RhoA activity has independently been shown to promote stemness via contractility, by inducing nuclear localization of the Hippo pathway mediator YAP1 (Dupont *et al*, 2011) and transcriptional regulation of *OCT4* (Cordenonsi *et al*, 2011).

Using both over‐expression experiments and epigenetic editing, here we show that RASSF1C drives expression of cancer stemness features in amoeboid invasive cells (Fig 4B and H) and upregulates a panel of cancer stem cell markers, such as ALDH1, CD133, and NANOG (Fig 4A,C,D). Furthermore, we show that, as for other RASSF1C‐initiated processes, upregulation of stemness markers such as NANOG relies on RhoA activation (Figs 4E and F and EV4, EV5B–D) and that the same process can be readily initiated by RASSF1C EVs in target cells (Fig 4J).

Our previous results point at a role for RASSF1C in modulating activity of the Hippo pathway effector YAP1, in the absence of RASSF1A (Vlahov *et al*, 2015). Future directions may include exploring the potential link between RASSF1C/RhoA axis and YAP1 function in modulating stemness.

Taken together, our results show for the first time amoeboid motility related to SRC‐mediated activation of the Rho/ROCK/pMLCII pathway via the RASSF1C oncogene, in cancer cells where the tumor suppressor *RASSF1A* is epigenetically silenced (schematic model, Fig 6D). Our results provide new insight on mechanisms of tumor progression, by highlighting the connection between Rho‐driven amoeboid motility, upregulation of cancer stemness features, and transfer of EVs able to alter the fate of target cells and tissues. The presented results correlate methylation data to disease prognosis and provide an explanation for poor clinical outcome in breast cancer patients with *RASSF1A* methylation. Furthermore, the data point at the interplay between Rho and RASSF1C/SRC pathways as a driver of cancer stemness and aggressiveness. Targeting these pathways would harness existing therapies by preventing cancer plasticity, a phenomenon at the basis of disease progression and dissemination.

## Materials and Methods

### Cell culture and transfection

MDA‐MB‐231, MCF7, and H1299 cells were purchased from ATCC and maintained in DMEM supplemented with 10% FBS, 2 mM Glutamine, and 100 U/ml Penicillin/Streptomycin (all from Thermo Scientific). Stable MDA‐MB‐231 Cre^+^ and T47D reporter^+^ cells were made as previously described (for reference: PMID 26658469). T47D cells were cultured in DMEM/F12 supplemented with 10% FBS, 2 mM Glutamine, and 100 U/ml Penicillin/Streptomycin. All cells were maintained at 37°C, with 5% CO_2_ in a humidified incubator. Stable cell lines were kept under constant selection using puromycin (Sigma‐Aldrich) or Zeocin (Thermo Scientific).

### Transfections and stable cell lines

All cells undergoing transfection were plated in the presence of complete DMEM (Thermo Scientific), without antibiotics. All transfections, either with plasmid DNA, were done using the Lipofectamine 2000 transfection reagent (Thermo Scientific) and following the manufacturer’s guidelines.

Stable cells lines were created using the retroviral vector pQXCIP (Clontech). The cDNAs of ZsGreen, ZsGreen‐RASSF1C, or HA‐RASSF1C were cloned into the pQXCIP construct. pQXCIP‐ZsGreen and pQXCIP‐ZsGreen‐RASSF1C were used to generate stable MDA‐MB‐231 and MCF7 cells. Empty pQXCIP or pQXCIP‐HA‐RASSF1C were used to generate stable MDA‐MB‐231 Cre^+^ cells.

### SDS–PAGE and western blot

Cells were lysed for Western blot using Laemmli lysis buffer (2.5 mM Tris–HCl pH6.8, 2% SDS, supplemented with protease inhibitor) before boiling (100°C, 10 min). Protein sample concentration was measured using Bradford reagent, and absorbance was measured using POLARstar OMEGA machine at 595 nm. EVs were lysed in 50 μl RIPA buffer (Cell Signaling) containing 20 mM Tris–HCl (pH 7.5), 150 mM NaCl, 1 mM Na_2_EDTA, 1 mM EGTA, 1% NP40, 1% sodium deoxycholate, 2.5 mM sodium pyrophosphate, 1 mM β‐glycerophosphate, 1 mM Na_3_VO_4_, 1 μg/ml leupeptin. The samples were then boiled at 100°C for 10 min, and 5 μl of each sample was used for protein quantification using the MicroBCA assay (Thermo Scientific) following the manufacturer’s protocol. The absorbance was then measured at wavelength of 562 nm using a plate reader (BMG POLARstar OMEGA). Samples were normalized with 1× Loading buffer (Thermo Scientific), so that equal loading per experiment was achieved. Protein samples were loaded onto a gel and separated by SDS–PAGE using NuPAGE^®^ pre‐cast gels (10% or 4–12%) (Thermo Scientific). Protein was transferred onto PVDF membrane and blocked and incubated with primary antibody in 5% non‐fat milk or BSA diluted in PBS‐Tween 20 (for a complete list of the antibodies used, see Table 1). Secondary antibodies were always incubated in 5% non‐fat Milk. Membranes were covered in ECL solutions from Thermo Scientific, Millipore, or GE Healthcare prior to exposure to film (Fujifilm) and developed in a XoGraph developer.

**Table 1:** Antibodies.

pMLCII	Cell Signaling
Integrin‐β1	Cell Signaling
pSRC (Y527)	Cell Signaling
pSRC (Y416)	Cell Signaling
SRC	Cell Signaling
GAPDH	Cell Signaling
RASSF1C	Abcam
RhoGDI	Abcam
pRHOGDI	Abcam
Rho	Thermo Fisher
HA tag	Millipore
ROCKI	Cell Signaling
Nanog	Cell Signaling
OCT4A	Cell Signaling
CD133	Cell Signaling
pH3	Cell Signaling
Ki‐67	Millipore
ALDH1	Proteintech
ALIX	Cell Signaling
CD81	Santa Cruz
β‐catenin	Cell Signaling
PDCD6	Abcam
Caveolin‐1	Cell Signaling
CD63	Novus biological
CD63 (MEM‐259)	Thermo Scientific
Flag (M2)	Sigma‐Aldrich
GFP	Abcam
Phalloidin‐568	Thermo Fisher
RhoA	Cell Signaling

### 2D Immunofluorescence staining

Cells, plated onto glass coverslips (Fisher), were washed twice with PBS and fixed in 4% (v/v) paraformaldehyde solution for 15 min after which cells were permeabilized with 0.2% (v/v) Triton X solution and blocked with 0.2% (v/v) Fish Skin Gelatin (FSG) (Sigma–Aldrich) for 1 h at RT. Cells were then incubated overnight at 4°C with primary antibody at 1–100 dilution. Coverslips were washed three times with PBS before the Alexa Fluor secondary antibody (1 in 500) (Fisher Scientific) was added and the cells incubated for a further 1 h at RT. Coverslips were washed with PBS and mounted onto microscope slides (Fisher) using Prolong^®^ Gold antifade reagent with DAPI (Thermo Scientific). Fluorescent microscopy was done using a Nikon Ti‐E or a Nikon 2000TE microscope using the NIS elements software (Nikon), version 4.2. Confocal microscopy was done using a Zeiss LSM780 with Zen 2011 program.

### 3D Immunofluorescence staining

Multicellular aggregates were generated for 24 h by using the hanging drop method (Foty, 2011). Spheroids were washed and either seeded on Matrigel matrix for another 48 h in complete medium or mixed with collagen matrix. For 3D spheroids in three‐dimensional collagen staining, collagen‐spheroids gels were washed with PBS, fixed with 4% PFA for 30 min, and cross‐linked in sodium azide solution overnight. After washing with PBS, spheroids in collagen gels were incubated in primary antibodies diluted in 0.2% Triton and 10% NGS overnight, extensively washed with PBS, and incubated in Alexa Fluor secondary antibodies diluted in 0.2% Triton and 10% NGS solution, overnight in 4°C. Spheroids‐collagen gels were washed with PBS and mounted with DAPI. 3D spheroids cultured on Matrigel matrix were washed with PBS, fixed with 4% PFA for 1 h and 3 times washed with PBS:Glycine solution (NaCl, Na_2_HPO_4_, NaH_2_PO_4_, glycine, pH 7.4). Spheroids were permeabilized with 0.5% Triton X‐100 for 10 min, washed 3 times with IF solution (NaCl, Na_2_HPO_4_, NaH_2_PO_4_, NaN_3_, BSA, Triton X‐100, and Tween solution, pH 7.4) and blocked for 2 h in 10% goat serum in IF solution. Spheroids on Matrigel were then incubated with primary antibodies diluted in 10% goat serum and IF solution for 2 h, washed 3 times with IF solution, and incubated in secondary Alexa Fluor antibodies diluted in IF solution for 1 h. Spheroids were then washed with IF solution and mounted with DAPI. Images were captured by using Nikon 10×/0.30 Ph1 objectives.

### In‐gel gelatin zymography

MDA‐MD‐231 cells were counted and 2 × 10^5^ were plated into 24‐well plate. After seeded, cells were washed with PBS and incubated in 300 μl of serum‐free medium for 72 h. Aliquots of the conditioned medium were mixed with 4× sample buffer and loaded for zymography on a 10% SDS–PAGE gel containing 1 mg/ml gelatin as previously published (Pozzi *et al*, 2000). Briefly, gel containing metalloproteases was washed for 1 h in 50 mmol/l Tris–HCl (pH 7.5), 0.1 mol/l NaCl, and 2.5% Triton X‐100 and then incubated at 37°C in 50 mmol/l Tris–HCl (pH 7.5), 10 mmol/l CaCl_2_, and 0.02% sodium azide overnight. The gels were stained with Coomassie blue then destained in 7% acetic acid/5% methanol and imaged for gelatin degradation area.

### Reverse transcriptase and quantitative real‐time PCR (qRT–PCR)

Reverse Transcriptase PCR and Quantitative Real‐Time PCR were prepared using the Power SYBR Green Cells‐to‐Ct Kit (Thermo Scientific) protocol. Cells (2 × 10^5^/well) were plated onto 6 well plates and 2 days later 5 × 10^4^ cells were used for cDNA preparation. Samples were run on Applied Biosystems 7500 Fast real‐time thermal cycler (Applied Biosystems). The protocol used was Holding Step (1× cycle: 95°C 10 min), Cycling Step (50× cycle: 95°C 15 s, 60°C 1 min) and Melt Curve Step (1× cycle: 95°C 15 s, 60°C 1 min, 95°C 30 s, 60°C 15 s). The qRT–PCR primers used are listed in Table 2. 18S or GAPDH were used as internal controls for all the experiments. The calculation of the ΔCt was done on Microsoft Excel.

**Table 2:** RT–PCR primers.

Target gene	Forward primer	Reverse primer
18S	AGTCCCTGCCCTTTGTACACA	GATCCGAGGGCCTCACTAAAC
GAPDH	AATCCCATCACCATCTTCCA	TGGACTCCACGACGTACTCA
NANOG	GGTGTGACGCAGAAGGCCTCA	CCCAGTCGGGTTCACCAGGCA
OCT4	GGCTCGAGAAGGATGTGGTCCG	GGGCTCCCATAGCCTGGGGT
RASSF1A	AGTGCGCGCATTGCAAGTT	AAAGGTCAGGTGTCTCCCAC
RASSF1C	CTGCAGCCAAGAGGACTCGG	GGGTGGCTTCTTGCTGGAGGG
SOX2	GGGGGAAAGTAGTTTGCTGCCTCT	TGCCGCCGCCGATGATTGTT

### EV isolation and treatment of cells

Cells (1 × 10^7^ cells/condition) were grown for the indicated times, up to 3 days, in DMEM supplemented with 0.1% (v/v) FBS (depleted of bovine EVs by overnight centrifugation at 100,000 *g*), 2 mM Glutamine, and 100 U/ml Pen/Strep. Conditioned medium was collected and EVs were isolated by sequential ultracentrifugation at 2,000 *g* for 30 min, 10,000 *g* for 40 min, 100,000 *g* for 3 h in an Optima XPN‐80 (Beckman Coulter) ultracentrifuge using UltraClear Thinwall tubes. The EVs were washed once in 1 ml of PBS and purified by centrifugation at 100,000 *g* for 80 min in an Optima MAX‐XP Ultracentrifuge (Beckman Coulter). EV protein concentration was measured using MicroBCA assay (Thermo Scientific) to ensure equal amounts were added to cells. Unless stated otherwise, 20 ng/μl of EVs were used per well. When stated, prior to addition to cells, EVs were stained using the PKH67 Green Fluorescent Cell Linker Mini Kit (Sigma–Aldrich) following manufacturer’s protocol. Internalization of EVs was assessed by confocal microscopy at a Zeiss LSM780 microscope.

### EV isolation by tangential flow filtration/size exclusion chromatography

Cells were handled as described under ‘EV Isolation’. EVs were isolated from ˜80 ml conditioned medium as previously described (Willms *et al*, 2016). Briefly, the 10,000 *g* supernatant was concentrated down to 1.0 ml with a 10 kDa molecular weight cut‐off Vivaflow 50 R tangential flow (TFF) device (Sartorius) and a 10 kDa Amicon Ultra‐15 centrifugal filter unit (Merck Millipore, Billerica, Massachusetts, USA). The concentrated supernatant was loaded on a Tricorn 10/300 Sepharose 4 Fast Flow (S4FF) size exclusion chromatography column (GE Healthcare, Buckinghamshire, UK) connected to the ÄKTA prime system (GE Healthcare), and eluted at 0.5 ml/min flow rate using PBS as the eluent. Chromatogram was recorded using absorbance at 280 nm. 1.0 ml fractions were collected, and EV‐containing fractions were pooled and concentrated with a 10 kDa molecular weight cut‐off Amicon Ultra‐4 centrifugal filter unit (Merck Millipore).

### Nanoparticle tracking analysis

For this analysis 1/5 of the total EV pellet per sample was resuspended in 10 μl PBS (Gibco) and stored at 4°C for up to 7 days. Additional 990 μl PBS were added on the day of analysis and the EV pellet was vigorously resuspended by pipetting and kept on ice, before starting the analysis.

Before starting any measurement, NanoSight NS300 (Malvern Panalytical) was washed three times by loading distilled water onto a syringe pump using a 1 ml syringe and pressing the liquid into the flow‐cell top plate of the NanoSight. PBS was used to prime the instrument and to control the purity of the diluent (i.e., absence of particulate in the solution or presence of particulate in a concentration lower than detectable level). After priming, 1 ml of sample was carefully loaded on the syringe pump. Every measurement was done automatically, with the aid of the syringe pump and a script for data acquisition was generated on the NTA 3.2 software. Three recordings of 60 min each were automatically taken once each sample was loaded in the chamber and the focus on the particles in solution was adjusted manually. Additionally, every sample was loaded onto the chamber twice and an average of 6 recordings was then used as a final value. NTA 3.2 software was used to generate data output.

### Time‐lapse imaging

MCF7 cells (3 × 10^5^ cells/well) were plated onto 12‐well plates. Cells were incubated for 24 h before media was changed to complete DMEM/F12 media without phenol red. The cells were then imaged for up to 8 h at a rate of 1 picture every 15 min, using a Nikon TE2000 inverted microscope at 20× magnification at 37°C and 5% CO_2_. The collected pictures and videos were analyzed using Nikon NIS Elements 4.0 program. Where necessary, still images were used at 0 h and 12 h to quantify the number of rounded, spherical cells per field of view. The diameter was also quantified using Nikon NIS Elements 4.0.

### Fluorescence‐assisted cell sorting

Fluorescence‐assisted cell sorting (FACS) of Green^high^ cells was performed as follows. Briefly, cells (1 × 10^6^) grown in 2D were trypsinized and washed in PBS. Cells were resuspended in PBS containing 1% FBS. Cells were gated and sorted for high green fluorescence on a BD FACSAria III. Sorted cells were re‐plated and grown in full medium. Wild type MDA‐MB‐231 cells were used as control for the setup and the definition of the fluorescence threshold.

### Immunoprecipitation

1 × 10^6^ cells per condition were transfected with the described plasmids, as stated in ‘Transfection methods’. Cells were washed two times with PBS before lysis with immunoprecipitation buffer (20 mM Tris–HCl pH 7.4, 1% NP40, 100 mM NaCl, 0.5 mM EDTA, 1× protease and 1× phosphatase inhibitors (Roche), 10% v/v glycerol). Cells were scraped from the dish and collected into a 1.5 ml microfuge tube and incubated end‐over‐end at 4°C for 10 min. Lysates were then cleared by centrifugation (20,817 *g*, 20 min, 4°C). Protein concentration of the lysates was determined by Bradford assay, so that an equal amount of protein was loaded into each immunoprecipitation reaction.

Protein A Dynabeads (Millipore) were used for immunoprecipitation. All beads were washed 3 times in immunoprecipitation buffer before being aliquoted into 1.5 ml microfuge tubes. Each lysate was pre‐cleared with 10 μl Protein A Dynabeads for 30 min prior to immunoprecipitation. 1 μg of antibody or corresponding amounts of same species IgG (Cell Signaling) were added to 20 μl of protein A Dynabeads and incubated end‐over‐end at 4°C for 30 min before lysate was added to the tubes and incubated end‐over‐end at 4°C for 3 h. Samples containing protein A Dynabeads were washed three times by immobilizing the beads using a magnet, aspirating the supernatant, and resuspending the beads. Protein was removed from the antibody by boiling at 100°C for 5 min in 2× LDS Sample buffer (Life Technologies). An equal volume of each sample and its corresponding input was loaded onto a gel for electrophoresis and Western blot analysis.

### Migration assay with xCELLigence

xCELLigence migration assay allows measurement of cells migrating from the upper chamber through a membrane into the bottom chamber of a Boyden‐like system in response to chemoattractant. 160 μl DMEM containing 10% FBS were added to the bottom wells of xCELLigence Real‐Time Cell Analyzer CIM plate (Roche), as chemoattractant. The top chamber was then attached to the bottom chamber and 50 μl of DMEM without supplements were added to each well. The plate was first incubated for 30 min at 37°C and then loaded into the xCELLigence analyzer and a blank run was run to zero the machine. EVs from described conditions were resuspended in 50 μl sterile PBS (Gibco) and normalized according to BCA assay (Thermo Fisher), in order to equally load 20 or 40 ng/μl of EVs per well. 50,000 cells were resuspended in DMEM without supplements, with or without EVs. Cells were plated into the top chamber and allowed to settle for 30 min at RT before the plate was loaded into the xCELLigence analyzer. Cells were incubated in the machine at 37°C, 5% CO_2_ in a humidified incubator, and measurements were taken every 15 min for 24 h. Normalization was calculated by subtracting the invasion rate of untreated MDA‐MB‐231 cells to the invasion rate of EV‐treated MDA‐MB‐231 cells.

### 3D Spheroids formation and morphology in 3D matrix

MDA‐MB‐231 cancer multicellular spheroids were generated by using the hanging drop method (Foty, 2011). Briefly, ZsGreen‐Control or ZsGreen‐RASSF1C transfected cells were detached with 2 mmol/l EDTA, counted, resuspended in DMEM supplemented with methylcellulose (20%, Sigma) and GFR Matrigel matrix (1%, Corning) and incubated as droplets (25 μl) containing 1,000 cells for 24 h to generate multicellular aggregates. Three‐dimensional aggregates were washed with medium, and either seeded and cultured on Matrigel matrix for 48 h or mixed with rat tail collagen (Serva; 2.0 mg/ml), 10× PBS, 1 M NaOH, and complete medium. Collagen gel‐spheroids solution was pipetted as a 100 µl drop‐matrix suspension into 12‐wells, polymerized at 37°C, replaced with medium, and cultured for another 24 h till single‐cell invasion from spheroids was apparent. Single‐cell morphology apparent as mesenchymal (elongated) projections from the ZsGreen transfected spheroids or as amoeboid (rounded) single cells invaded around from ZsGgreen‐RASSF1C transfected spheroids was imaged and captured by using Nikon 20×/0.8 Ph1 objective.

### Boyden chamber invasion and Hydrogel invasion assays

MDA‐MB‐231 or MCF7 cancer cells were pre‐treated or not with either 10 µmol/l of ROCK inhibitor Y27632 for 1 h or with 2 µg/ml Rho inhibitor C3 for 4 h. Cells were trypsinized and (1 × 10^5^) were cultured in serum‐free medium alone with 20 µmol/l GM6001 metalloprotease inhibitor, or incubated with EVs (20 or 40 ng/µl) in the upper wells (in triplicate) of Transwell Matrigel Boyden chambers (Corning), or seeded in upper wells coated with 50% hydrogel matrix (PuraMatrix, Corning). Cells were allowed to invade toward bottom wells supplemented with 10% FBS medium. After 8 or 18 h of incubation, invading cells were fixed and stained with Richard‐Allan Scientific™ Three‐Step Stain (Thermo Fisher Scientific), photographed, and counted manually using Adobe Photoshop software. Images were captured by Nikon Ti90 4× objective.

### Single‐cell morphology in 3D collagen

To analyze single‐cell morphology in 3D collagen, cells were trypsinized, washed in with complete medium, counted and (10^5^/ml) cells were mixed with a solution containing 2 mg/ml Collagen R (Serva), 10× PBS, 1 M NaOH, and complete medium. The suspension of cells was loaded in 96 wells. The gels were polymerized at 37°C for 30 min and replaced with complete medium. After 24 h cell morphology in 3D collagen was analyzed by using a Nikon Eclipse TE2000 (10×/0.40 Ph1 objective) microscope, counted, and classified on the basis of the elongation index. The elongation index was calculated as the length divided by the width. For each condition, a minimum of 500 cells were analyzed.

### Immunoblotting and Rho pull‐down assay

Confluent cell cultures were washed with cold 1× PBS and lysed in modified RIPA buffer (50 mM Tris–HCL (pH 7.4), 150 mM NaCL, 5 mM EDTA, 1% NP40, 1% sodium deoxycholate, 50 mM NaF and 1% aprotinin and 0.1 mM Na_3_VO_4_). Rho pull‐down assays were performed using GST‐Rhotekin and Rho pull‐down detection kit according to manufacturer’s instructions (Thermo Scientific). The levels of total and active Rho were established by using a Rho‐specific antibody. Protein concentration in lysates was determined by BCA assay. For immunoblotting, samples were separated on 12% SDS‐polyacrylamide gels and transferred onto nitrocellulose membrane. Non‐specific activity was blocked by 1× TBS containing 0.05% Tween and 4% bovine serum albumin (BSA). Membranes were incubated overnight in Rho primary antibody, washed, and then incubated within HRP‐conjugated secondary antibody for 1 h at RT. After extensive washing with TBS‐T, blots were developed as previously stated.

### CRISPR

Plasmids containing a mammalian codon‐optimized dCas9‐VP64 activator (pMLM3705, AddGene #47754) and single‐chain gRNA encoding plasmid (MLM3636, AddGene #43860) were gifts from Keith Joung. Plasmids encoding dCas9‐only and dCas9‐Super Krab Domain (SKD) repressor were gifts from Marianne Rots (Cano‐Rodriguez *et al*, 2016). gRNAs were cloned into BsmBI‐digested MLM3636 plasmid by ligation of annealed oligonucleotides encoding 20 bp gRNA target sequences and 4 bp overhangs. Four target regions of 20 bps of the RASSF1C promoter were selected to design gRNAs based on close proximity to the transcription start site (TSS) (gRNA1: TTGTGCGCTTGCCCGGACGC; gRNA2: CGGAGCGATGAGGTCATTCC; gRNA3: GGATCTAGCTCTTGTCTCAT reverse strand; gRNA4: AGTGCGCGTGCGCGGAGCCT reverse strand). For dCas9 experiments, each well of a 12‐well plate was transfected with 500 ng of dCas9 construct and 500 ng combined gRNAs. Transfected cells were collected 48 h post‐transfection.

### Co‐cultures and transwell experiments

For all co‐culture and transwell experiments, complete DMEM culture medium was used. MDA‐MB‐231^CFP; Cre^ cells were co‐cultured with T47D cells for one week prior to imaging in a culture dish. All experiments were done in parallel. For the transwell experiments, transwells with a pore size of 400 nm (Greiner Bio‐one, Frickenhausen, Germany) were placed in a culture dish with T47D cells for one week prior to imaging. The cells in the culture dish were imaged with a Nikon 2000TE microscope using a 20× objective. During imaging, cells were kept at 37°C in a humidified atmosphere containing 5% CO2. A 430/24 nm excitation filter and a 470/40 nm emission filter was used for CFP, a 470/40 nm excitation filter and a 520/40 nm emission filter was used for eGFP, and a 572/35 nm excitation filter and a 640/50 nm emission filter was used for DsRed.

### Mice

NOD scid gamma (NSG) mice (own crossing) were housed under IVC conditions. Mice received food and water ad libitum. All experiments were carried out in accordance with the guidelines of the Animal Welfare Committee of the Royal Netherlands Academy of Arts and Sciences, The Netherlands.

### Injection of tumor cells

One week before injection of tumor cells, mice were ovarectomized and implanted with an estrogen pellet (0.36 mg/pellet, 60‐day release, Innovative Research of America, Sarasota, FL, USA) using a precision trochar, as per the manufacturer’s instructions. Tumor cells were injected in the fourth and/or ninth mammary fat pad of female 8–20 weeks old NSG mice. For MDA‐MB‐231 tumor development, 5 × 10^5^ MDA‐MB‐231 cells in sterile PBS were injected. For MDA‐MB‐231 and T47D mixed tumors, a total of 1 × 10^6^ cells was injected in a ratio of 10:1 Cre^+^:reporter^+^ cells in sterile PBS with 50% growth factor‐reduced Matrigel (BD Biosciences, Franklin Lakes, NJ, USA).

### Tumor and tissue processing

Tumors and tissues were removed from the mice at the end of the experiment and fixed and processed as described before (for reference: PMID 26658469). In short, tissues were fixed in periodate‐lysine‐paraformaldehyde (PLP) buffer (2.5 ml 4% PFA + 0.0212 g NaIO_4_ + 3.75 ml L‐Lysine + 3.75 ml P‐buffer (pH 7.4)) O/N at 4°C. The following day, the fixed tumors and tissues were washed twice with P‐buffer and placed for at least 6 h in 30% sucrose at 4°C. The tumors and tissues were then embedded in tissue freezing medium (Leica Microsystems, Nussloch, Germany) and stored at −80°C before cryosectioning.

### Immunostainings and confocal microscopy of tissue sections

Tumor or tissue cryosections (15 μm thick when used for subsequent immunostainings) were rehydrated for 10 min in Tris 0.1 M pH 7.4, when indicated used for immunostainings and embedded in Vectashield mounting medium (hard set; Vector Labs, Burlington, Ontario, Canada). When indicated, cryosections were counterstained with 0.1 μg/ml DAPI (Invitrogen Life Technologies, Paisley, UK) to visualize the nuclei. Images were acquired using Nikon Ti‐E microscope equipped with 40× dry objectives. DAPI and CFP were excited with a UV 405 nm laser, and emission was collected at 415–455 nm for DAPI and 455–495 nm for CFP. eGFP was excited with an argon ion laser at 488 nm and emission was collected at 490–515 nm. DsRed was excited with a 561 nm laser and emission was collected at 570–620 nm. At least 6 frozen sections per mouse were analyzed. Area and number of primary tumors and metastatic events to the lungs were quantified using Nikon NIS software.

### Intravital imaging

Mice were sedated using isoflurane inhalation anesthesia (1.5–2% isoflurane/O_2_ mixture). The imaging site was surgically exposed, and the mouse was placed with its head in a facemask within a custom‐designed imaging box. The isoflurane was introduced through the facemask and ventilated by an outlet on the other side of the box. The imaging box and microscope were kept at 36.5°C by a climate chamber that surrounds the whole stage of the microscope including the objectives. Imaging was performed on an inverted Leica TCS SP5 AOBS or Leica SP8 multi‐photon microscope (Mannheim, Germany) with a chameleon Ti:Sapphire pumped Optical Parametric Oscillator (Coherent Inc. Santa Clare, CA, USA). The microscope is equipped with four non‐descanned detectors: NDD1 (< 455 nm), NDD2 (455–490 nm), NDD3 (500–550 nm), NDD4 (560–650 nm). CFP was excited with a wavelength of 840 nm, and GFP and DsRed were excited with a wavelength of 960 nm. CFP was detected in NND2, GFP was detected in NDD3, and DsRed in NDD4. All images were in 12 bit and acquired with a 25× (HCX IRAPO N.A. 0.95 WD 2.5 mm) water objective. All pictures were processed using ImageJ software or Imaris 8.2 (Bitplane); pictures were converted to an 8 bit RGB, smoothed (if necessary), cropped (if necessary), rotated (if necessary), and contrasted linearly. At the beginning of each movie, a random DsRed^+^ cell close to an eGFP^+^ was selected. The XY position was determined over time, and the displacement and track distance were calculated by Excel (Microsoft).

### *In silico* analysis

Breast cancer data, including clinical and methylation was downloaded from TCGA database (Broad Institute TCGA Genome Data Analysis Center (2015): Analysis‐ready standardized TCGA data from Broad GDAC Firehose stddata__2014_07_15 run. Broad Institute of MIT and Harvard. Dataset. https://doi.org/10.7908/C1TQ60P0). A total of 732 patients had HM450K tumor methylation data. Clinical data were available for 695 patients, 687 of which were females, with an average age at diagnosis 58 years (± 13 years). Tumor stage was determined for all 687 female patients, whereby 114 patients were stage I at primary diagnosis, stage II – 384 patients, stage III – 176 patients, and stage IV – 7 patients. Estrogen receptor (ER) status was determined for 656 primary tumors, whereby 498 were ER‐positive and 150 were ER‐negative. Male patients were excluded from analyses. Molecular subtype (PAM50) was available for 209 tumors, whereby 107 had Luminal A subtype, 43 Luminal B, 40 Basal, 14 Her2^+^, and 5 normal‐like.

The CpG island methylation was calculated as the average methylation of CpG sites located within defined CpG island at Genome Browser (http://genome‐euro.ucsc.edu/index.html). The median values were calculated for all 687 female patients.

### Statistics

For all *in vitro* experiments, unless stated otherwise in figure legends, statistical analysis was carried out using Student’s *t*‐test, calculated on Microsoft Excel or Prism 6.0 (GraphPad). The standard error mean (SEM), used for error bars, was calculated by the division of the standard deviation by the square root of the number of measurements made. For *in silico* analysis, statistics were performed using SPSS 24.0 and R version 3.3.2. The heatmaps were created using the ggplot package. A Mann‐Whitney‐Wilcoxon test was used to compare two different sample populations. Statistical significance was regarded as *P* < 0.05.

## Author contributions

DP, MLT, NV, and SSc performed all *in vitro* work; NV and SSt performed all *in vivo* work and statistical measurements under guidance of JvR; ME and DC‐R employed the CRISPR‐dCas9 experiments under the guidance of MGR. EW and MLT performed SEC isolation under the supervision of MJW. AMG performed *in silico* analysis on TCGA retrieved data with the help of CK. AvK performed all proteomic experiments, EON designed and supervised all experiments, conceptualized the story and wrote the manuscript with the help of DP and MLT.

## Conflict of interest

MJW is co‐founder and non‐executive director of and has equity interest in Evox Therapeutics. All other authors declare that they have no conflict of interest.

## Supporting information



Appendix

Expanded View Figures PDF

Movie EV1

Movie EV2

Movie EV3

Movie EV4

Source Data for Expanded View and Appendix

Source Data for Figure 1

Source Data for Figure 2

Source Data for Figure 3

Source Data for Figure 4

## Data Availability

The mass spectrometry raw data included in this publication have been deposited to the ProteomeXchange Consortium via the PRIDE partner repository with the dataset identifier: PXD026226 (http://www.ebi.ac.uk/pride/archive/projects/PXD026226).
