# Supplementary material for: RASSF1C oncogene elicits amoeboid invasion, cancer stemness, and extracellular vesicle release via a SRC/Rho axis
Source: EMBO J. 2021 Sep 17;40(20):e107680. doi: 10.15252/embj.2021107680 (PMC8521318; doi:10.15252/embj.2021107680)
Supplement: Supplementary file 1 — Appendix [file EMBJ-40-e107680-s012.pdf]

Appendix Figures

Index

Appendix Figure S1	2
Appendix Figure S2	4
Appendix Figure S3	5

Appendix Figure S1

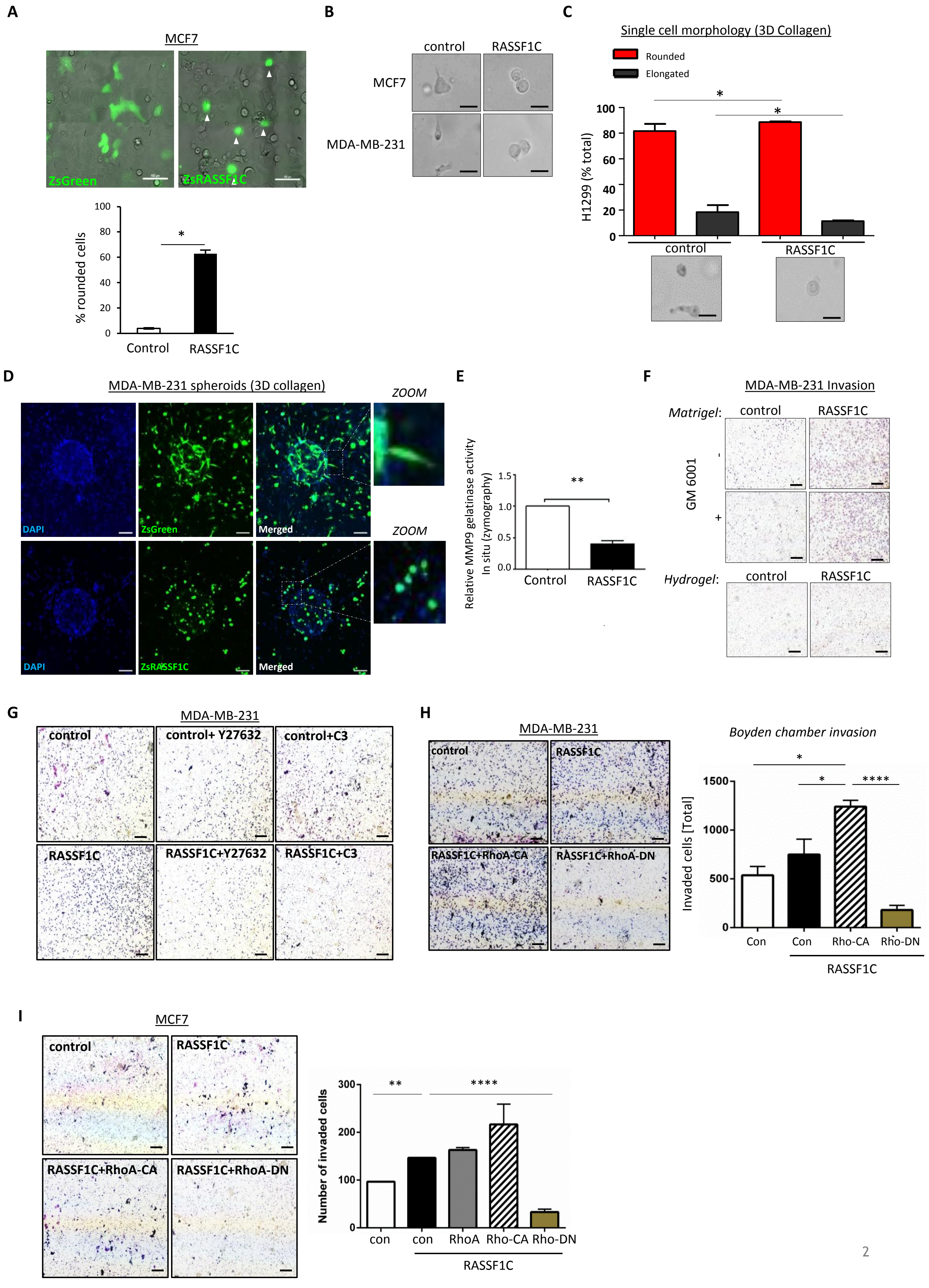

Appendix Figure S1 RASSF1C promotes invasion. Related to Figure 1.

A. Top: images taken 48 h after MCF7 cells were transfected with ZsGreen or ZsGreen-RASSF1C (ZsRASSF1C) plasmids. White arrowheads show rounded morphology of ZsGreen-RASSF1C cells. Scale bars 100 µm. The images were taken as tile scans and stitched together into mosaics. Representative 4 x 4 scan areas of whole tile scans are presented here. Bottom: quantification of $ZsGreen^{+}$ cells with rounded morphology as percentage of the total number of $ZsGreen^{+}$ cells.
B. Related to Fig 1B, representative images of single cell morphology of MCF7 and MDA-MB-231 cell lines, expressing a ZsGreen (control) or ZsGreen-RASSF1C construct, cultured in 3D rat tail collagen I and analysed for their rounded or elongated (fibroblast-like) morphology, 24 hours after seeding in 3D matrix. Scale bar 10 µm.
C. Quantification (top) and representative images (bottom, scale bar 10 µm), showing single cell morphology in 3D-collagen matrix of H1299 cells, 12 hours after being transfected with ZsGreen (Control) or ZsRASSF1C (RASSF1C) plasmids.
D. Immunofluorescent images of ZsGreen or ZsRASSF1C expressing MDA-MB-231 spheroids cultured in 3D rat tail collagen I. Zoom images showing detail of single cells morphology adapted during invasion from 3D spheroids. Scale bar 100 µm.
E. Quantification of in situ zymography assay, related to Fig 1E.
F. Related to Fig 1F, representative images of 3D Matrigel Boyden chamber invasion with or without metalloproteases inhibitor GM6001 and 3D Hydrogel invasion of MDA-MB-231 control cells or cells over-expressing RASSF1C. Scale bar 200 µm.
G. Related to Fig 1G, representative images of Boyden chamber invasion in 3D Matrigel of MDA-MB-231 cells, transfected with Control or RASSF1C and treated with inhibitors against ROCK (Y27632, 10 µmol/L) or Rho (C3, 2 µg/ml). Scale bar 200 µm.
H. Representative images and quantification of Boyden chamber invasion in 3D matrigel of MDA-MB-231 cells, transfected with Control or RASSF1C and RhoA catalytically active (CA) or dominant negative (DN) plasmids. Scale bar 200 µm.
I. Representative images and quantification of Boyden chamber invasion in 3D matrigel of MCF7 cells, transfected with Control or RASSF1C and RhoA catalytically active (CA) or dominant negative (DN) plasmids, Scale bar 200 µm.

Data information: Data are analyzed by *Student T-test* and represented as mean ± SEM.* $p \leq 0.05$, ** $p \leq 0.01$, **** $p \leq 0.0001$, n=3.

Appendix Figure S2

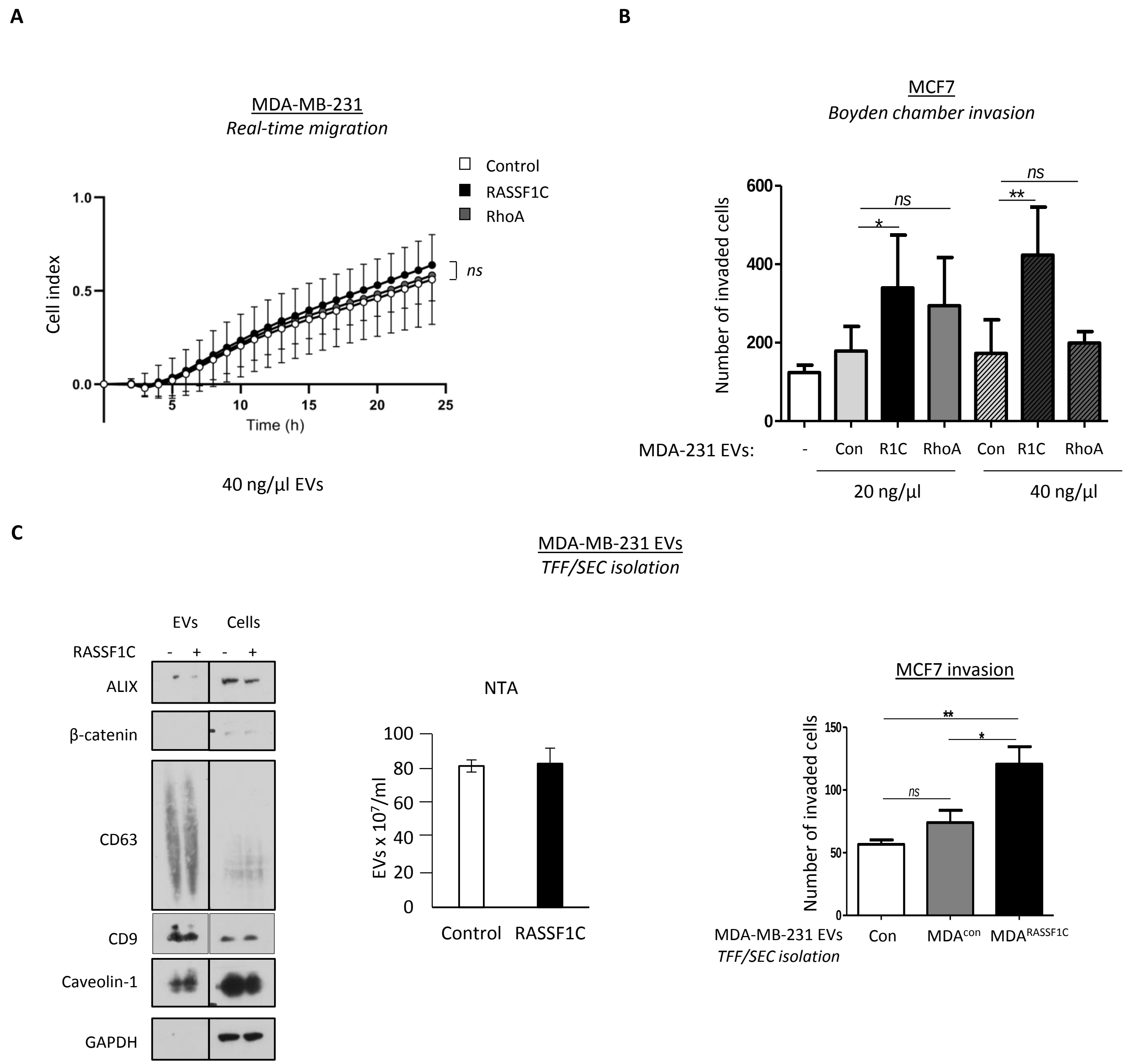

Appendix Figure S2 RASSF1C increases EV-promoted invasion *in vitro*. Related to Figure 3.

A. Related to Fig 3E, normalized values of real-time migration rates in xCELLigence plates of MDA-MB-231 cells treated with 40 ng/µl EVs derived from MCF7 cells expressing a Control, FLAG-RASSF1C or EGFP-RhoA plasmid, left to migrate for 24 h. Statistics were performed with 2-way ANOVA (Dunnett's multiple comparisons test).
B. Related to Fig. 3f, quantification of Boyden chamber invasion assay in 3D matrigel of MCF7 cells, treated with 20 or 40 ng/µl EVs derived from MDA-MB-231 cells, transfected the same constructs used in Fig 3E.
C. EVs from MDA-MB-231 cells transiently expressing a Control or a Flag-RASSF1C construct were purified by tangential-ultrafiltration (TFF) followed by Size Exclusion Liquid Chromatography (SEC), to ensure purity of the preparations. Left: Western blot analysis of EVs isolated from MDA-MB-231 cells transfected with Control (pcDNA3) or FLAG-RASSF1C with indicated antibodies. Equal amounts of protein were loaded after normalization via microBCA assay, and middle: averaged Nanoparticle Tracking Analysis displaying EV concentration for both conditions. Right: equal amounts of MDA-MB-231 EVs were incubated with MCF7 cells in Boyden chamber invasion assay. Quantification of the number of invaded cells in the presence and absence of EVs is represented.

Data information: Data are analyzed by *Student T-test* and represented as mean ± SEM.* $p \leq 0.05$, ** $p \leq 0.01$, n=3.

Appendix Figure S3

A

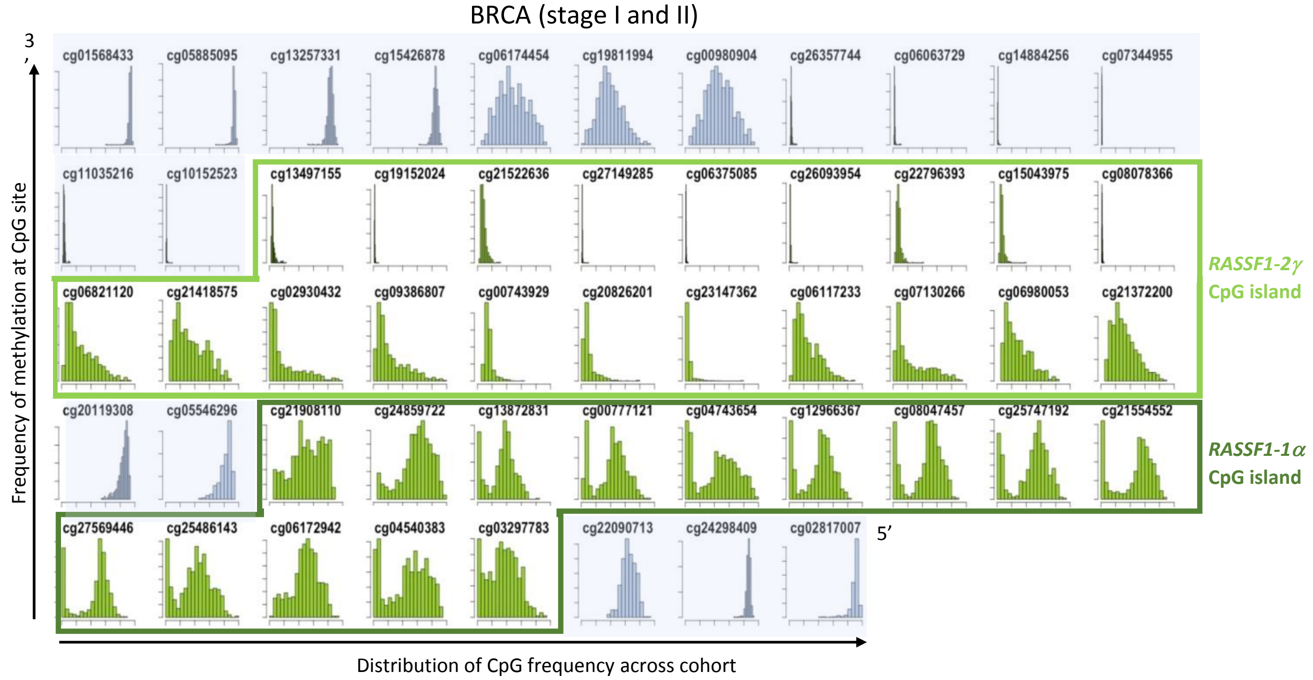

B

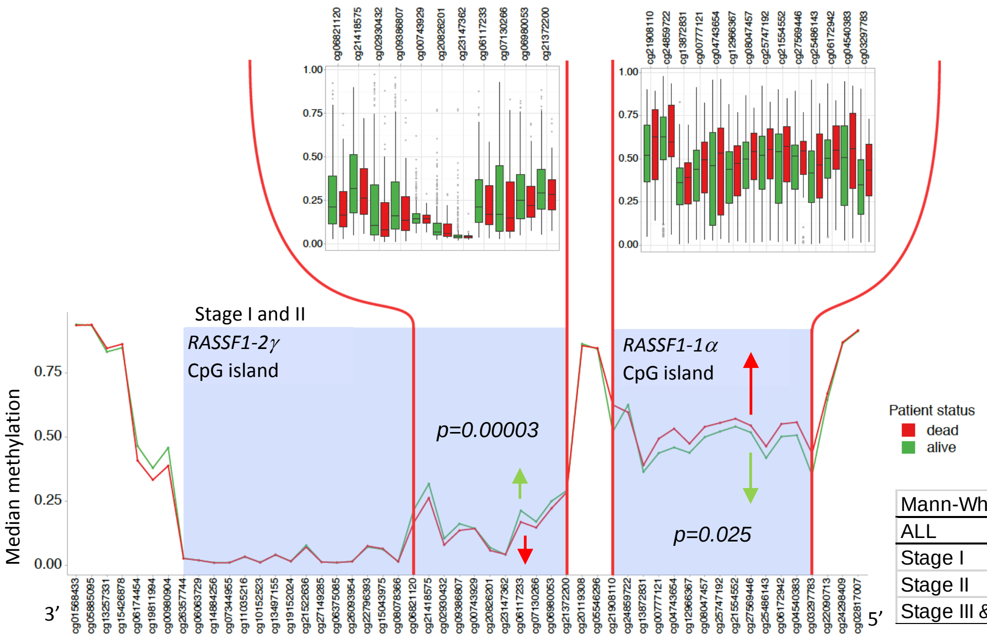

Median methylation [alive vs dead]

Mann-Whitney test	*RASSF1-1 a*	*RASSF1-2 g*
ALL (n=687)	*p=0.00003*	*p=0.025*
Stage I (n=114)	*p=0.000001*	*n.s.*
Stage II (n=384)	*p=0.003*	*p=0.03*
Stage III & IV (n=183)	*n.s.*	*p=0.00000007*

C

Cox regression [multivariate]

CpG island	Transcript	Analysis	df	*p value*	RR	95% CI
RASSF1	R1A and R1C	high *vs* low	2	**0.03**	-	-
RASSF1-1α<br>RASSF1-2γ	R1A and R1C	low, high *vs* low, low/high, high	1	0.16	2.83	(0.66-12.12)
RASSF1-1α<br>RASSF1-2γ	R1A and R1C	low, high *vs* high, low	1	**0.02**	**6.62**	**(1.38-31.77)**
		pN +ve *vs* -ve	1	0.23	1.58	(0.75-3.32)

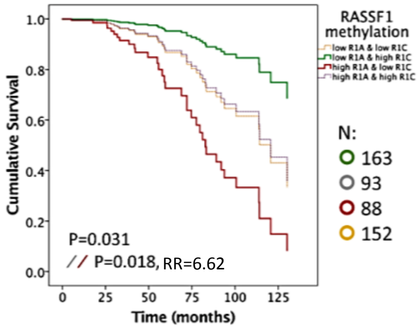

Appendix Figure S3 Methylation pattern of RASSF1 gene is associated with prognostic outcome in breast cancer patients. Related to Figure 6.

A. Histograms depicting distribution of methylation across stage I and II breast cancer tumors for individual *RASSF1* HM450K CpG sites located within CpG islands (green) or CpG shores (blue).
B. Distribution of median methylation of all individual *RASSF1* CpG sites in tumors of stage I and II breast cancer patients who were alive (green) or dead (red) on last follow up. Boxplots (n = 498, of which 114 - stage I and 384 - stage II patients) represent individual values of the median methylation of patients in the area that gives greatest prognostic value (boxes extend from the 25th to 75th percentiles, central band represents the median value, whiskers span from smallest value up to the largest). Table summarizes the differences in median methylation of *RASSF1A* and *RASSF1C* CpGs according to tumor stage, with a particular focus on stage I and II.
C. Related to Fig 6C. Left: table summarizing Cox multivariate analysis performed on the same data-set as in Fig 6C. Right: Cox survival curves depicting interaction of RASSF1A and RASSF1C methylation to predict the outcome of early stage breast cancer patients. P values were calculated from a log-rank test. Color legend: yellow, low RASSF1A and low RASSF1C; green, low RASSF1A and high RASSF1C; red, high RASSF1A and low RASSF1C; grey, high RASSF1A and high RASSF1C.

Data information: Abbreviations used in the table: df, degrees of freedom; CI, confidence interval; RR, relative risk; pN, pathologic node status.
