# Supplementary material for: RASSF1C oncogene elicits amoeboid invasion, cancer stemness, and extracellular vesicle release via a SRC/Rho axis
Source: EMBO J. 2021 Sep 17;40(20):e107680. doi: 10.15252/embj.2021107680 (PMC8521318; doi:10.15252/embj.2021107680)
Supplement: Supplementary file 3 — Movie EV1 [file EMBJ-40-e107680-s006.zip › Movie EV1.docx]

**Movie EV1**

Example of ZsRASSF1C expressing MCF7 cell that became rounded, a morphological change associated with increased contractility. The cells were imaged for up to 8 h at a rate of 1 picture every 15 min.
