# Supplementary material for: RASSF1C oncogene elicits amoeboid invasion, cancer stemness, and extracellular vesicle release via a SRC/Rho axis
Source: EMBO J. 2021 Sep 17;40(20):e107680. doi: 10.15252/embj.2021107680 (PMC8521318; doi:10.15252/embj.2021107680)
Supplement: Supplementary file 4 — Movie EV2 [file EMBJ-40-e107680-s008.zip › Movie EV2.docx]

**Movie EV2**

Representative intravital imaging video of MDA-MB-231^CFP;Cre;Control^/T47D^DsRed^ tumors where eGFP^+^ recombined reporter cells adopt mesenchymal mode of motility.
