# Supplementary material for: RASSF1C oncogene elicits amoeboid invasion, cancer stemness, and extracellular vesicle release via a SRC/Rho axis
Source: EMBO J. 2021 Sep 17;40(20):e107680. doi: 10.15252/embj.2021107680 (PMC8521318; doi:10.15252/embj.2021107680)
Supplement: Supplementary file 5 — Movie EV3 [file EMBJ-40-e107680-s003.zip › Movie EV3.docx]

**Movie EV3**

Representative intravital imaging videos taken from the same tumor at lower (40 μm) and higher (20 μm) magnification of MDA-MB-231^CFP;Cre;HA-RASSF1C^/T47D^DsRed^ tumors where eGFP^+^ recombined reporter cells adopt amoeboid mode of motility.
