# Supplementary figures and images for: RASSF1C oncogene elicits amoeboid invasion, cancer stemness, and extracellular vesicle release via a SRC/Rho axis
Source: EMBO J. 2021 Sep 17;40(20):e107680. doi: 10.15252/embj.2021107680 (PMC8521318; doi:10.15252/embj.2021107680)

Appendix Figure S2

C

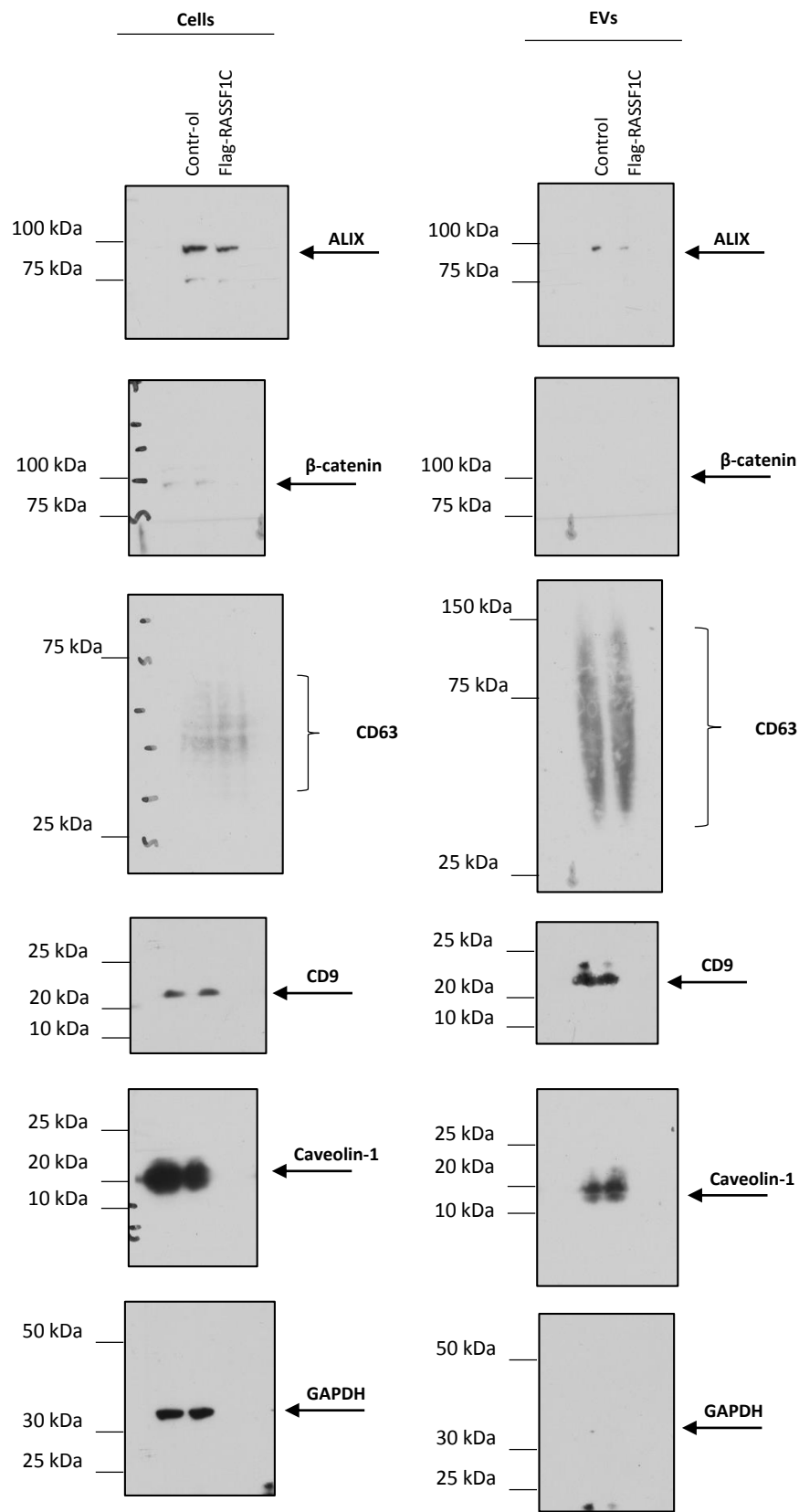

Supplement: Supplementary file 7 — Source Data for Expanded View and Appendix [file EMBJ-40-e107680-s001.zip › Source Data_Appendix Fig S2.pdf]

A

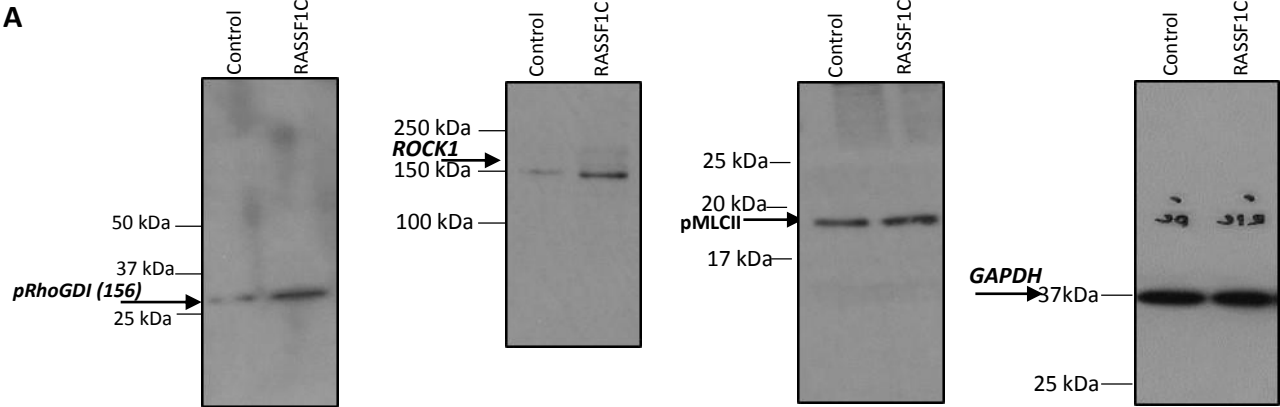

MCF7

B

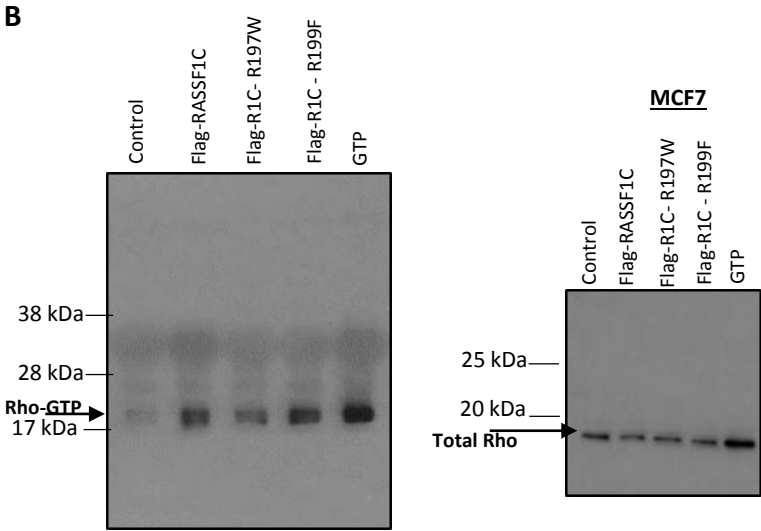

Supplement: Supplementary file 7 — Source Data for Expanded View and Appendix [file EMBJ-40-e107680-s001.zip › Source Data_Figure EV2.pdf]

Expanded View 3

A

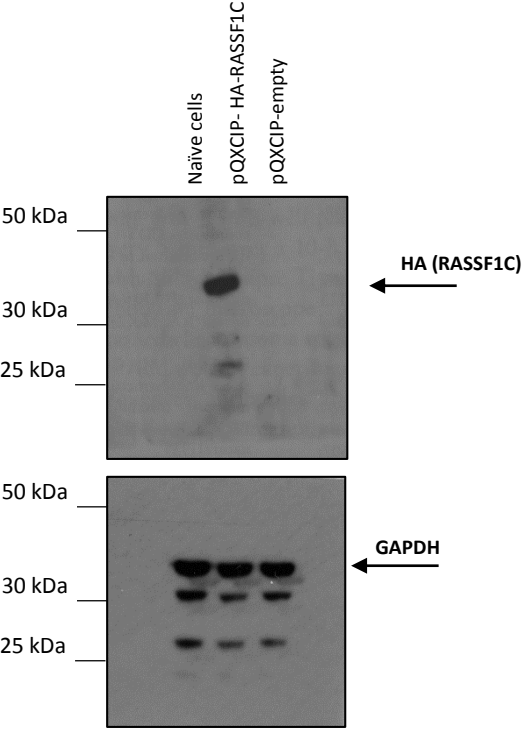

Supplement: Supplementary file 7 — Source Data for Expanded View and Appendix [file EMBJ-40-e107680-s001.zip › Source Data_Figure EV3.pdf]

Figure 1

e

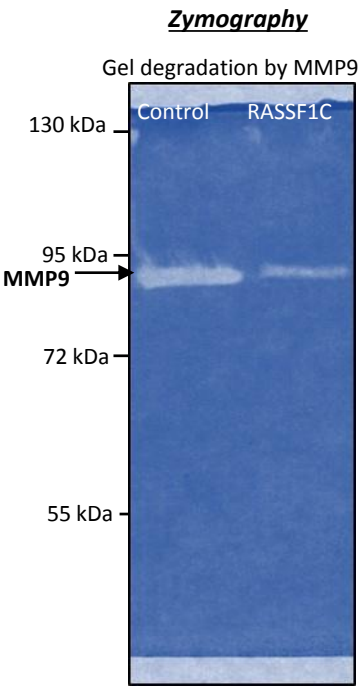

Supplement: Supplementary file 8 — Source Data for Figure 1 [file EMBJ-40-e107680-s010.pdf]

**Figure 2**

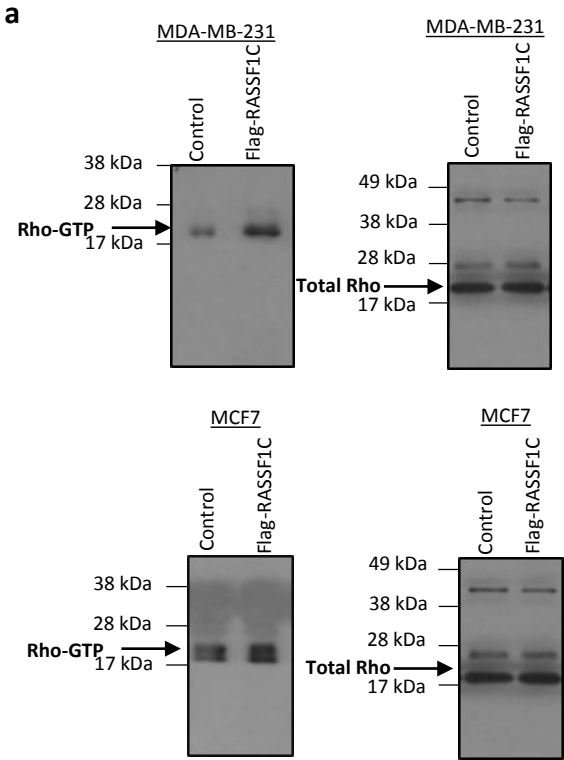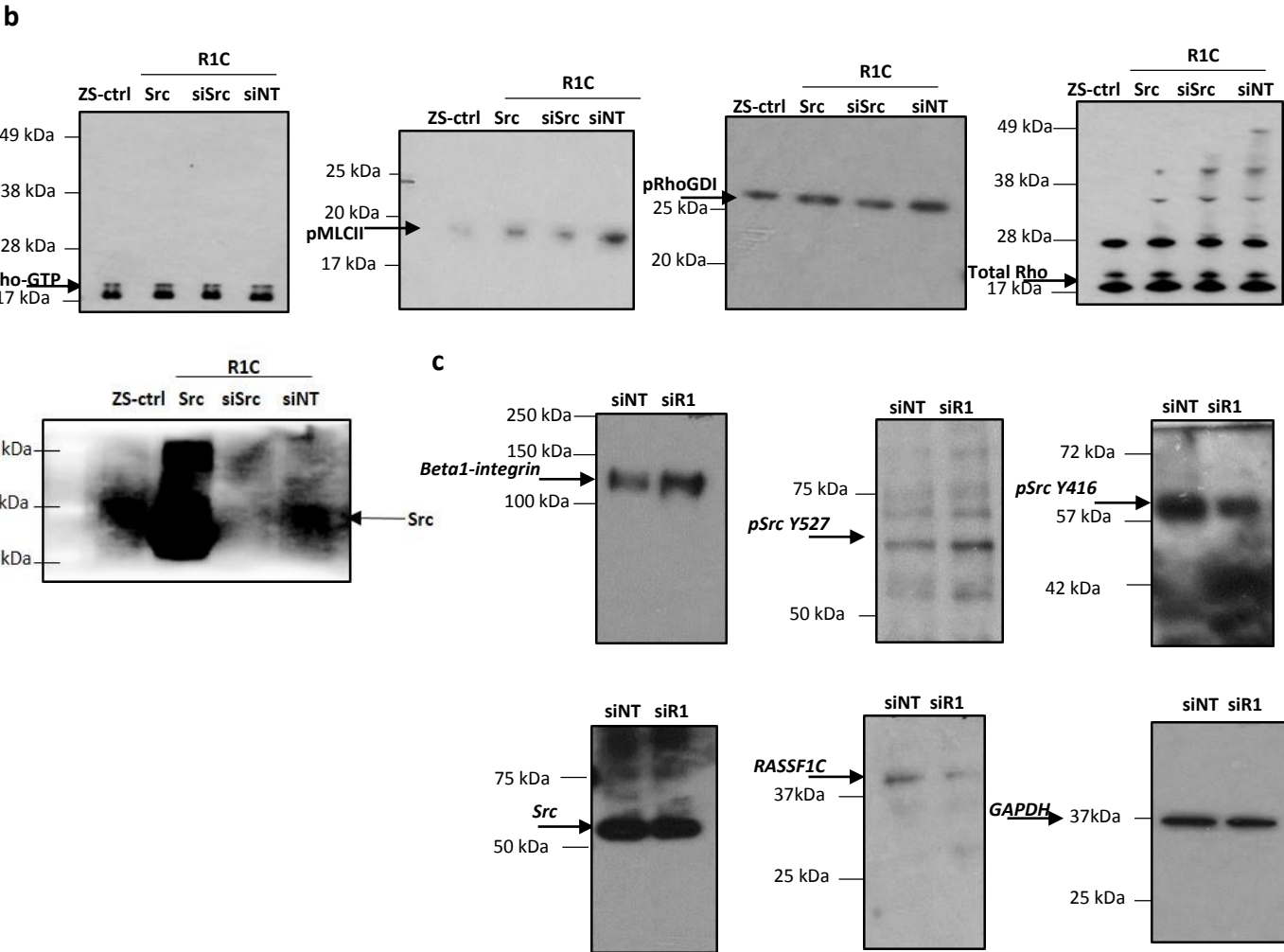

Figure 2

d

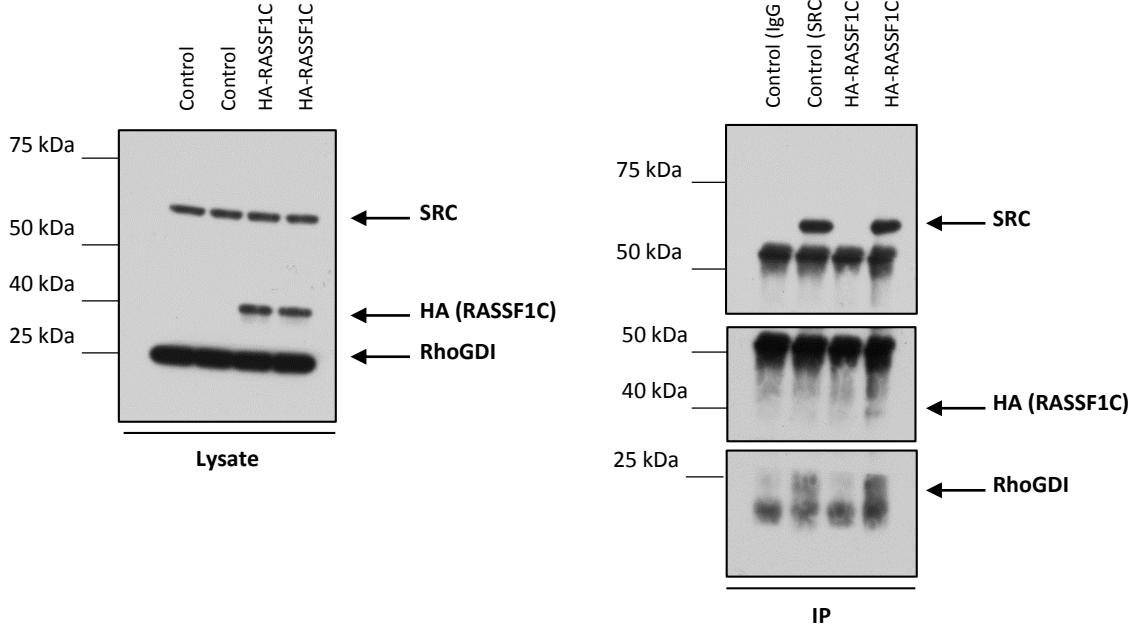

f

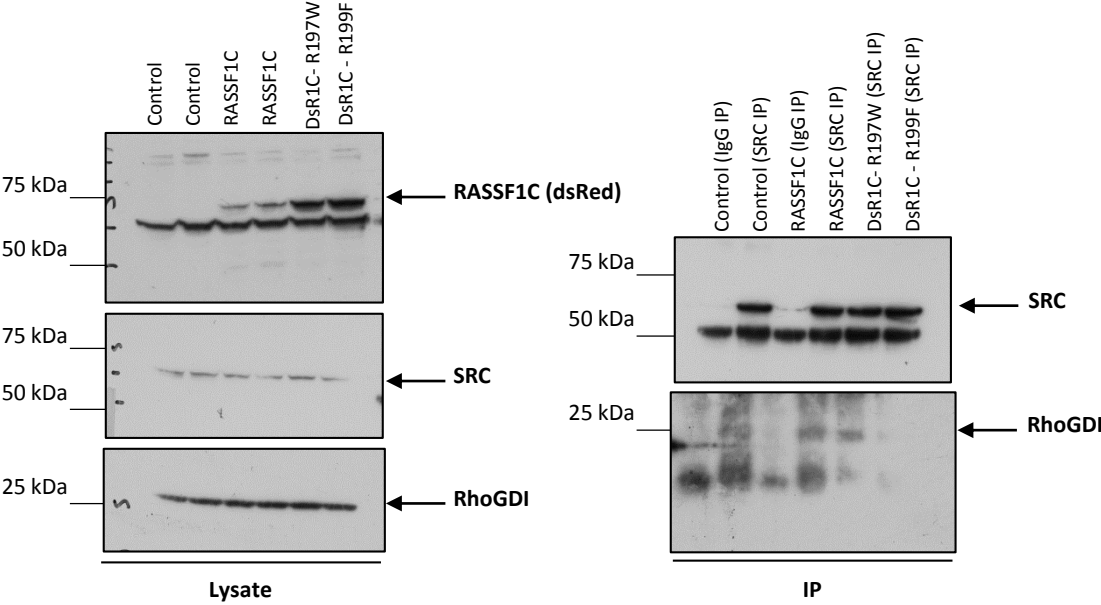

g

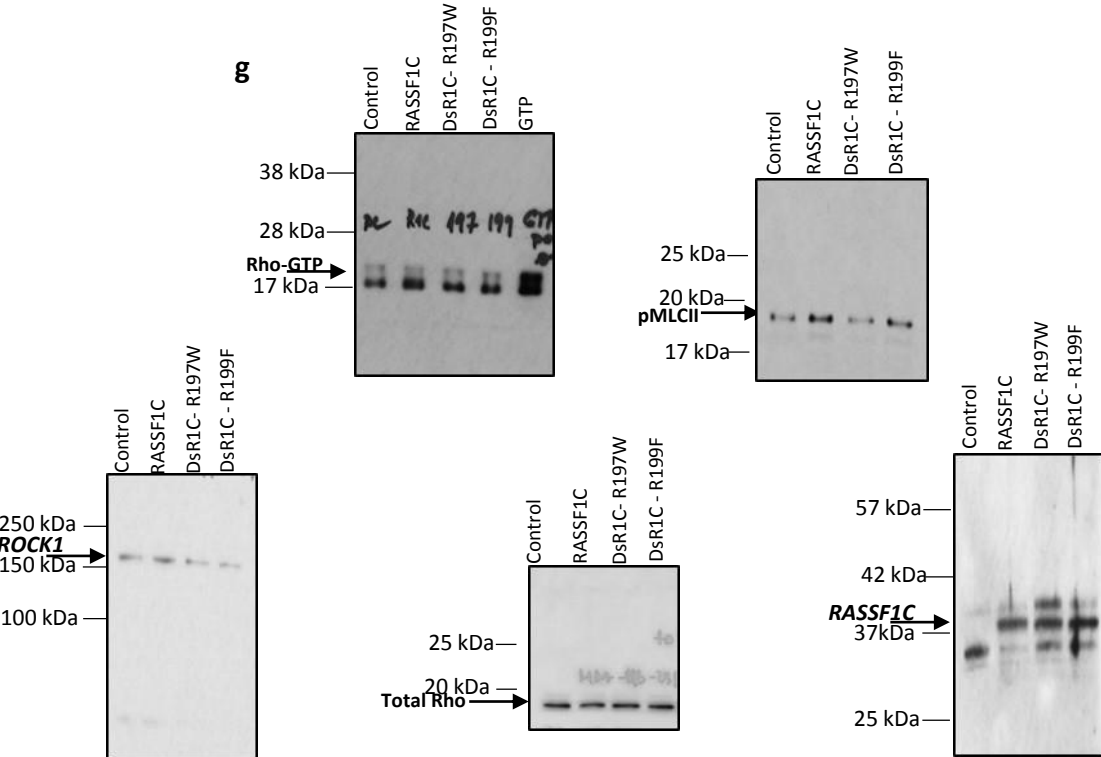

Supplement: Supplementary file 9 — Source Data for Figure 2 [file EMBJ-40-e107680-s005.pdf]

Figure 3

C

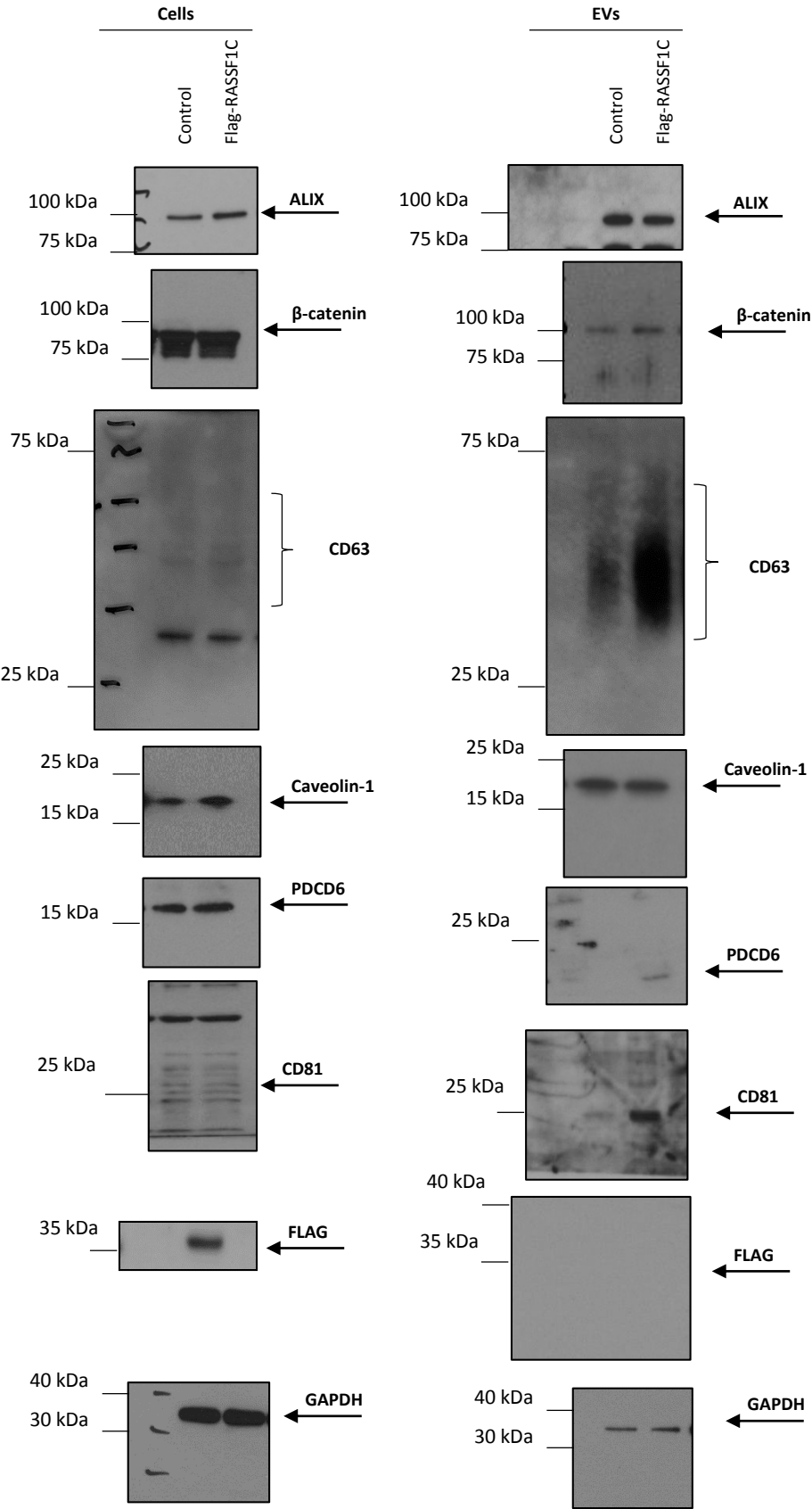

Supplement: Supplementary file 10 — Source Data for Figure 3 [file EMBJ-40-e107680-s004.pdf]

Figure 4

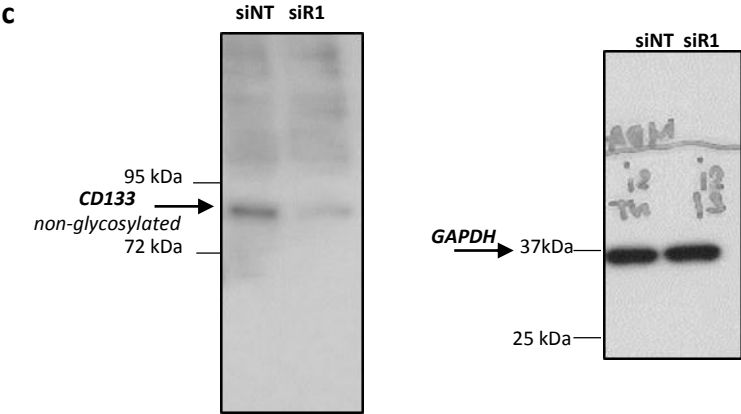

Supplement: Supplementary file 11 — Source Data for Figure 4 [file EMBJ-40-e107680-s002.pdf]
