# Supplementary material for: RASSF1C oncogene elicits amoeboid invasion, cancer stemness, and extracellular vesicle release via a SRC/Rho axis
Source: EMBO J. 2021 Sep 17;40(20):e107680. doi: 10.15252/embj.2021107680 (PMC8521318; doi:10.15252/embj.2021107680)
Supplement: Supplementary file 12 [file EMBJ-40-e107680-s007.pdf]

THE EMBO JOURNAL

# RASSF1C oncogene elicits amoeboid invasion, cancer stemness and extracellular vesicle release via a SRC/Rho axis

Maria Laura Tognoli, Nikola Vlahov, Sander Steenbeek, Anna M. Grawenda, Michael Eyres, David Cano-Rodriguez, Simon Scrace, Christiana Kartsonaki, Alex von Kriegsheim, Eduard Willms, Matthew J. Wood, Marianne G. Rots, Jacco van Rheenen, Eric O'Neill and Daniela Pankova **DOI: 10.15252/embj.2021107680**

*Corresponding authors: Daniela Pankova (daniela.pankova@gmail.com) , Eric O'Neill (eric.oneill@oncology.ox.ac.uk)*



## Review Timeline:

| | |
|---|---|
| Submission Date: | 8th Jan 21 |
| Editorial Decision: | 18th Jan 21 |
| Author Correspondence: | 4th Feb 21 |
| Editor Correspondence: | 5th Feb 21 |
| Editorial Decision: | 23rd Feb 21 |
| Revision Received: | 21st May 21 |
| Editorial Decision: | 25th Jun 21 |
| Revision Received: | 22nd Jul 21 |
| Accepted: | 27th Jul 21 |

*Editor: Daniel Klimmeck*


## Transaction Report:

(Note: With the exception of the correction of typographical or spelling errors that could be a source of ambiguity, letters and reports are not edited. Depending on transfer agreements, referee reports obtained elsewhere may or may not be included in this compilation. Referee reports are anonymous unless the Referee chooses to sign their reports.)

## 1st Editorial Decision 18th Jan 2021

Dear Dr Pankova,

Thank you for submitting your manuscript (EMBOJ-2021-107680-T) to The EMBO Journal. Please accept my apologies for getting back to you with delay due to detailed discussions in the editorial team. I have now read your manuscript carefully and discussed it with my colleagues, and I regret to say that we cannot offer publication in The EMBO Journal.

We appreciate your results on a role for RASSF1c in induction of amoeboid breast cancer cell invasion independently of RASSF1a loss, and a RASSF1c-Src signaling axis for amoeboid transitions, cancer stemness and EV secretion in tumor plasticity. We realise that these results will as such be of interest to the field. However, we also noted that RASS1C was per se shown to promote invasive spread and support Src in adhesion loss. Also, Src was reported to inhibit Rho, and amoeboid migration linked to cancer stemness. Thus, I am afraid we concluded that, despite its notable aspects, your manuscript does not provide the sufficiently striking conceptual advance and functional insights we have to ask for publication at The EMBO Journal. I am therefore sorry to say that we have decided not to send it out for peer-review.

I regret to have to disappoint you on this occasion.

Kind regards,

Daniel Klimmeck

Daniel Klimmeck, PhD
Senior Editor
The EMBO Journal

** As a service to authors, EMBO Press provides authors with the possibility to transfer a manuscript that one journal cannot offer to publish to another EMBO publication or the open access journal Life Science Alliance launched in partnership between EMBO Press, Rockefeller University Press and Cold Spring Harbor Laboratory Press. The full manuscript and if applicable, reviewers' reports, are automatically sent to the receiving journal to allow for fast handling and a prompt decision on your manuscript. For more details of this service, and to transfer your

manuscript please click on Link Not Available.**

Please do not share this URL as it will give anyone who clicks it access to your account.

Dear Daniel,

Many thanks for considering the submission of myself and Daniela Pankovas (co-corresponding) manuscript to EMBO J. We appreciate your comments, but I do feel that the points you raise for not sending out for review could be clarified by a quick discussion.
While the story is built on previous discoveries by us and others, the story we present is entirely novel. All research articles refer to previous work in order to lay foundations for the experimental design and approach which they go on the describe. In this case we are presenting a novel, clinically relevant, driver of ameboid motility that has not been previously described. This is important biologically and clinically, moreover we explain and connect previous individual anecdotal observations into a meaningful explanation for why metastasis spreads this mode of invasive phenotype.

As an editor, I do respect the position you are in and the responsibility to ensure novelty for the journal. I do not make a habit of questioning editorial decisions but would urge you to reconsider in this case. I would be happy to outline our reasoning in greater detail.

Regards
Eric

Dear Daniela, dear Eric,

Thank you for following-up on our decision and calling in to re-emphasize the value of your findings for the field.

We have carefully re-considered your points in the team, and while we have to weigh in that there is precedence for the signalling links reported, we understand the point that joint, cooperative RAssf1c-Src-Rho signaling might not be that obvious. Also, putting this together in the context of in vivo amoeboid motility should have merit.

We have thus concluded to in this case reverse our initial decision, considering this now as a threshold send out. We will have the work evaluated by referees and let you know as soon as we hear back from them.

Best regards,

Daniel

## 1st Revision - Editorial Decision 23rd Feb 2021

Dear Daniela, dear Eric,

Thank you again for the submission of your manuscript (EMBOJ-2020-107680) to The EMBO Journal. Your study has been sent to three reviewers for evaluation, and we have received reports from all of them, which I enclose below.

As you will see, the referees acknowledge the potential interest and novelty of your results, although they also express a number of issues that will have to be conclusively addressed before they can be supportive of publication of your manuscript in The EMBO Journal. In more detail, referee #1 raises concerns on the robustness of the results and asks you to add additional experimentation in order to consolidate the findings. Reviewer #3 agrees in that additional replicates are needed to corroborate your results. This referee also asks you to evaluate a role for pMLCII in the RASSF1C-Src-Rho signalling axis. Referee #2 states that more work is required to support a Src-dependent role of Rassf1c in EV secretion. In addition, the reviewers raise a number of points related to data annotation and statistics as well as discussion of the findings in light of recent literature, which would need to be conclusively addressed to achieve the level of robustness and clarity needed for The EMBO Journal.

I judge the comments of the referees to be generally reasonable and given their overall interest, we are in principle happy to invite you to revise your manuscript experimentally to address the referees' comments. Please keep in mind thought that given the overall subtle effects, the statistics-quantification issues have to be solved to the full satisfaction of the reviewers in order to move on with this towards publication.

Please let me know any time if you have additional questions or need further input on the referee comments.

Please see below for additional instructions for preparing your revised manuscript.

Thank you for the opportunity to consider your work for publication. I look forward to your revision.

Kind regards,

Daniel

Daniel Klimmeck, PhD
Senior Editor
The EMBO Journal

Instructions for preparing your revised manuscript:

Please make sure you upload a letter of response to the referees' comments together with the

revised manuscript.

Please also check that the title and abstract of the manuscript are brief, yet explicit, even to non-specialists.

When assembling figures, please refer to our figure preparation guideline in order to ensure proper formatting and readability in print as well as on screen:
https://bit.ly/EMBOPressFigurePreparationGuideline

IMPORTANT: When you send the revision we will require
- a point-by-point response to the referees' comments, with a detailed description of the changes made (as a word file).
- a word file of the manuscript text.
- individual production quality figure files (one file per figure)
- a complete author checklist, which you can download from our author guidelines (https://www.embopress.org/page/journal/14602075/authorguide).
- Expanded View files (replacing Supplementary Information)
Please see out instructions to authors
https://www.embopress.org/page/journal/14602075/authorguide#expandedview

Please remember: Digital image enhancement is acceptable practice, as long as it accurately represents the original data and conforms to community standards. If a figure has been subjected to significant electronic manipulation, this must be noted in the figure legend or in the 'Materials and Methods' section. The editors reserve the right to request original versions of figures and the original images that were used to assemble the figure.

Further information is available in our Guide For Authors:
https://www.embopress.org/page/journal/14602075/authorguide

The revision must be submitted online within 90 days; please click on the link below to submit the revision online before 24th May 2021.

Link Not Available

------------------------------------------------

Referee #1:

In this manuscript the authors describe a mechanisms how the oncogene RASSF1C promotes mesenchymal-to-amoeboid transition. Interestingly, this process appears to promote breast cancer cell stemness associated with a more invasive phenotype as well as the release of 'oncosomes'. They further describe a RASSF1C promotor methylation that acts as a biomarker for better prognosis in patients with early stage disease. The work is of significant importance and is by and large well conducted. I have listed some important points below that should be addressed to strengthen the conclusions.

Comments:

RASSF1C oncogene elicits amoeboid invasion, cancer stemness and invasive EVs via a novel SRC/Rho axis
p. 4 - Fig 1a and S1c: how do cells behave long-term? How do they look after 24-48 h? Do they round up and die after RASSF1C overexpression?
p. 5 - Fig. 1b + S1e: even though statistically significant, the data are not very convincing for the epithelial cell lines MCF7 and H1299 (an increase in amoeboid morphology from already 80% of cells in untreated cells to 81% in RASSF1C overexpressing cells?).
p. 5 - Fig S1f: the authors write: "We reasoned that expression of RASSF1C is responsible for epithelial-amoeboid (EAT, in MCF7 and H1299 cell lines) and mesenchymal-amoeboid transition (MAT, in MDA-MB-231 cell line), and for the switch from elongated into rounded morphology induced in 3D collagen matrix." No data are shown for EAT of MCF7 in 3D collagen matrix. The hanging drop experiment should be repeated for this cell line, otherwise it seems difficult to claim a EAT phenotype for MCF7 cells after RASSF1C overexpression.
p. 5 - Fig 1d: the bar graph is labelled with "siRASSF1". Was the siRNA used for this experiment specific for the isoform RASSF1C as written in the text? If so, please specify in the graph. (same for Fig. 4c, S4A).

p. 7 - Fig 2 b:
• a WB control to show efficient SRC knockdown upon siSRC treatment is missing
• The authors write "Transient knock-down of SRC by siRNA showed a decrease in Rho activity (measured as lower expression levels of Rho-GTP in the pull-down assay)."
o Which of the two bands corresponds to Rho-GTP?
o Difference/decrease in Rho activity is not very convincing
• Authors write: "SRC over-expression resulted in increased inhibitory phosphorylation of RhoGDI (pY156), a Rho GTPase inhibitor, and elevated levels of pMLCII (pS19) (Fig. 2b)"
o Was a Quantification of RhoGDI by Western analysis done?
o How do the authors explain the increase in pMLCII levels in siNT-treated cells (without SRC overexpression) that seems to be even higher than after SRC overexpression? Could the effect be attributed to RASSF1C alone?

p.8 - Fig 2g: The Western blot labelling not clear to me. What ratios are given in the figure?

p. 8 - Fig 2h: I don´t understand the labelling of the y-axis. % of total number of cells in that field of view? How can it be more than 100% of total (e.g. control, mesenchymal is 140% total). Also, shouldn´t the two bars of one treatment group sum up to appr. 100% if they display one of the two given morphologies as seen in Fig 1b, 1d etc.?

p. 9: "...whereas a RhoA dominant negative derivative (RhoA-DN) had an opposite effect (Fig. 3d)." decrease visible in Fig 3d, however, no statistics shown.

p. 10 - Fig 3e:
• How were the statistics performed? Tested for each individual data point or given p-value only for end-point data?
• It is difficult to interpret the migration data above 24h since cells could already start proliferating, which will also be detected by the xCELLigence device.
• It looks like control cells migrate at the same speed/rate (=slope of the graph) like the RASSF1C transfected cells, they just start migration a bit later (curve shifted to the right).

p. 10 -Fig S3H: The CD63 Western blot does not look like a specific antibody-stained band but more like a smear.

p. 10 - Fig 3f+g:

"In our setup, the RASSF1C large and medium EVs did not confer higher invasive ability to recipient cells, compared to the corresponding control fractions."
"The data so far suggest that RASSF1C-derived small EVs, and no other fractions, transfer the invasive potential to naïve cells."
But compared to EV´s from untreated cells they all (also large and medium EV´s) conferred invasive potential.

"Taken together, the data show that RASSF1C promotes EV functional transfer and that both RASSF1C and RhoA expression promote the release of pro-migratory and pro-invasive EVs."
Fig 3f: no significant increase after addition of RhoA-EV´s when comparing to its control group (control plasmid) instead of comparing to untreated control

Minor points:

General Western Blots: no markers/sizes given in the WB data
Fig 1a: scale bars missing
p. 8 - Fig S2d: membrane blebs don´t look very prominent to me...? Possible to have video footage or better image?

Figure legend to Suppl. Fig 2d: "Cells were stained with F-actin (red)...". This should probably say "...were stained with phalloidin for F-actin.:"

Referee #2:

The manuscript by Tognoli & Vlahov etc is well written and describes a new role for RASSF1C in breast cancer progression by describing a role in upregulating Rho/rock signalling axis in cancer - leading to stem like phenotypes and importantly revealing an EV based role for the transfer of a more invasion, migratory phenotype (in vitro and in vivo by intravital imaging)....were RASSF1C role via SRC can regulate these phenomenon via rock/rho signalling axis.

The experiment are well designed with caveats addressed in the paper and discussed....
Moreover appropriate control experiment are evident over activating/reducing expression or activity for RhoA CA/DN
Links to the clinical relevance are clearly show via TCGA and patient data etc and also nice work is presented via RASSF1C epigenetic control vi dCas9-VP64 in this study should be applauded.
Lastly the small versus medium/large EVs speaks to novelty for this MS..I would like the authors to talk to recent work on ROCK/KD effects in breast cancer Pmyt on secretome conditional media work Involving Creld2 and discuss how this work aligns to this.- pro tumourogenic .... The author touch upon this for manipulation of the tumour microenvironment but more should be discussed re the role of targeting ROCK in this area.

-Since the author describe the role for RASSF1C in SRC based control of Rock/Rho signalling via RhoGDI -can the authors perform experimets to inhibit Src either via molecular manipulation or via

commonly used drugs to target Src in principle? This can be done for in vitro setting and can also be assessed in vivo to assess if Src inhibition here would reduce EV effects on surrounding cell phenotypes and migratory potential etc..this would speak to the pathways role in this disease and its potential clinical targeting. Similar as discussed above Rock targeting can be discussed in this context as a drug target.

Referee #3:

In this manuscript, the ability of the RASSF1C oncogene to affect breast cancer cell morphology, invasive behaviours and stemness has been examined. RASSF1C is expressed as a result of an epigenetic switch that represses its homologue RASSF1A, which has previously been characterized as a tumour suppressor. The expression of RASSF1C is reported to activate the SRC tyrosine kinase, leading to phosphorylation and inhibition of Rho-GDI protein, resulting in increased Rho activity. The increase in Rho activity promotes myosin light chain phosphorylation, driving cell contractility and morphological transitions from mesenchymal or eplithelial to rounded or amoeboid morphologies. In parallel, these morphological changes are associated with changes in the expression of stem cell markers, including CD133, ALDH1 and NANOG, suggesting that the morphological transformation is a driver of cancer cell stemness. The altered morphology also promotes the generation and release of extracellular vesicles that promote tumour cell migration. In vivo, cells expressing RASSF1C were more motile, and adopted a rounded mode of motility. Experiments designed to assess the transfer of material to recipient cells via extracellular vesicles revealed that RASSF1C increased this horizontal transfer, both in vitro and in vivo, likely contributing to increased invasion of the recipient cells. Analysis of gene methylation patterns revealed a reciprocal relationship between RASSF1A and RASSF1C, consistent with respective tumour-suppressive and oncogenic roles in breast cancer patients.

Overall, the study is interesting and logical. In general, the experiments are well designed and appropriate. Major issues include a lack of experimental replicates, quantification and statistical analysis of some experiments, particularly western blot experiments that indicate subtle responses. The relatively small changes observed are not the problem, many biological processes are governed by small changes in signalling. However, given the inherent variability in some of the assays that have been used, it is important to provide data showing that the observations are reproducible and robust.

Points to be considered
1. Introduction - Why state that Rho/ROCK signalling promotes MLCK mediated MLCII phosphorylation? The evidence that ROCK kinases directly phosphorylate MLCII was reported several decades ago, and yet I can not think of a convincing mechanism for ROCK promoting MLCK activity. Unless you are referring to a fairly old idea that MLCK is the only MLC kinase, and that ROCK operates exclusively as an inhibitor of the MLCII phosphatase via MYPT1 phosphorylation?
2. Figures 1b and 1d - the morphologies should be defined as rounded and elongated, amoeboid and mesenchymal refer to migration modes, as this assay is based purely on cell shape.
3. Figure 1c - does the overexpression of RASSF1C increase pMLCII levels? Or does it just cause the staining to be re-localized? This would be easily addressed by western blotting. Is the increase in pMLCII levels sensitive to Y27632 or C3?
4. Previous studies (including by the current author's studies from 2015) have shown that overexpressed RASSF1C is localized at microtubules and at the nuclear envelope. Is RASSF1C localized on microtubules in Figure 1c and 2i?. If so, does the change in cell morphology have

anything to do with RASSF1C microtubular localization?
5. Figure 1d - does the knockdown of RASSF1C decrease pMLCII levels or change its localization? Does it change the distribution of F-actin from the cortical cytoskeleton to stress fibres? Western blots (or qPCR) should be included for Figure 1d to show the amount of RASSF1C depletion. There is a similar absence of controls for the extent of knockdown achieved in other figures that should be rectified.
6. Figure 1e - how was it determined that this activity is MMP9? Was a western blot done in parallel? Based on the molecular weight of the band? If so, it would be important to show the MW markers. How reproducible is this observation? Is it statistically significant?
7. Figure 1f - given that MMPs can alter cell behaviours by acting as autocrine factors that affect cell signalling pathways, it is not a given that the hydrogel experiment proves that the effect of RASSF1C is MMP independent. In fact, given the result in the left panel, it's not even clear why this experiment was done. But it would be more robust if it were shown that GM6001 did not affect invasion into hydrogel, in agreement with the lack of effect on Matrigel invasion.
8. Figure 1g - it would be more obvious if the labelling indicated Y27632 in the graph to be consistent with the text. C3 hasn't been labelled as RHOi, so why not just label them with the way that the experiment was actually done. Given that there are many more ROCK inhibitors than just Y27632, why not be explicit about the experiment?
9. Figure 1g - is the effect of Y27632 and C3 on the invasion induced by RASSF1C actually significant?
10. Figure 1j - the cell migration speeds are indicated, the text reports that the cells expressing RASSF1C move via "more amoeboid, rounded morphology". This data is actually important and should be moved from the supplementary data to the primary figures.
11. Figure 2a - Which Rho protein is this? RhoA? Given the variability of the pull-down assays, this should be repeated, quantified and statistically tested.
12. Figure 2b - Are the levels of active Rho reproducible and significant? Are the differences in RhoGDI phosphorylation reproducible and significant? By eye, it's not even apparent that there are differences, which reinforces the importance of replication and quantification.
13. Figure 2b - the levels of pMLCII between +/- RASSF1C are apparent, and this actually addresses point 3 I made above. But the effect of either SRC over-expression or siRNA mediated knockdown appear to be the same, both result in decreased pMLCII. According to the model of this paper, the decrease in pMLCII when SRC is knockdown is logical, but why would SRC over-expression decrease pMLCII? This again reinforces the need to replicate these experiments, quantify and statistically evaluate.
14. Figure 2c - replicates, quantification, statistical evaluation are needed.
15. Figure 2d - This experiment indicates that SRC and RhoGDI are constitutively associated? And then if HA-tagged RASSF1C is included, it can be found in the complex by blotting for the HA tag. But it doesn't actually address whether RASSF1C is needed for, or promotes, the SRC-RhoGDI interaction. Can endogenous RASSF1C be found in this complex? Does RASSF1C knockdown reduce the SRC-RhoGDI interaction. Also, please label what has been IPed on the figure, the text didn't actually state this and only the figure legend indicated what antibody has been used.
16. Figure 2f - label the IP please. What happened in the RhoGDI blot in the control + SRC lane? Wouldn't the over-expression of RASSF1C alone be expected to increase the association of SRC with RhoGDI compared to the SRC alone according to the model? That would be predicted if the R197W and R199F expression resulted in less SRC-RhoGDI interaction. Unless the idea is that these point mutations antagonize the SRC-RhoGDI interaction? Also, is there actually less RhoGDI in the R197W lane compared to the wild-type RASSF1C? This again reinforces the need for replicates when there are such subtle differences.
17. Figure 2g - label the top panel please. It seems like Figures 2g and Supplemental Figure 2a are revealing a new mechanisms of action of RASSF1C, that it regulates ROCK1 protein levels. Why is

this not part of the central hypothesis? How reproducible is this observation? Is it post-translational or transcriptional? The subtle effects on pMLCII should be corroborated through replicates, quantification and statistical evaluation.
18. Figure 2i - what are the proportions of rounded vs elongated cells for each condition? Similar to what was reported in Figure 2d.
19. Text - section headed "RASSF1C amoeboid cells release pro-invasive extracellular vesicles". In the first sentence, it is stated that RASSF1C leads to the generation of protrusive forces, but formally this has not been shown. Please revise to agree with what the data actually shows.
20. Figure 3a - data normalization is fine, but it should give an indication of the variability in the control condition as well. Otherwise, the statistical test is biased because of the artificial absence of variability in the control.
21. Figure 3e - it should be described that this experiment is chemotaxis experiment akin to a Boyden chamber rather than migration across a 2D surface.
22. Figure 4j - can the authors speculate why SOX2 was induced in EV recipient cells, but not in the RASSF1C over-expressing cells in Figure 4g? If SOX2 isn't induced, is RASSF1C really inducing stemness, or something that is "stem-like"?
23. This sentence from MS page 14 is confusing "In line with our data supporting a pro-oncogenic role for RASSF1C, deceased patients had less median methylation of RASSF1-2.." Does this mean lower median methylation?

Minor points
1. Reviewing papers is easier if there are page and line numbers. Given the length and complexity of this manuscript, reviewing it would have been considerably easier if they had been included. I recommend it for future submissions
2. The number of times that abbreviations (e.g. EMT, MAT, etc.) are defined is excessive, the first occurrence is sufficient.
3. New paragraphs would be more obvious if the first line were indented, or if there was a line between it and the previous paragraph.
4. Consider making the colours in the graphs friendlier for people with red-green colour blindness.

*Referee#1:*

*In this manuscript the authors describe a mechanisms how the oncogene RASSF1C promotes mesenchymal-to-amoeboid transition. Interestingly, this process appears to promote breast cancer cell stemness associated with a more invasive phenotype as well as the release of 'oncosomes'. They further describe a RASSF1C promotor methylation that acts as a biomarker for better prognosis in patients with early stage disease. The work is of significant importance and is by and large well conducted. I have listed some important points below that should be addressed to strengthen the conclusions.*

*Comments:*
*RASSF1C oncogene elicits amoeboid invasion, cancer stemness and invasive EVs via a novel SRC/Rho axis*
*p. 4 - Fig 1a and S1c: how do cells behave long-term? How do they look after 24-48 h? Do they round up and die after RASSF1C overexpression?*

We thank the reviewer for asking us to clarify this important point. We hereby provide data of MCF7 cells transiently transfected with Control or RASSF1C constructs (ZsGreen or Flag tagged) and stained 72 h after transfection with a proliferation marker (Ki-67), a mitotic marker (phospho-H3), and an apoptotic marker (FITC-VAD). The data show that rounded cells are not undergoing mitosis ($pH3^{-ve}$) or apoptosis (caspase $activity^{-ve}$), physiological states associated with rounded morphology, but are $Ki67^{+ve}$ indicating that they are not quiescent as has been observed for amoeboid glial cells during development (*Zusso, M. et al. Regulation of postnatal forebrain amoeboid microglial cell proliferation and development by the transcription factor Runx1. J Neurosci* **32**, 11285-11298 (2012)).

MCF7

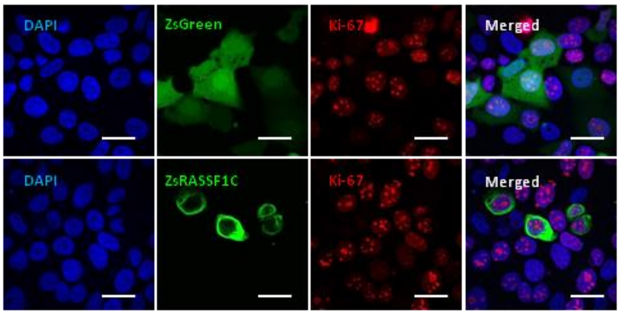


MCF7

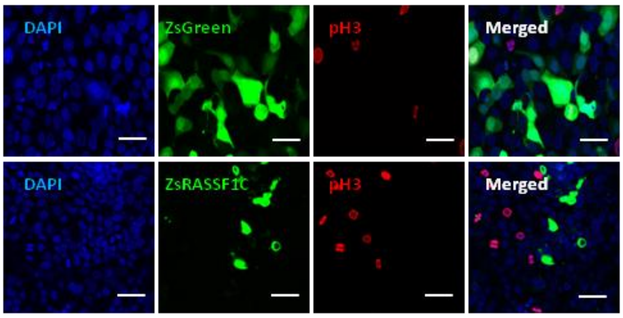


MCF7

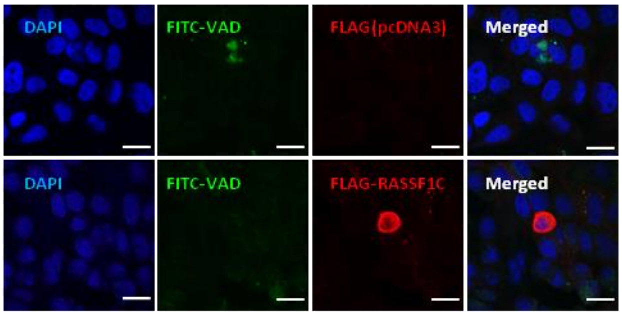


Furthermore, we provide data of MDA-MB-231 cells stably expressing a ZsGreen-Control or a ZsGreen-RASSF1C construct that were FACS sorted for green$^{high}$ expression, re-seeded and kept in culture for further 7 days to monitor cell growth. In this setup we could observe that cells remained viable (alive) but failed to proliferate up to 7 days post sorting.

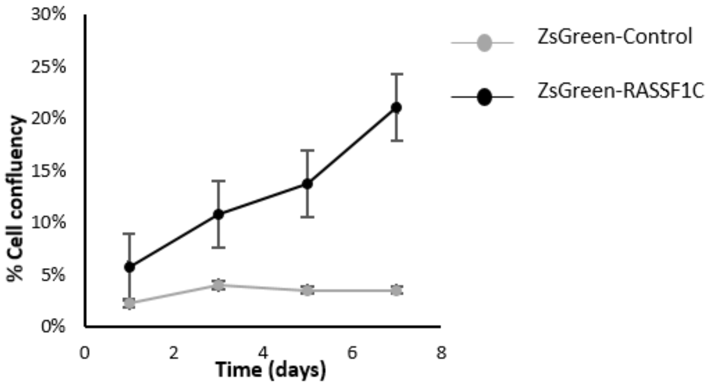


The above data has now been included and discussed in the manuscript as Figure EV1C-D.

*p. 5 - Fig. 1b + S1e: even though statistically significant, the data are not very convincing for the*

*epithelial cell lines MCF7 and H1299 (an increase in amoeboid morphology from already 80% of cells in untreated cells to 81% in RASSF1C overexpressing cells?).*

We apologise to the reviewer for not having clearly stated it in the manuscript, but our data show that RASSF1C overexpression in epithelial MCF7 caused 5% morphology shift (from 80% of epithelial-polygonal morphology to 85% amoeboid-rounded morphology) and in H1299 7% morphology shift (from 81% epithelial-polygonal shape to 88% amoeboid-rounded morphology). Although a minor shift in single cell morphology in 3D collagen matrix, these data were included to support mechanistic EAT (epithelial-amoeboid transition) and that rounded morphology driven by RASSF1C-Rho activation can also occur in epithelial MCF7 cells (Fig 2A, EV2A). This information has also been included in the text (lines 109-110).

*p. 5 - Fig S1f: the authors write: "We reasoned that expression of RASSF1C is responsible for epithelial-amoeboid (EAT, in MCF7 and H1299 cell lines) and mesenchymal-amoeboid transition (MAT, in MDA-MB-231 cell line), and for the switch from elongated into rounded morphology induced in 3D collagen matrix." No data are shown for EAT of MCF7 in 3D collagen matrix. The hanging drop experiment should be repeated for this cell line, otherwise it seems difficult to claim a EAT phenotype for MCF7 cells after RASSF1C overexpression.*

We again apologise to the reviewer for not making this point clear. While we did show data for EAT (single cell morphology assays in 3D collagen) for MCF7 in Fig 1B and EV1F, we did not provide spheroids morphology for MCF7 and H1299 as with their epithelial-polygonal cell shape it will be technically very difficult to distinguish different cell morphologies in 3D collagen by microscopy Z-stacks. Instead, we provided single cell morphology assay in 3D collagen (as an alternative), wherein cells can better adapt their plasticity to the environment and it is technically possible to recognize the transition from polygonal (epithelial) to rounded shapes.

*p. 5 - Fig 1d: the bar graph is labelled with "siRASSF1". Was the siRNA used for this experiment specific for the isoform RASSF1C as written in the text? If so, please specify in the graph. (same for Fig. 4c, S4A).*

We thank the reviewer for highlighting lack of information regarding the siRNA used in the mentioned experiments. The siRNA used in these experiments targets exons common to all *RASSF1* isoforms (exon 3) as previously published (*Vlahov, N. et al. Alternate RASSF1 Transcripts Control SRC Activity, E-Cadherin Contacts, and YAP-Mediated Invasion. Curr Biol 25, 3019-3034 (2015)).* In the present manuscript, however, we show that RASSF1C is the predominant isoform expressed and the RASSF1-1α promoter derived isoforms are not expressed (see data below, which corresponds to Fig EV1B, middle panel). Therefore, the use of this siRNA (siRASSF1) reliably targets the RASSF1C isoform and RASSF1A is not transcribed.

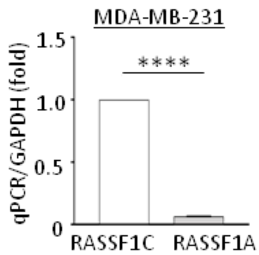


This is supported by a wide body of evidence that RASSF1-1α promoter is highly methylated in the cell lines used in this study (References from the current manuscript: *Calanca et al. Epigenetics 2019.; Chatzifrangkeskou et al. EMBO J 2019; Montenegro et al. PLoS One 2012).*

*p.7 - Fig.2b:*

*• a WB control to show efficient SRC knockdown upon siSRC treatment is missing*

We apologise to the reviewer for not including a western blot analysis after siRNA-mediated KD of Src. We have now included the missing control below and in Fig 2B.

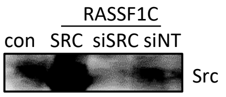


*• The authors write "Transient knock-down of SRC by siRNA showed a decrease in Rho activity (measured as lower expression levels of Rho-GTP in the pull-down assay)."*

*o Which of the two bands corresponds to Rho-GTP?*

We thank the reviewer again for noticing this imprecision. As the appereance of GST affinity-purified Rho-GTP double bands have been documented in previous studies (*Sander et al. 1999. J Cell Biol; Carton et al., Am J of Physiol, 2002*), we believe that both bands represent active Rho.

*o Difference/decrease in Rho activity is not very convincing*

We apologise to the reviewer for not including statistical analysis of Rho activity in Fig 2B, which was analysed by Rho-Pull down assay. This is now corrected and added into the manuscript as a part of EV2B.

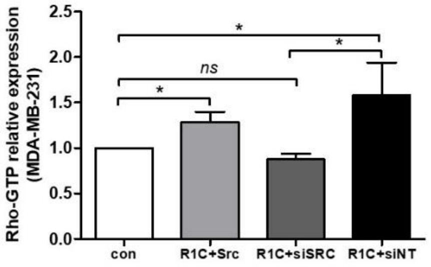


*• Authors write: "SRC over-expression resulted in increased inhibitory phosphorylation of RhoGDI (pY156), a Rho GTPase inhibitor, and elevated levels of pMLCII (pS19) (Fig. 2b)"*

*o Was a Quantification of RhoGDI by Western analysis done?*

We thank the reviewer for raising this point. We have now included a quantification of pMLCII and pRhoGDI western blot analysis as part of EV2B.

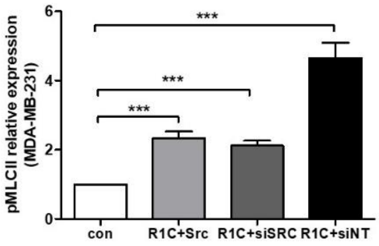


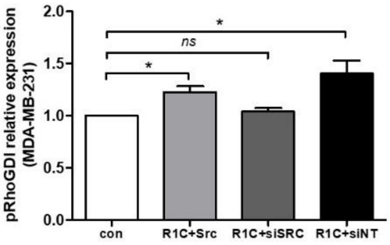


*o How do the authors explain the increase in pMLCII levels in siNT-treated cells (without SRC overexpression) that seems to be even higher than after SRC overexpression? Could the effect be attributed to RASSF1C alone?*

Indeed, we show in Fig EV2C that RASSF1C expression alone leads to increased pMLCII levels (now quantified and included as EV2C). This phenomenon is not surprising, as we have previously demonstrated that RASSF1C is upstream of SRC and leads to its activation (*Vlahov, N. et al. Curr Biol*

*2015*). We therefore postulate in this case that expression of RASSF1C is sufficient to activate SRC and in turn to generate the observed changes, among which is the increase in pMLCII levels.

*p.8 - Fig 2g: The Western blot labelling not clear to me. What ratios are given in the figure?*
We apologise for the confusion here. The labelling in the first lane was indeed missing. We have now amended it to "Rho-GTP (active)" and specified that the ratio is referred to Rho-GTP.

*p. 8 - Fig 2h: I don´t understand the labelling of the y-axis. % of total number of cells in that field of view? How can it be more than 100% of total (e.g. control, mesenchymal is 140% total). Also, shouldn´t the two bars of one treatment group sum up to appr. 100% if they display one of the two given morphologies as seen in Fig 1b, 1d etc.?*
We apologise once again for mislabelling the *y* axis title in this figure. This has now been corrected to "Total number of cells" analysed.

*p. 9: "...whereas a RhoA dominant negative derivative (RhoA-DN) had an opposite effect (Fig. 3d)." decrease visible in Fig 3d, however, no statistics shown.*
We thank the reviewer for raising this point. We have now added statistics also for this condition, compared to control. Despite the decrease in Rho-DN condition, the p-value calculated was not significant.

*p. 10 - Fig 3e:*
*• How were the statistics performed? Tested for each individual data point or given p-value only for end-point data?*
We apologise for the lack of statistical methods. Statistics were performed as a 2-way ANOVA test with Dunnett's multiple comparisons tests and p-value was derived based on comparison of the mean of each treatment condition (RASSF1C or RhoA EVs) to the control one (Control EVs). This information has now been added to the figure legend.

*• It is difficult to interpret the migration data above 24h since cells could already start proliferating, which will also be detected by the xCELLigence device.*
We thank the reviewer for making this point and agree that data representation would be more representative of migration if kept up to 24 h data-point. We therefore made new figures where the last time-point is indeed 24 h and adjusted the statistics accordingly (Fig 3E).

*• It looks like control cells migrate at the same speed/rate (=slope of the graph) like the RASSF1C transfected cells, they just start migration a bit later (curve shifted to the right).*
We thank the reviewer for raising this point and we acknowledge that the differences in slope may not be appreciable from the figure. For this reason we have now selected a window of time where a linear migration phase was observed (5-10 h) and we calculated the slope for each condition in this time-frame. Below are the slope values represented as a ratio over control:

| ***Ratio over control*** | |
|---|---|
| Control | 1 |
| RASSF1C | 1.456587 |
| RhoA | 1.614782 |

The data show that RASSF1C and RhoA cells migrate respectively at a 1.46 and 1.62-fold faster than control cells.

*p. 10 -Fig S3H: The CD63 Western blot does not look like a specific antibody-stained band but more like a smear.*

We thank the reviewer for commenting on the specificity of the CD63 antibody. The CD63 protein has four N-glycosylation sites and can therefore undergo PTM that may result in a smear-like band rather than a defined band at a molecular weight spanning between 25 and 50 KDa. This has been observed by us and others (please see the following publications as references: Van Niel et al., Dev Cell. 2011., *Fig 2A CD63 WB*; Zomer et al., Cell. 2015, *Fig 2E CD63 WB*). The antibody manufacturer page also shows a smear rather than a sharp band (please see enlarged image in: https://www.novusbio.com/products/cd63-antibody-h5c6_nbp2-42225).

*p.10-Fig3f+g:*

*"In our setup, the RASSF1C large and medium EVs did not confer higher invasive ability to recipient cells, compared to the corresponding control fractions."*
*"The data so far suggest that RASSF1C-derived small EVs, and no other fractions, transfer the invasive potential to naïve cells."*
*But compared to EV´s from untreated cells they all (also large and medium EV´s) conferred invasive potential.*
We thank the reviewer for raising this point. Numerous studies have shown that not only small cancer EVs (i.e. in our protocol isolated via a 100.000 *x g* centrifugation step) but also larger EVs (i.e. EVs isolated via lower centrifugation steps) retain invasive potential. With these data we also observe that any EV type (i.e. isolated at any centrifugation step or isolated from control or RASSF1C cells) retain this ability. However, our main focus here was to show that RASSF1C-derived small EVs conferred an advantage to recipient cells compared to their control counterparts, a phenomenon that is less appreciable for the other EV categories we examined. Nevertheless, we would like to take the reviewer's comment into account and, to this end, we amended the text within the results (see below).
*"In our setup, RASSF1C large and medium EVs did not confer* ***significantly*** *higher invasive ability to recipient cells, compared to the corresponding control fractions." (lines 287-289).*
*"The data so far suggest that,* ***despite all EV fractions showing a degree of ability to confer invasive potential,*** *it is the RASSF1C derived small EVs that significantly transferred invasive potential to naïve cells." (lines 297-299).*

*"Taken together, the data show that RASSF1C promotes EV functional transfer and that both RASSF1C and RhoA expression promote the release of pro-migratory and pro-invasive EVs."*
*Fig 3f: no significant increase after addition of RhoA-EV´s when comparing to its control group (control plasmid) instead of comparing to untreated control*
We again thank the reviewer for raising this point. We have now amended the above sentence as follows.
*"Taken together, the data show that RASSF1C promotes EV functional transfer* ***and the release of pro-invasive EVs****, and that both RASSF1C and RhoA expression promote the release of pro-migratory EVs." (lines 305-306).*

*Minor points:*
*General Western Blots: no markers/sizes given in the WB data*
We thank the reviewer for highlighting this. As per EMBO Journal guidelines we provide uncropped western-blots with molecular weight markers as a "Source data" file.

*Fig 1a: scale bars missing*
We now have made the scale bars (20 µm) visible on the figure.
*p. 8 - Fig S2d: membrane blebs don´t look very prominent to me...? Possible to have video footage or better image?*

We apologise to the reviewer for not providing a clearer image of blebbing in RASSF1C-expressing cells. Now, we corrected the image (as EV2I), where arrows show blebs (rounded F-actin/pMLCII co-localized dynamic structures, typical of amoeboidly migrating cells) within the plasma membrane.

*MDA-MB-231 RASSF1C*

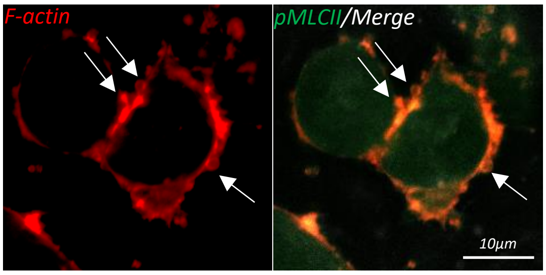


*Figure legend to Suppl. Fig 2d: "Cells were stained with F-actin (red)...". This should probably say "...were stained with phalloidin for F-actin.:"*
This has now been amended (lines 1257-1258).

*Referee#2:*

*The manuscript by Tognoli & Vlahov etc is well written and describes a new role for RASSF1C in breast cancer progression by describing a role in upregulating Rho/rock signalling axis in cancer - leading to stem like phenotypes and importantly revealing an EV based role for the transfer of a more invasion, migratory phenotype (in vitro and in vivo by intravital imaging)....were RASSF1C role via SRC can regulate these phenomenon via rock/rho signalling axis.*

*The experiment are well designed with caveats addressed in the paper and discussed.... Moreover appropriate control experiment are evident over activating/reducing expression or activity for RhoA CA/DN*
*Links to the clinical relevance are clearly show via TCGA and patient data etc and also nice work is presented via RASSF1C epigenetic control vi dCas9-VP64 in this study should be applauded. Lastly the small versus medium/large EVs speaks to novelty for this MS..I would like the authors to talk to recent work on ROCK/KD effects in breast cancer Pmyt on secretome conditional media work Involving Creld2 and discuss how this work aligns to this.- pro tumourogenic .... The author touch upon this for manipulation of the tumour microenvironment but more should be discussed re the role of targeting ROCK in this area.*
We thank the reviewer for bringing this seminal study to our attention. We have now included in our Discussion section a paragraph where the above work is discussed and related to our own study (lines 482-487).

*-Since the author describe the role for RASSF1C in SRC based control of Rock/Rho signalling via RhoGDI -can the authors perform experimets to inhibit Src either via molecular manipulation or via commonly used drugs to target Src in principle? This can be done for in vitro setting and can also be assessed in vivo to assess if Src inhibition here would reduce EV effects on surrounding cell phenotypes and migratory potential etc..this would speak to the pathways role in this disease and its potential clinical targeting. Similar as discussed above Rock targeting can be discussed in this context as a drug target.*
We thank the reviewer for raising this important point and for suggesting the above experiments

aimed at providing evidence for clinical targeting of Src. Indeed Src activation is a key feature of our current and previous work (*Vlahov, N. et al. Curr Biol 2015).*

Src oncogene has previously been described as a modulator of EV release. For instance, one recent study in colorectal cancer cells has shown that Src co-localizes with multivesicular body markers (namely Rab7 and CD63) in cells, directly interacts with ESCRT partners (namely Alix), and promotes EV production and colony formation. Both processes are impaired in the presence of Dasatinib, a Src inhibitor, suggesting Src involvement in EV secretion and tumour progression (*Hikita et al.. Sci Rep 2019).*

Another study on breast cancer cells has previously shown that Src stimulates the secretion of exosomes having pro-migratory activity on endothelial cells in a syntenin-dependent manner. In this study Src involvement in EV production was assessed through pharmaceutical inhibition and Src ability to affect exosome functionality (i.e. to induce cell migration) was tested by iRNA interference (SRC and syntenin exosomes *(Imjeti et al. PNAS 2017).*

Finally, a previous study on chronic myeloid leukaemia cells has described a role for Src-derived EVs in promoting remodelling of endothelial cells. This effect was abrogated with the use of two tyrosine kinase inhibitors (Imatinib and Dasatinib). Furthermore, the effects of Src pharmaceutical inhibition on angiogenesis induced by leukaemia cell-derived EVs were confirmed in an *in vivo* murine model (*Mineo et al. Angiogenesis 2012).*

Therefore, a role for Src in both EV secretion and EV effect on surrounding cell phenotypes has already been described. Directly inhibiting Src we would likely achieve the effects observed in previous studies, but we feel that this would not add to the novelty of the present study, which in our opinion is the activation of Src by upstream regulator, RASSF1C. Nevertheless, we feel that these are important points raised and are now discussed along with the above references in support of our model in our Discussion section (lines 450-452 and 488-492).

*Referee#3:*

*In this manuscript, the ability of the RASSF1C oncogene to affect breast cancer cell morphology, invasive behaviours and stemness has been examined. RASSF1C is expressed as a result of an epigenetic switch that represses its homologue RASSF1A, which has previously been characterized as a tumour suppressor. The expression of RASSF1C is reported to activate the SRC tyrosine kinase, leading to phosphorylation and inhibition of Rho-GDI protein, resulting in increased Rho activity. The increase in Rho activity promotes myosin light chain phosphorylation, driving cell contractility and morphological transitions from mesenchymal or eplithelial to rounded or amoeboid morphologies. In parallel, these morphological changes are associated with changes in the expression of stem cell markers, including CD133, ALDH1 and NANOG, suggesting that the morphological transformation is a driver of cancer cell stemness. The altered morphology also promotes the generation and release of extracellular vesicles that promote tumour cell migration. In vivo, cells expressing RASSF1C were more motile, and adopted a rounded mode of motility. Experiments designed to assess the transfer of material to recipient cells via extracellular vesicles revealed that RASSF1C increased this horizontal transfer, both in vitro and in vivo, likely contributing to increased invasion of the recipient cells. Analysis of gene methylation patterns revealed a reciprocal relationship between RASSF1A and RASSF1C, consistent with respective tumour-suppressive and oncogenic roles in breast cancer patients.*

*Overall, the study is interesting and logical. In general, the experiments are well designed and appropriate. Major issues include a lack of experimental replicates, quantification and statistical analysis of some experiments, particularly western blot experiments that indicate subtle responses. The relatively small changes observed are not the problem, many biological processes are governed*

*by small changes in signalling. However, given the inherent variability in some of the assays that have been used, it is important to provide data showing that the observations are reproducible and robust.*

*Points to be considered*

*1. Introduction - Why state that Rho/ROCK signalling promotes MLCK mediated MLCII phosphorylation? The evidence that ROCK kinases directly phosphorylate MLCII was reported several decades ago, and yet I cannot think of a convincing mechanism for ROCK promoting MLCK activity. Unless you are referring to a fairly old idea that MLCK is the only MLC kinase, and that ROCK operates exclusively as an inhibitor of the MLCII phosphatase via MYPT1 phosphorylation?*

We thank the reviewer for pointing out this incogruence. We fully agree with the reviewer, that the mechanism by which activated ROCK kinase directly regulates the phosphorylation of MLC2 is widly reported and a fully accepted paradigm of Rho/ROCK signalling pathway for the last 10 years. We apologise to the reviewer for not having included such a crucial finding into the introduction of our manuscript. This has now been corrected (lines 45-46).

*2. Figures 1b and 1d - the morphologies should be defined as rounded and elongated, amoeboid and mesenchymal refer to migration modes, as this assay is based purely on cell shape.*

We thank the reviewer for raising this point, we have now changed this nomenclature in both figures and text.

*3. Figure 1c - does the overexpression of RASSF1C increase pMLCII levels? Or does it just cause the staining to be re-localized? This would be easily addressed by western blotting. Is the increase in pMLCII levels sensitive to Y27632 or C3?*

We thank the reviewer for raising this point. Indeed, we have shown in Fig 2B, Fig 2G and EV2C that RASSF1C expression leads to increased pMLCII expression levels as well as its relocating into cortical actomyosin. Unfortunately, we have not provided WBs to check the sensitivity of pMLCII expression levels to Y27632 and C3 inhibitors. Instead, we have shown changes in ROCKI and MLCII expressions in MDA-MB-231 constructs with OE RhoA, constitutively active Rho (CA) (increased MLCII activity) and dominant negative Rho (DN) (downregulation of MLCII).

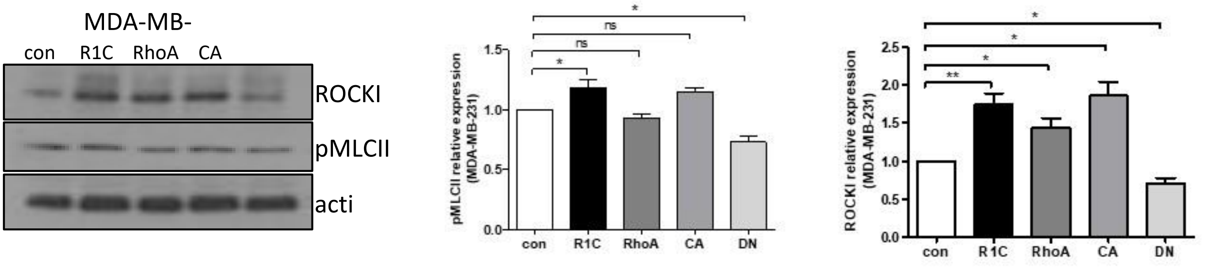


*4. Previous studies (including by the current author's studies from 2015) have shown that overexpressed RASSF1C is localized at microtubules and at the nuclear envelope. Is RASSF1C localized on microtubules in Figure 1c and 2i? If so, does the change in cell morphology have anything to do with RASSF1C microtubular localization?*

Our previous work and that of others have widely demonstrated that the RASSF1A isoform associates with microtubules during mitosis or when overexpressed in interphase cells *(Song et al. Nat Cell Bio 2004, El-Kalla et al. Oncogene 2010, Dallol et al. Cancer Res 2004)*. We have also indentified endogenous RASSF1A at the nucelar envelope (*Chatzifrangkeskou et al. EMBO J 2019)*. RASSF1C has a distinct N-terminus which does not result in similar localisation, but localises to cell-cell junctions where it promotes SRC-mediated phosphorylation of E-cadherin and induces

vesicularisation of E-cadherin, thus resulting in removal of epithelial contacts (*Vlahov, N. et al. Curr Biol 2015).*
Furthermore, in our current manuscript, we investigate cell plasticity and cell transition between different modes of invasion. This requires experiments in three-dimensional matrix to allow cells to adapt their morphology according to the mode of invasion. As we have shown in Fig 1C and 2I, RASSF1C-expressing MDA-MB-231 cells cultured in 3D collagen matrix reorganized their cytoskeleton into cortical actomyosin as a response to upregulation of Rho/ROCK/pMLCII signaling pathway, which is associated with amoeboid transition. As reported in literature (*Kolodney MS et al.*

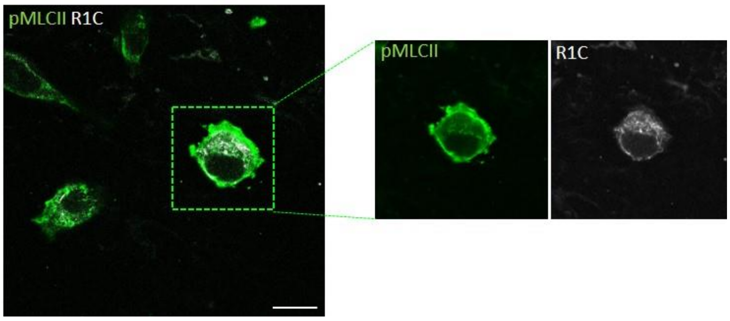


*PNAS 1995*), we suggest that contractility generated by actomyosin forces in 3D collagen lattice might be associated with microtubule disruption. In contrast to RASSF1A subcellular localisation, we observed that MDA-MB-231 cells expressing RASSF1C, cultured in 3D collagen matrix, show co-localization of RASSF1C and pMLCII in cortical actomyosin structures and not with microtubules.

*5. Figure 1d - does the knockdown of RASSF1C decrease pMLCII levels or change its localization? Does it change the distribution of F-actin from the cortical cytoskeleton to stress fibres?*
We thank the reviewer for asking us to clarify this point. As an siRNA directly targeting RASSF1C is not commercially available, we choose a more specific approach and prepared RASSF1C point mutants. Western blot analysis (Fig 2G) shows decreased expression levels of pMLCII when RASSF1C is mutated. Moreover, RASSF1C mutations lead to strong reorganization of actomyosin cytoskeleton (Fig 2I), from cortical actomyosin (in RASSF1C-expressing cells) to stress fibres (in RASSF1C mutants, R197W and R199F).

*Western blots (or qPCR) should be included for Figure 1d to show the amount of RASSF1C depletion. There is a similar absence of controls for the extent of knockdown achieved in other figures that should be rectified.*
We thank the reviewer for pointing this out. Fig 1D represents a collagen assay to assess cell morphology in three-dimensional matrix for which the representative western blot analysis is shown in Fig 2C. The siRNA has been validated and published in our previous work (*Vlahov et al. Curr Biol 2015, Fig S1B, S1C)* and we demonstrate efficiency in reduction of RASSF1C expression (Fig 2C).

We also thank the reviewer for their valid point regarding KD controls. RASSF1C siRNA is not commercially avaliable and many attempts to design specific a siRNA failed, instead we chose to employ RASSF1C point mutants (R197W and R199F), that were used throughout the manuscript (Fig 2G, 2H, 2I, EV2B-D, EV4D).

*6. Figure 1e - how was it determined that this activity is MMP9? Was a western blot done in parallel? Based on the molecular weight of the band? If so, it would be important to show the MW markers. How reproducible is this observation? Is it statistically significant?*

We apologise to the reviewer for not giving a clearer explanation of the zymography assay. Gelatin zymography is a powerful method for detection of proteolytic enzymes capable to degrade specific

substrates. The assay uses SDS-polyacrylamid gel copolymerized with gelatin and, in order to retain enzymatic activity in the samples, the samples were electrophoresed under non-reducing conditions. Upon incubation of the gel in a calcium-containing buffer, renaturated enzymes are able to degrade gelatin which can be detected as a clear band after gel staining with Comassie blue (*M.Roth and R. Fridman, Assessment of Gelatinases (MMP-2 and MMP-9) by Gelatin Zymography, Methods Mol Med. 2001*). The samples (three biological replicates) were run along with a protein marker to detect correct molecular weight of MMP9-92kDa. Now we have included quantification of zymography analysis of three biological replicates in MDA-MB-231 cells (control and RASSF1C-expressing cells, EV1I) with statistical significance and a representative picture of the raw gel after staining with Comassie blue labelled with protein marker molecular weights.

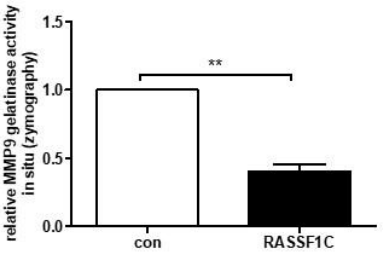


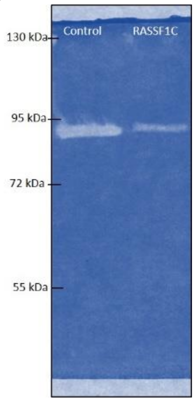


*7. Figure 1f - given that MMPs can alter cell behaviours by acting as autocrine factors that affect cell signalling pathways, it is not a given that the hydrogel experiment proves that the effect of RASSF1C is MMP independent. In fact, given the result in the left panel, it's not even clear why this experiment was done. But it would be more robust if it were shown that GM6001 did not affect invasion into hydrogel, in agreement with the lack of effect on Matrigel invasion.*

We thank the reviewer for raising this point. We fully agree with the reviewer that MMPs can act as autocrine factors that affect cell signalling pathways. However, the main function of MMPs during cell invasion is to degrade surrounding tissue and allow cells to spread into distant sites. Fig 1E, shows that MDA-MB-231 RASSF1C-expressing cells have downregulated the activity of MMP9 metalloprotease. Moreover, we provide a 3D invasion assay in non-metalloprotease degradable hydrogel which only allows invasion that is _independent_ of proteolytical degradation of ECM. These cells, which are using protrusive forces generated by cortical actomyosin, squeeze into and invade through the pores within the hydrogel matrix. The hydrogel invasion assay allowed us to elucidate that, invasion of control cells with active MMP9 (Fig 1E) is highly dependent on MMPs degradation of Matrigel matrix and indeed it is abrogated by GM6001 inhibitor (Fig 1F, left graph) and greatly inhibited by Hydrogel matrix, a substrate not degradable by MMPs (Fig 1F, right graph). However, RASSF1C-expressing cells do not use proetolytic degradation of ECM as a main mechanism during invasion, and that explains why inhibition of MMPs by GM6001 did not influence RASSF1C-expressing cell invasion in Matrigel matrix (Fig 1F, left graph). Moreover, the use of a hydrogel substrate (Fig 1F, right graph) did not influence invasion of RASSF1C-expressing cells, due to their amoeboid type of invasiveness, for which crucial actomyosin protrusive forces at the leading edges allow them to squeeze through the pores. Now, we corrected the statistics and compare Matrigel invasion in MDA-MB-231 control and RASSF1C-expressing cells with or without GM6001 inhibitor to provide a clearer insight of the point of the assay. We hope that our explanation along with Fig 1E satisfies the reviewer.

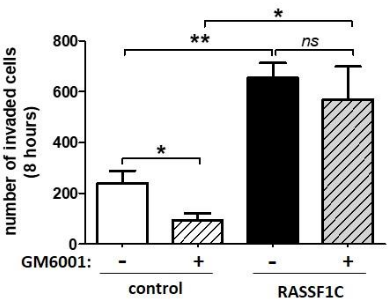

*8. Figure 1g - it would be more obvious if the labelling indicated Y27632 in the graph to be consistent with the text. C3 hasn't been labelled as RHOi, so why not just label them with the way that the experiment was actually done. Given that there are many more ROCK inhibitors than just Y27632, why not be explicit about the experiment?*

We thank the reviewer for pointing out this incongruence. We have now amended ROCKi to Y27632 throughout figures and text.

*9. Figure 1g - is the effect of Y27632 and C3 on the invasion induced by RASSF1C actually significant?*

We apologise to the reviewer for not having included statistical significance. This has now been corrected.

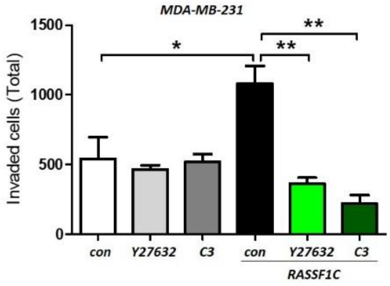


*10. Figure 1j - the cell migration speeds are indicated, the text reports that the cells expressing RASSF1C move via "more amoeboid, rounded morphology". This data is actually important and should be moved from the supplementary data to the primary figures.*

We thank the reviewer for making this suggestion. We have now moved the figure to the main panel, given its importance.

*11. Figure 2a - Which Rho protein is this? RhoA? Given the variability of the pull-down assays, this should be repeated, quantified and statistically tested.*

We thank the reviewer for raising this point. The active Rho pull-down kit used in our assays includes an anti-Rho antibody that reacts with RhoA, RhoB and RhoC. Thus, the western blot (Fig 2A) showing expression level of total Rho includes RhoA, RhoB and RhoC according to manufacturer's instructions. We also apologise to the reviewer for not having included a quantification of three biological replicates of Rho activity investigated by Western blot analysis. This is now corrected and added to EV2A.

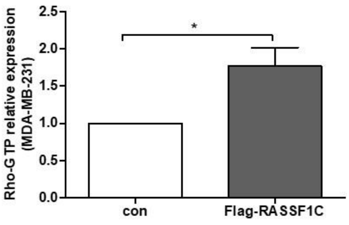


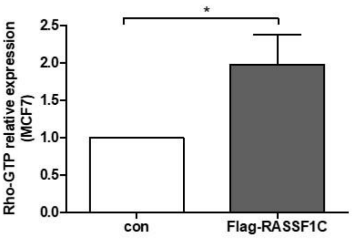

*12. Figure 2b - Are the levels of active Rho reproducible and significant? Are the differences in RhoGDI phosphorylation reproducible and significant? By eye, it's not even apparent that there are differences, which reinforces the importance of replication and quantification.*

We thank reviewer for raising this point. We believe that detected active Rho in our manuscript corresponds to both bands, as the appereance of GST affinity-purified Rho-GTP double bands have been documented in previous studies *(Sander et al. J Cell Biol 1999; Carton et al. Am J Physiol 2002*). Now we included a quantification of Rho-GTP and pRHOGDI from Western blot analysis (EV2A-B).

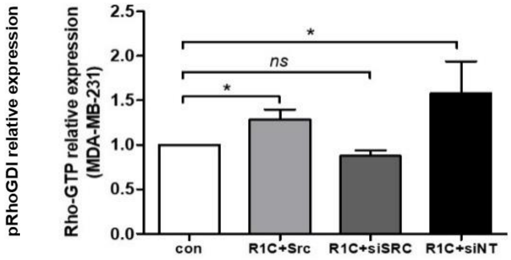


*13. Figure 2b - the levels of pMLCII between +/- RASSF1C are apparent, and this actually addresses point 3 I made above. But the effect of either SRC over-expression or siRNA mediated knockdown appear to be the same, both result in decreased pMLCII. According to the model of this paper, the decrease in pMLCII when SRC is knockdown is logical, but why would SRC over-expression decrease pMLCII? This again reinforces the need to replicate these experiments, quantify and statistically evaluate.*

We thank the reviewer for highlighting this. As stated in point 3, we show also in Fig 2G, EV2B, C that RASSF1C expression alone leads to increased pMLCII levels. We do understand the confusion about SRC OE and SRC KD, however, the siSRC condition (*lane 3*) should be compared to siNT (*lane 4*) and indeed there is a decrease in pMLCII levels upon SRC knock-down, which is in line with our working hypothesis; whereas the SRC OE condition (*lane 2*) should be directly compared to the control (empty plasmid) condition (*lane 1*), and in this case what we see is an increase of pMLCII upon SRC/R1C OE, which again is in line with our working hypothesis. We hope that this, along with Fig 2G, EV2B-C, satisfies the reviewer.

*14. Figure 2c - replicates, quantification, statistical evaluation are needed.*

We again apologize to the reviewer for not including quantification and statistical analysis of replicates of our Western blot analysis. Now, we provide quantification and statistical evaluation below (and included in EV2D). We ensure all our experiments are replicatable and statistically sound in order to conclude reliable information.

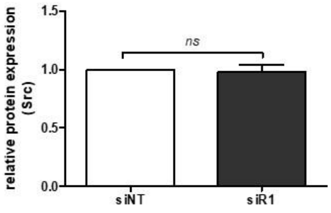

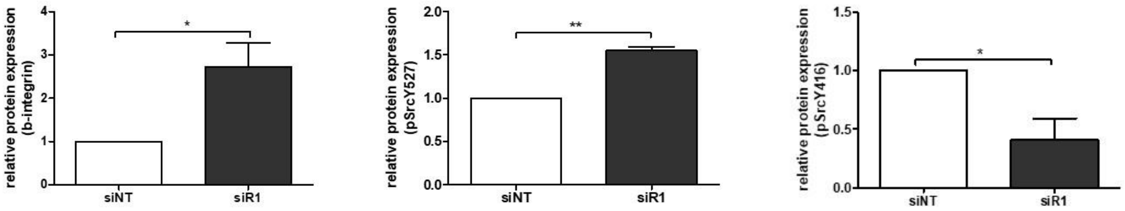


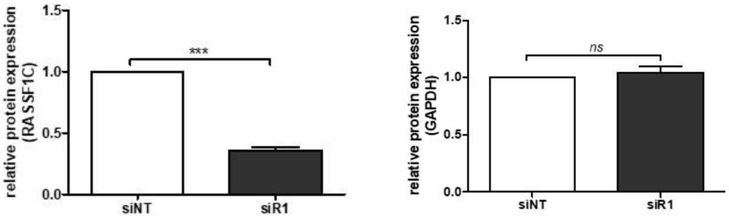


*15. Figure 2d - This experiment indicates that SRC and RhoGDI are constitutively associated? And then if HA-tagged RASSF1C is included, it can be found in the complex by blotting for the HA tag. But it doesn't actually address whether RASSF1C is needed for, or promotes, the SRC-RhoGDI interaction. Can endogenous RASSF1C be found in this complex? Does RASSF1C knockdown reduce the SRC-RhoGDI interaction. Also, please label what has been IPed on the figure, the text didn't actually state this and only the figure legend indicated what antibody has been used.*

We thank the reviewer for raising this point. In the figure, a row stating what has been IPed can be found just above the western blot images (**IP**: IgG and SRC). We have now also added this information to the main text. Our experiment indeed shows constitutive association between SRC and RhoGDI. Moreover, if exogenous RASSF1C is expressed, it can be found in the same complex. Our working hypothesis is that inclusion of RASSF1C in the complex will allow for SRC activation (i.e. SRC phosphorylation at Y416) as we previously published and also confirmed in this study (Fig 2C shows that reduction in endogenous RASSF1C levels reduced SRC activating phosphorylation, pSRC-Y416, and increased SRC inhibitory phosphorylation, pSRC-Y527).

We agree with the reviewer that immunoprecipitation of endogenous RASSF1C would give a more complete and informative answer, however, in our experience and previous work detection of endogenous RASSF1C in immunoprecipitation assays has proven difficult and therefore this is why we have resorted to exogenous expression for these assays.

*16. Figure 2f - label the IP please.*

Similar to labelling in Fig 2D, IPs are labelled appropriately to indicate where IgG or IgG-SRC was used.

*What happened in the RhoGDI blot in the control + SRC lane?*

While we do see RhoGDI in the SRC IP, the lane demonstrates background staining reflecting the poor quality antibodies beyond our control despite multiple replicates.

*Wouldn't the over-expression of RASSF1C alone be expected to increase the association of SRC with RhoGDI compared to the SRC alone according to the model? That would be predicted if the R197W and R199F expression resulted in less SRC-RhoGDI interaction. Unless the idea is that these point mutations antagonize the SRC-RhoGDI interaction? Also, is there actually less RhoGDI in the R197W lane compared to the wild-type RASSF1C? This again reinforces the need for replicates when there are such subtle differences.*

As pointed out in the previous point, we do not observe increases in SRC-RhoGDI but rather the association of RASSF1C results in activation of SRC and phosphorylation of RhoGDI. The mutations reflect an inability to couple this to Rho activation. Quantification of RhoGDI levels from the IP is now reported below and included as EV2E and it indeed shows a decrease in RhoGDI association with SRC

upon expression of either of the RASSF1C mutants.

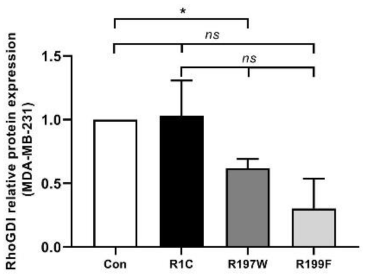


*17. Figure 2g - label the top panel please. It seems like Figures 2g and Supplemental Figure 2a are revealing a new mechanisms of action of RASSF1C, that it regulates ROCK1 protein levels. Why is this not part of the central hypothesis? How reproducible is this observation? Is it post-translational or transcriptional? The subtle effects on pMLCII should be corroborated through replicates, quantification and statistical evaluation.*

We thank the reviewer for pointing out the missing labeling in Fig 2G, which has now been corrected. We also appreciate the reviewer's suggestion regarding the mechanism of action by which RASSF1C regulates ROCK1 expression levels. We again apologise to the reviewer for not including a statistical evaluation of western blot assays for ROCK1 and MLCII protein expression related to Fig 2G and EV2C, now corrected and included below (and as EV2C and F). Our working hypothesis is explained in the schematic cartoon in Fig 2E, showing that RASSF1C controls mesenchymal-amoeboid transition via upregulation of protein expression levels of ROCK1 and MLCII, crucial proteins for amoeboidly invading cells. We agree with the reviewer that this is intruiging and deserves more in depth analysis and experimentation, but we believe that it is beyond the scope of the current, already complex, study.

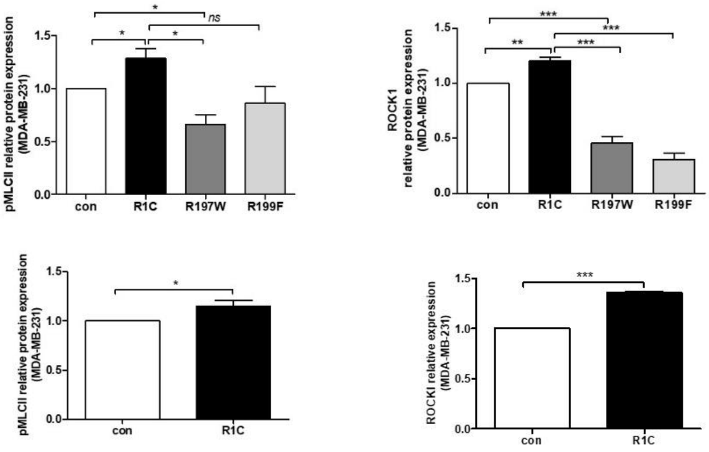


*18. Figure 2i - what are the proportions of rounded vs elongated cells for each condition? Similar to what was reported in Figure 2d.*

We thank the reviewer for asking to clarify this point. Quantification of rounded vs elongated cells, upon RASSF1C mutants expression, by using single cell morphology in 3D collagen is reported in Fig 2H. And the corresponding representative pictures can be found in EV2C. This has now been clarified also in the text. In addition to this, Fig 2I provides information on pMLCII and F-actin localisation upon RASSF1C mutants expression.

*19. Text - section headed "RASSF1C amoeboid cells release pro-invasive extracellular vesicles". In the first sentence, it is stated that RASSF1C leads to the generation of protrusive forces, but formally this has not been shown. Please revise to agree with what the data actually shows.*

We thank the reviewer for highlighting this. We have now amended the sentence in the text to reflect our data (lines 222-224).

*20. Figure 3a - data normalization is fine, but it should give an indication of the variability in the control condition as well. Otherwise, the statistical test is biased because of the artificial absence of variability in the control.*

We thank the reviewer for making this point. We have now included control conditions variability in Fig 3A and re-run the statistics test accordingly.

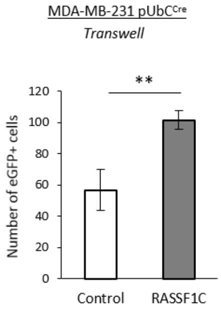


*21. Figure 3e - it should be described that this experiment is chemotaxis experiment akin to a Boyden chamber rather than migration across a 2D surface.*

We thank the reviewer for pointing this out. We have now amended the text and added an explanation to the Material & Methods section to reflect that xCELLigence is a chemotaxis transwell migration experiment (lines 674-675).

*22. Figure 4j - can the authors speculate why SOX2 was induced in EV recipient cells, but not in the RASSF1C over-expressing cells in Figure 4g? If SOX2 isn't induced, is RASSF1C really inducing stemness, or something that is "stem-like"?*

We thank the reviewer for raising this point. The two experiments however differ in their setup. In Fig 4G the Cas9 technique allows for endogenous re-expression, and not over-expression, of RASSF1C by epigenetic editing; whereas in Fig 4J secreted EVs were isolated after RASSF1C exogenous expression. The differences in SOX2 expression that the reviewer points at may stem from the two approaches used, but while SOX2 is a marker of stemness as part of the pluripotency cassette – it alone is not a bone fide marker of stem-like behaviour – whereas both OCT4 and NANOG are independently and as part of the cassette, markers of stem cells.
Furthermore, in EV4E we show that MDA-MB-231 expressing ZsGreen-RASSF1C (sorted for green$^{high}$ expression) indeed show upregulation of all three stem cell markers, NANOG, OCT4 and SOX2, compared to the sorted ZsGreen-Control.
We also support that RASSF1C induces cancer stemness via regulation of CD133 and ALDH1 (Fig 4A, 4C, 4D).

*23. This sentence from MS page 14 is confusing "In line with our data supporting a pro-oncogenic role for RASSF1C, deceased patients had less median methylation of RASSF1-2.." Does this mean lower median methylation?*

We thank the reviewer for noticing this, we have amended the text to “lower median methylation”.

*Minor points*

*1. Reviewing papers is easier if there are page and line numbers. Given the length and complexity of this manuscript, reviewing it would have been considerably easier if they had been included. I recommend it for future submissions*

We apologise for the inconvenience, lines are now numbered.

*2. The number of times that abbreviations (e.g. EMT, MAT, etc.) are defined is excessive, the first occurrence is sufficient.*

We have now corrected this.

*3. New paragraphs would be more obvious if the first line were indented, or if there was a line between it and the previous paragraph.*

*4. Consider making the colours in the graphs friendlier for people with red-green colour blindness.*

The reviewer is absolutely right, we will bear this in mind for future submissions.

## 1st Revision - Editorial Decision 25th Jun 2021

Dear Daniela, dear Eric,

Thank you for submitting your revised manuscript (EMBOJ-2021-107680R) to The EMBO Journal, as well as for your patience with our response. Your amended study was sent back to the referees #1 and #3 for re-evaluation, and we have received comments from both, which I enclose below. Please note that we editorially assessed your response to referee #2's concerns and found these to be convincingly addressed. As you will see, the other referees stated that their issues have been comprehensively resolved and they are now broadly in favour of publication, pending minor revision.

Thus, we are pleased to inform you that your manuscript has been accepted in principle for publication in The EMBO Journal.

Please consider the remaining minor issue stated by referee #1 carefully together with a number of points related to formatting and data representation as detailed below, which should be addressed at re-submission.

Further, I will share additional changes and comments from our production team during the next days to be considered.

Please contact me at any time if you have additional questions related to below points.

As you might have seen, every paper at the EMBO Journal now includes a 'Synopsis', displayed on the html and freely accessible to all readers. The synopsis includes a 'model' figure as well as 2-5 one-short-sentence bullet points that summarize the article. I would appreciate if you could provide this figure and the bullet points.

Thank you for giving us the chance to consider your manuscript for The EMBO Journal. I look forward to your final revision.

Again, please contact me at any time if you need any help or have further questions.

with
Kind regards,

Daniel

Daniel Klimmeck PhD
Senior Editor
The EMBO Journal

Formatting changes required for the revised version of the manuscript:

>> Add maximally five keywords to your manuscript.

>> Adjust the reference format to EMBO Journal style, limiting to 10 authors et al. .

>> Please add a data access section to your manuscript indicating the identifiers of the PRIDE data set and make it publically available. Also update the author checklist accordingly.

>> As to our journal policy, we kindly ask you to provide with the uncropped source data for figure EV1E.

>> Organise EV figures as one file and page per figure; limit to maximally five EV figures, and update the callouts accordingly. If you need more space these would have to go into an appendix file with Appendix Figures S1, S2...;

>> Dataset EV legends: Four movies need renaming to "Movie EV1" etc., and their legends removed from the manuscript and zipped to each movie file.

>> Source data should be uploaded as one file per figure.

>> Primer and antibodies tables should be made Table 1 and Table 2 and moved to the end of the manuscript.

********

Please remember: Digital image enhancement is acceptable practice, as long as it accurately represents the original data and conforms to community standards. If a figure has been subjected to significant electronic manipulation, this must be noted in the figure legend or in the 'Materials and Methods' section. The editors reserve the right to request original versions of figures and the original images that were used to assemble the figure.

Further information is available in our Guide For Authors:
https://www.embopress.org/page/journal/14602075/authorguide

The revision must be submitted online within 90 days; please click on the link below to submit the revision online before 23rd Sep 2021.

Link Not Available

------------------------------------------------

Referee #1:

The authors have done a good job addressing my concerns. One minor error is in Figure EV1C: control and RASSF1C is probably mixed up?

Referee #3:

The manuscript was carefully reviewed by three reviewers during the first submission. The authors

have done a thorough job of responding to the reviewers' comments, and I am happy to recommend acceptance. The central findings were very interesting, my major concerns were that there were insufficient experimental replication and statistical analysis in the first submission. These shortcomings have been satisfactorily addressed.

The authors performed the requested editorial changes.

## 3rd Revision - Editorial Decision 27th Jul 2021

Dear Daniela, dear Eric,

Thank you for submitting the revised version of your manuscript. I have now evaluated your amended manuscript and concluded that the remaining minor concerns have been sufficiently addressed.

Thus, I am pleased to inform you that your manuscript has been accepted for publication in the EMBO Journal.

Please note that it is EMBO Journal policy for the transcript of the editorial process (containing referee reports and your response letter) to be published as an online supplement to each paper. I would thus like to ask for your consent on keeping the additional referee figures included in this file.

Also, in case you might NOT want the transparent process file published at all, you will also need to inform us via email immediately. More information is available here:
http://emboj.embopress.org/about#Transparent_Process

For the data availability on your study, please make sure that privacy is released from the PRIDE dataset and it is made available online. Please note that we will need this in order to move on with online publication of the article.

------------------------------------------------

Please note that in order to be able to start the production process, our publisher will need and contact you regarding the following forms:

- PAGE CHARGE AUTHORISATION (For Articles and Resources)
http://onlinelibrary.wiley.com/journal/10.1002/(ISSN)1460-2075/homepage/tej_apc.pdf

- LICENCE TO PUBLISH (for non-Open Access)

Your article cannot be published until the publisher has received the appropriate signed license agreement. Once your article has been received by Wiley for production you will receive an email from Wiley's Author Services system, which will ask you to log in and will present them with the appropriate license for completion.

- LICENCE TO PUBLISH for OPEN ACCESS papers

Authors of accepted peer-reviewed original research articles may choose to pay a fee in order for their published article to be made freely accessible to all online immediately upon publication. The EMBO Open fee is fixed at $5,200 (+ VAT where applicable).

We offer two licenses for Open Access papers, CC-BY and CC-BY-NC-ND.
For more information on these licenses, please visit: http://creativecommons.org/licenses/by/3.0/ and http://creativecommons.org/licenses/by-nc-nd/3.0/deed.en_US

- PAYMENT FOR OPEN ACCESS papers

You also need to complete our payment system for Open Access articles. Please follow this link and select EMBO Journal from the drop down list and then complete the payment process: https://authorservices.wiley.com/bauthor/onlineopen_order.asp

Should you be planning a Press Release on your article, please get in contact with embojournal@wiley.com as early as possible, in order to coordinate publication and release dates.

On a different note, I would like to alert you that EMBO Press is currently developing a new format for a video-synopsis of work published with us, which essentially is a short, author-generated film explaining the core findings in hand drawings, and, as we believe, can be very useful to increase visibility of the work. This has proven to offer a nice opportunity for exposure i.p. for the first author(s) of the study. Please see the following link for representative examples and their integration intob the article web page:
https://www.embopress.org/video_synopses
https://www.embopress.org/doi/full/10.15252/embj.2019103932

Please let me know, should you be interested to engage in commissioning a similar video synopsis for your work. According operation instructions are available and intuitive.

Finally, we have noted that the submitted version of your article is also posted on the preprint platform bioRxiv. We would thus appreciate if you could alert bioRxiv on the acceptance of this manuscript at The EMBO Journal in order to allow for an update of the entry status. Thank you in advance!

If you have any questions, please do not hesitate to call or email the Editorial Office.

Thank you again for this contribution to The EMBO Journal and congratulations on a successful publication! Please consider us again in the future for your most exciting work.

Kind regards,

Daniel

Daniel Klimmeck, PhD
Senior Editor
The EMBO Journal
EMBO
Postfach 1022-40
Meyerhofstrasse 1
D-69117 Heidelberg
contact@embojournal.org
Submit at: http://emboj.msubmit.net

EMBO PRESS

YOU MUST COMPLETE ALL CELLS WITH A PINK BACKGROUND ➔

PLEASE NOTE THAT THIS CHECKLIST WILL BE PUBLISHED ALONGSIDE YOUR PAPER

| |
|---|
| Corresponding Author Name: Daniela Pankova; Eric O'Neill |
| Journal Submitted to: Embo Journal |
| Manuscript Number: EMBOJ-2021-107680R1 |

## Reporting Checklist For Life Sciences Articles (Rev. June 2017)

This checklist is used to ensure good reporting standards and to improve the reproducibility of published results. These guidelines are consistent with the Principles and Guidelines for Reporting Preclinical Research issued by the NIH in 2014. Please follow the journal's authorship guidelines in preparing your manuscript.

### A- Figures

### 1. Data

### The data shown in figures should satisfy the following conditions:

- the data were obtained and processed according to the field's best practice and are presented to reflect the results of the experiments in an accurate and unbiased manner.
- figure panels include only data points, measurements or observations that can be compared to each other in a scientifically meaningful way.
- graphs include clearly labeled error bars for independent experiments and sample sizes. Unless justified, error bars should not be shown for technical replicates.
- if n< 5, the individual data points from each experiment should be plotted and any statistical test employed should be justified
- Source Data should be included to report the data underlying graphs. Please follow the guidelines set out in the author ship guidelines on Data Presentation.

### 2. Captions

### Each figure caption should contain the following information, for each panel where they are relevant:

- a specification of the experimental system investigated (eg cell line, species name).
- the assay(s) and method(s) used to carry out the reported observations and measurements
- an explicit mention of the biological and chemical entity(ies) that are being measured.
- an explicit mention of the biological and chemical entity(ies) that are altered/varied/perturbed in a controlled manner.

- the exact sample size (n) for each experimental group/condition, given as a number, not a range;
- a description of the sample collection allowing the reader to understand whether the samples represent technical or biological replicates (including how many animals, litters, cultures, etc.).
- a statement of how many times the experiment shown was independently replicated in the laboratory.
- definitions of statistical methods and measures:
  - common tests, such as t-test (please specify whether paired vs. unpaired), simple χ2 tests, Wilcoxon and Mann-Whitney tests, can be unambiguously identified by name only, but more complex techniques should be described in the methods section;
  - are tests one-sided or two-sided?
  - are there adjustments for multiple comparisons?
  - exact statistical test results, e.g., P values = x but not P values < x;
  - definition of 'center values' as median or average;
  - definition of error bars as s.d. or s.e.m.

Any descriptions too long for the figure legend should be included in the methods section and/or with the source data.

**In the pink boxes below, please ensure that the answers to the following questions are reported in the manuscript itself. Every question should be answered. If the question is not relevant to your research, please write NA (non applicable). We encourage you to include a specific subsection in the methods section for statistics, reagents, animal models and human subjects.**

## USEFUL LINKS FOR COMPLETING THIS FORM

http://www.antibodypedia.com
http://1degreebio.org
http://www.equator-network.org/reporting-guidelines/improving-bioscience-research-repor

http://grants.nih.gov/grants/olaw/olaw.htm
http://www.mrc.ac.uk/Ourresearch/Ethicsresearchguidance/Useofanimals/index.htm
http://ClinicalTrials.gov
http://www.consort-statement.org
http://www.consort-statement.org/checklists/view/32-consort/66-title
http://www.equator-network.org/reporting-guidelines/reporting-recommendations-for-tumo
http://datadryad.org
http://figshare.com
http://www.ncbi.nlm.nih.gov/gap
http://www.ebi.ac.uk/ega
http://biomodels.net/
http://biomodels.net/miriam/
http://jjj.biochem.sun.ac.za
http://oba.od.nih.gov/biosecurity/biosecurity_documents.html
http://www.selectagents.gov/

## B- Statistics and general methods

Please fill out these boxes ➔ (Do not worry if you cannot see all your text once you press return)

| | |
|---|---|
| 1.a. How was the sample size chosen to ensure adequate power to detect a pre-specified effect size? | All experiments were performed at a minimum of biological triplicates and the data was reported as means with representative images. In accordance with ethical guidelines for the use of experimental animals, the minimum number of mice was used. |
| 1.b. For animal studies, include a statement about sample size estimate even if no statistical methods were used. | Sample sizes for intravital imaging experiments were determined based on previous studies and we used a minimum of 4 mice per condition. |
| 2. Describe inclusion/exclusion criteria if samples or animals were excluded from the analysis. Were the criteria pre-established? | For the intravital imaging experiments, mice and imaging positions were excluded if the same tumor areas could not be visualized for the full duration of the imaging. |
| 3. Were any steps taken to minimize the effects of subjective bias when allocating animals/samples to treatment (e.g. randomization procedure)? If yes, please describe. | Animals were randomly assigned to the indicated cancer cell transplantation groups. Researchers were not blinded to the group allocation, however and all mice, samples and data were processed in the same manner, independent of the experimental condition. |
| For animal studies, include a statement about randomization even if no randomization was used. | Animals were randomly assigned to the indicated cancer cell transplantation experiments and groups. |
| 4.a. Were any steps taken to minimize the effects of subjective bias during group allocation or/and when assessing results (e.g. blinding of the investigator)? If yes please describe. | All mice, samples and data were processed in the same manner independent of the experimental condition. Researchers were not blinded to the group allocation. |
| 4.b. For animal studies, include a statement about blinding even if no blinding was done | All mice, samples and data were processed in the same manner independent of the experimental condition. Researchers were not blinded to the group allocation. |
| 5. For every figure, are statistical tests justified as appropriate? | Yes, we have included statistical tests with every figure and described sample size and the statistical tests used in every corresponding figure legend. |
| Do the data meet the assumptions of the tests (e.g., normal distribution)? Describe any methods used to assess it. | Data was tested for normal distribution using the D'Agostino and Pearson omnibus normality test. For normally distributed data, the unpaired t-test was used. For not normally distributed data, the Mann-Whitney test was performed. For paired measurements with not normally distributed data, the Wilcoxon matched pair test was used. |

| | |
|---|---|
| Is there an estimate of variation within each group of data? | For the MDA-MB-231 RASSF1C line the variation was not known since these were the first tumour measurement and imaging ever. |
| Is the variance similar between the groups that are being statistically compared? | Yes, the variance is similar between the groups that are statistically tested. |

### C- Reagents

| | |
|---|---|
| 6. To show that antibodies were profiled for use in the system under study (assay and species), provide a citation, catalog number and/or clone number, supplementary information or reference to an antibody validation profile. e.g., Antibodypedia (see link list at top right), 1DegreeBio (see link list at top right). | listed in manuscript |
| 7. Identify the source of cell lines and report if they were recently authenticated (e.g., by STR profiling) and tested for mycoplasma contamination. | N/A |

* for all hyperlinks, please see the table at the top right of the document

### D- Animal Models

| | |
|---|---|
| 8. Report species, strain, gender, age of animals and genetic modification status where applicable. Please detail housing and husbandry conditions and the source of animals. | NOD.CB17-Prkdcscid/NCrCr female mice of 8-20 weeks old were equally divided over experimental conditions. Mice were socially housed under standard housing conditions and received ad libitum food and water. |
| 9. For experiments involving live vertebrates, include a statement of compliance with ethical regulations and identify the committee(s) approving the experiments. | Experiments were carried out in accordance with the guidelines of, and approved by, the animal Welfare Committee of the Royal Netherlands Academy of arts and Sciences, the Netherlands. |
| 10. We recommend consulting the ARRIVE guidelines (see link list at top right) (PLoS Biol. 8(6), e1000412, 2010) to ensure that other relevant aspects of animal studies are adequately reported. See author guidelines, under 'Reporting Guidelines'. See also: NIH (see link list at top right) and MRC (see link list at top right) recommendations. Please confirm compliance. | We have reported all relevant aspects adequately. |

### E- Human Subjects

| | |
|---|---|
| 11. Identify the committee(s) approving the study protocol. | N/A |
| 12. Include a statement confirming that informed consent was obtained from all subjects and that the experiments conformed to the principles set out in the WMA Declaration of Helsinki and the Department of Health and Human Services Belmont Report. | N/A |
| 13. For publication of patient photos, include a statement confirming that consent to publish was obtained. | N/A |
| 14. Report any restrictions on the availability (and/or on the use) of human data or samples. | N/A |
| 15. Report the clinical trial registration number (at ClinicalTrials.gov or equivalent), where applicable. | N/A |
| 16. For phase II and III randomized controlled trials, please refer to the CONSORT flow diagram (see link list at top right) and submit the CONSORT checklist (see link list at top right) with your submission. See author guidelines, under 'Reporting Guidelines'. Please confirm you have submitted this list. | N/A |
| 17. For tumor marker prognostic studies, we recommend that you follow the REMARK reporting guidelines (see link list at top right). See author guidelines, under 'Reporting Guidelines'. Please confirm you have followed these guidelines. | N/A |

### F- Data Accessibility

| | |
|---|---|
| 18: Provide a "Data Availability" section at the end of the Materials & Methods, listing the accession codes for data generated in this study and deposited in a public database (e.g. RNA-Seq data: Gene Expression Omnibus GSE39462, Proteomics data: PRIDE PXD000208 etc.) Please refer to our author guidelines for 'Data Deposition'.<br><br>Data deposition in a public repository is mandatory for:<br>a. Protein, DNA and RNA sequences<br>b. Macromolecular structures<br>c. Crystallographic data for small molecules<br>d. Functional genomics data<br>e. Proteomics and molecular interactions | Proteomics data: PXD026226 |
| 19. Deposition is strongly recommended for any datasets that are central and integral to the study; please consider the journal's data policy. If no structured public repository exists for a given data type, we encourage the provision of datasets in the manuscript as a Supplementary Document (see author guidelines under 'Expanded View' or in unstructured repositories such as Dryad (see link list at top right) or Figshare (see link list at top right). | N/A |
| 20. Access to human clinical and genomic datasets should be provided with as few restrictions as possible while respecting ethical obligations to the patients and relevant medical and legal issues. If practically possible and compatible with the individual consent agreement used in the study, such data should be deposited in one of the major public access-controlled repositories such as dbGAP (see link list at top right) or EGA (see link list at top right). | N/A |
| 21. Computational models that are central and integral to a study should be shared without restrictions and provided in a machine-readable form. The relevant accession numbers or links should be provided. When possible, standardized format (SBML, CellML) should be used instead of scripts (e.g. MATLAB). Authors are strongly encouraged to follow the MIRIAM guidelines (see link list at top right) and deposit their model in a public database such as Biomodels (see link list at top right) or JWS Online (see link list at top right). If computer source code is provided with the paper, it should be deposited in a public repository or included in supplementary information. | N/A |

### G- Dual use research of concern

| | |
|---|---|
| 22. Could your study fall under dual use research restrictions? Please check biosecurity documents (see link list at top right) and list of select agents and toxins (APHIS/CDC) (see link list at top right). According to our biosecurity guidelines, provide a statement only if it could. | No |
